# Supplementary material for: Transcriptomic architecture of nuclei in the marmoset CNS
Source: Nat Commun. 2022 Sep 21;13:5531. doi: 10.1038/s41467-022-33140-z (PMC9492672; doi:10.1038/s41467-022-33140-z)
Supplement: Supplementary file 1 — Supplementary Information [file 41467_2022_33140_MOESM1_ESM.pdf]

Transcriptomic architecture of nuclei in the marmoset CNS

Jing-Ping Lin[1], Hannah M. Kelly[1], Yeajin Song[1], Riki Kawaguchi[2],
Daniel H. Geschwind[2,3], Steven Jacobson[4], Daniel S. Reich[1]*

[1]Translational Neuroradiology Section, National Institute of Neurological Disorders and Stroke, National Institute of Health, Bethesda, MD. [2]Psychiatry, Semel Institute for Neuroscience and Human Behavior, David Geffen School of Medicine, University of California, Los Angeles, Los Angeles, CA. [3]Departments of Neurology and Human Genetics, University of California, Los Angeles, Los Angeles, CA. [4]Viral Immunology Section, National Institute of Neurological Disorders and Stroke, National Institute of Health, Bethesda, MD.
*Correspondence to: Daniel S. Reich (daniel.reich@nih.gov).

Supplementary Fig. 1–43
Supplementary References

SupFig. 1 - Related to Fig1

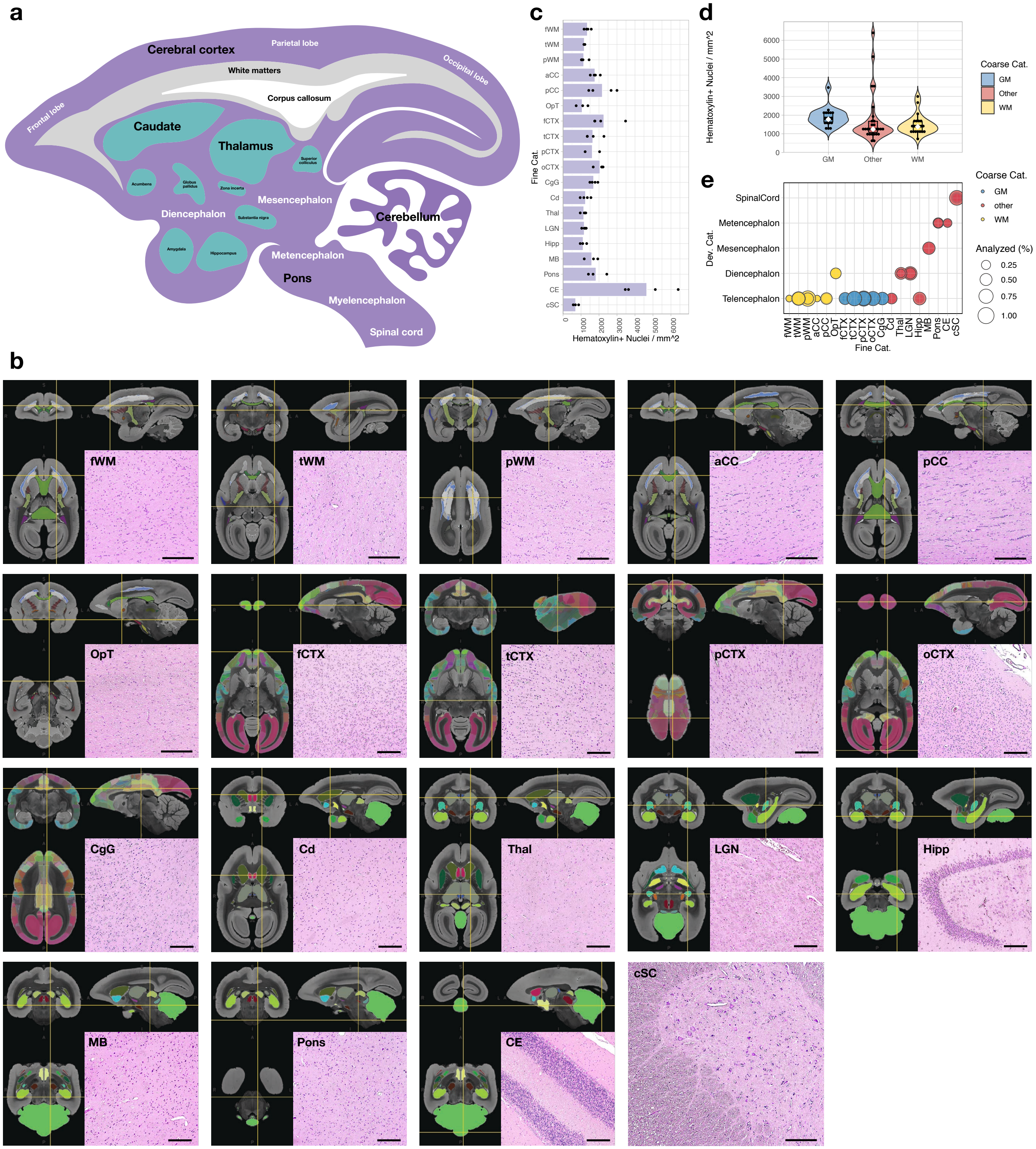

Supplementary Fig. 1. Related to Fig. 1. Location reference for image guided tissue sampling and quantification of nuclei density per area.

a. Sagittal schematic illustration of an adult marmoset brain.

b. Hematoxylin and eosin staining of 5 μm-thick tissue sections from areas as indicated on the marmoset brain mapping MRI atlas (https://marmosetbrainmapping.org/). Sections were selected by matching with regions that were sampled for snRNA-seq. **fWM**, frontal white matter; **tWM**, temporal white matter; **pWM**, parietal white matter; **aCC**, anterior corpus callosum; **pCC**, posterior corpus callosum; **OpT**, optic tract; **fCTX**, frontal cortex; **tCTX**, temporal cortex; **pCTX**, parietal cortex; **oCTX**, occipital cortex; **CgG**, cingulate gyrus; **Cd**, caudate; **Thal**, thalamus; **LGN**, lateral geniculate nucleus; **Hipp**, hippocampus; **MB**, midbrain; **CE**, cerebellum; **cSC**, cervical spinal cord. Scale bar, 200 μm. Experiment was repeated independently for 2–4 times with similar results for each CNS region as quantified in Supplementary Fig. 1c.

c. Bar plot of hematoxylin$^+$ nuclei density from each region (category IL06_tissue.3, fine annotation) that were quantified from 2–4 animals (CJaT01, CJaV02, CJaB03, and CJaD04).

d. Violin plot of hematoxylin$^+$ nuclei density in each tissue type (coarse category). "**WM**" includes fWM, tWM, pWM, aCC, pCC, and OpT; "**GM**" includes fCTX, tCTX, pCTX, oCTX, and CgG; "**other**" includes Cd, Thal, LGN, Hipp, MB, Pons, CE, and cSC. n = 42 independent samples; median is annotated (white diamond shape).

e. Dot plot of estimated nuclei recovery as a percentage of each snRNA-seq sample from each tissue type (developmental category). Each circle represents the percentage from one sample. "**Telencephalon**" includes fWM, tWM, pWM, aCC, pCC, fCTX, tCTX, pCTX, oCTX, CgG, Cd, Hipp; "**Diencephalon**" includes OpT, Thal, LGN; "**Mesencephalon**" includes MB; "**Metencephalon**" includes Pons and CE; "**Spinal cord**" includes cSC.

SupFig. 2 - Related to Fig1

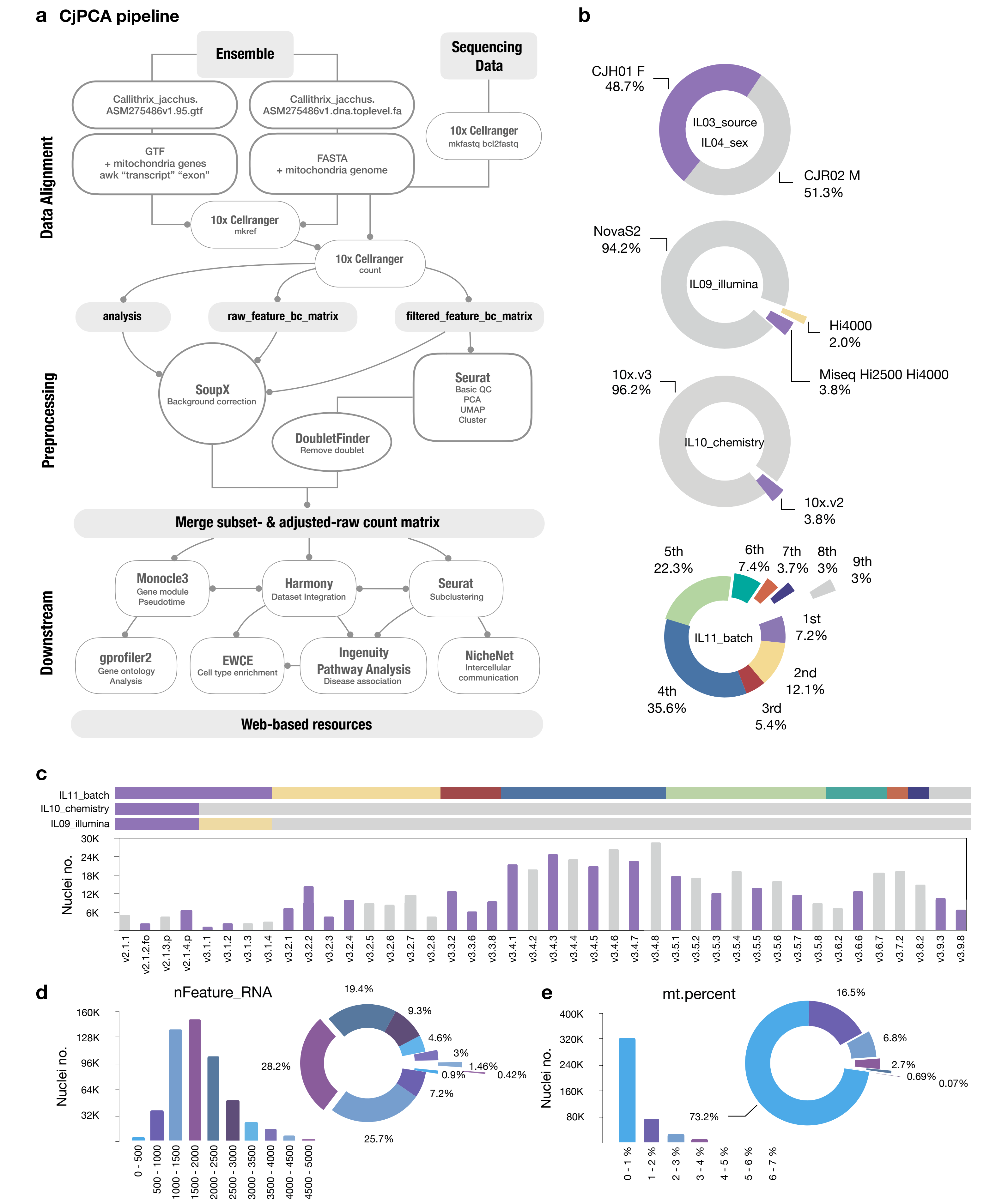

Supplementary Fig. 2. Related to Fig. 1. Workflow of data processing and quantification of nuclei number over each covariate.

- **a.** A genome assembly (ASM275486v1) and annotation were acquired from the Ensembl release-95, and the marmoset mitochondrial sequences (NC_025586.1, GenBank) were manually added to the file. An alignment reference package that was tailored for snRNA- seq was generated by 10x Cellranger mkref function. Raw count matrices were generated by 10x Cellranger count function. For individual samples, the output of 10x Cellranger was then processed in parallel with SoupX to remove ambient RNA, and with Seurat v3 followed by DoubletFinder to remove low quality nuclei and putative doublets. The adjusted matrices of each sample from SoupX output were subset by nuclei names that passed the Seurat v3 and DoubletFinder quality control step to become matrices used for the Level 1 partition. Adjusted matrices from individual samples were combined and integrated using Seurat v3 and Harmony. Monocle, gprofiler2, Expression Weighted Celltype Enrichment (EWCE), Ingenuity Pathway Analysis (IPA) software, and NicheNet algorithms were used for downstream analysis.
- **b.** Donut plots showing the proportional nuclei input from each animal and sex, sequencer, 10x chemistry version, and library preparation batch.
- **c.** Bar plot showing the input of nuclei number from each sample included in the Level 1 analysis. The color bars correspond to the labels showing in Supplementary Fig. 3b.
- **d.** Bar and donut plots showing the distribution of nuclei over the number of RNA features detected per nucleus. Nuclei were grouped into 10 bins with step size of 500 features (left). Proportional nuclei from each bin in percentage (right).
- **e.** Bar and donut plots showing the distribution of nuclei over the percentage of reads that were mapped to mitochondria genome detected per nucleus. Nuclei were grouped into 7 bins with step size of 1% (left). Percentage of nuclei in each bin (right).

SupFig. 3 - Related to Fig1

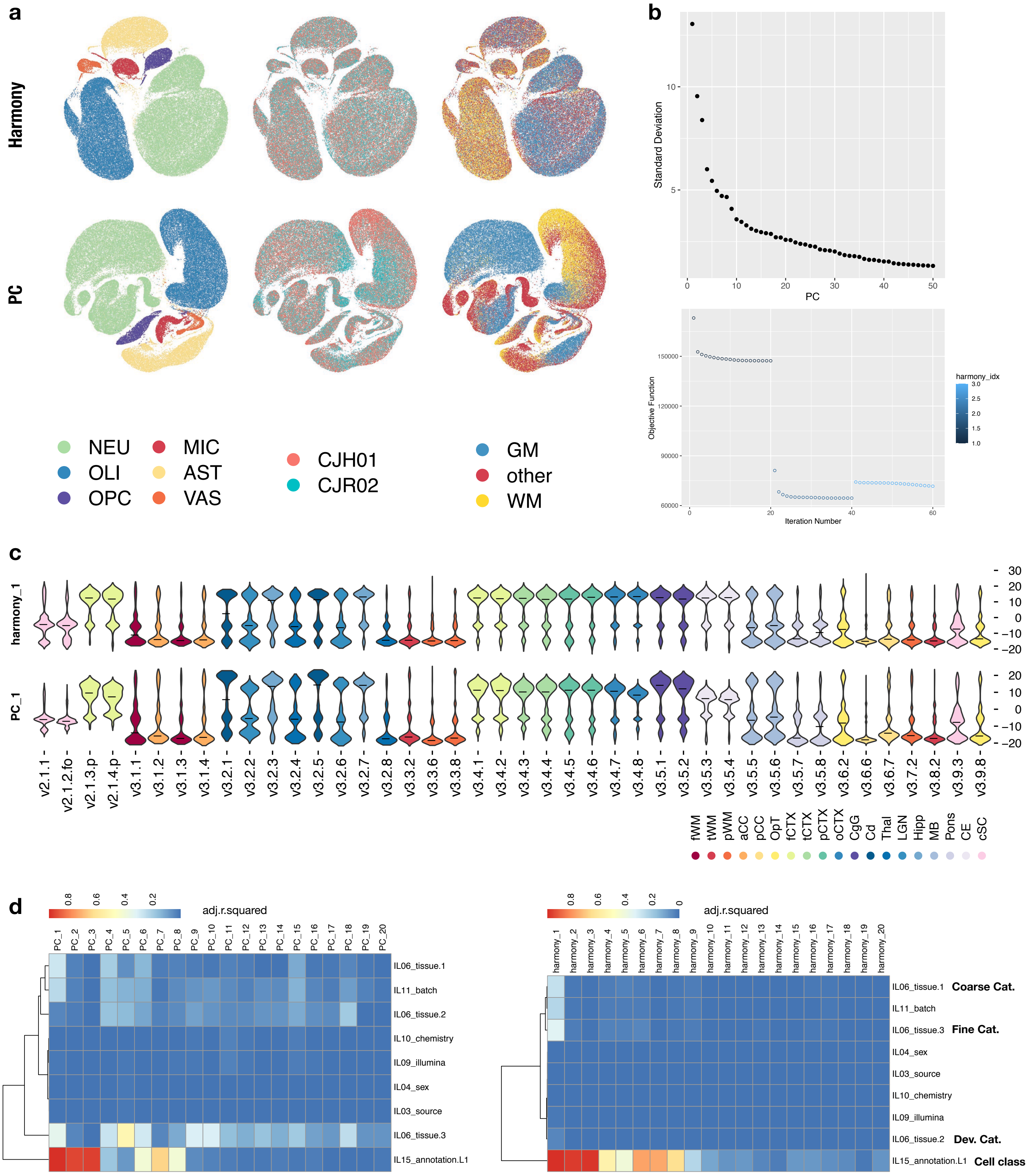

Supplementary Fig. 3. Related to Fig. 1. Basic feature assessment of individual samples before data integration and correlation of the covariate with the top 20 PC and Harmony embeddings.

a. Global UMAP scatter plot before (bottom row) and after (top row) alignment with Harmony, colored by L1 cell class (left), animal source (middle), and coarse tissue type (right).

b. Top, elbow plot showing individual standard deviation of the top 50 principal components (PC) in the Level 1 analysis. Convergence plot showing that Harmony converged after 3 iterations.

c. Violin plots showing the nuclei density along the PC_1 and Harmony_1 embedding axes. The distributions of nuclei are aligned across samples acquired from similar regions after Harmony. The color of each violin denotes the tissue of origin, as shown in the index at the bottom. Median is annotated (−).

d. Heatmap showing the linear correlations (adjusted r^2) between the top 20 principal component (PC, left) and Harmony (right) embeddings and each sample category. Level 1 cell partition results (MIC, OPC, OLI, AST, VAS, NEU) were labeled as IL15_annotation category.

See also Supplementary Data 1.

SupFig. 4 - Related to Fig1

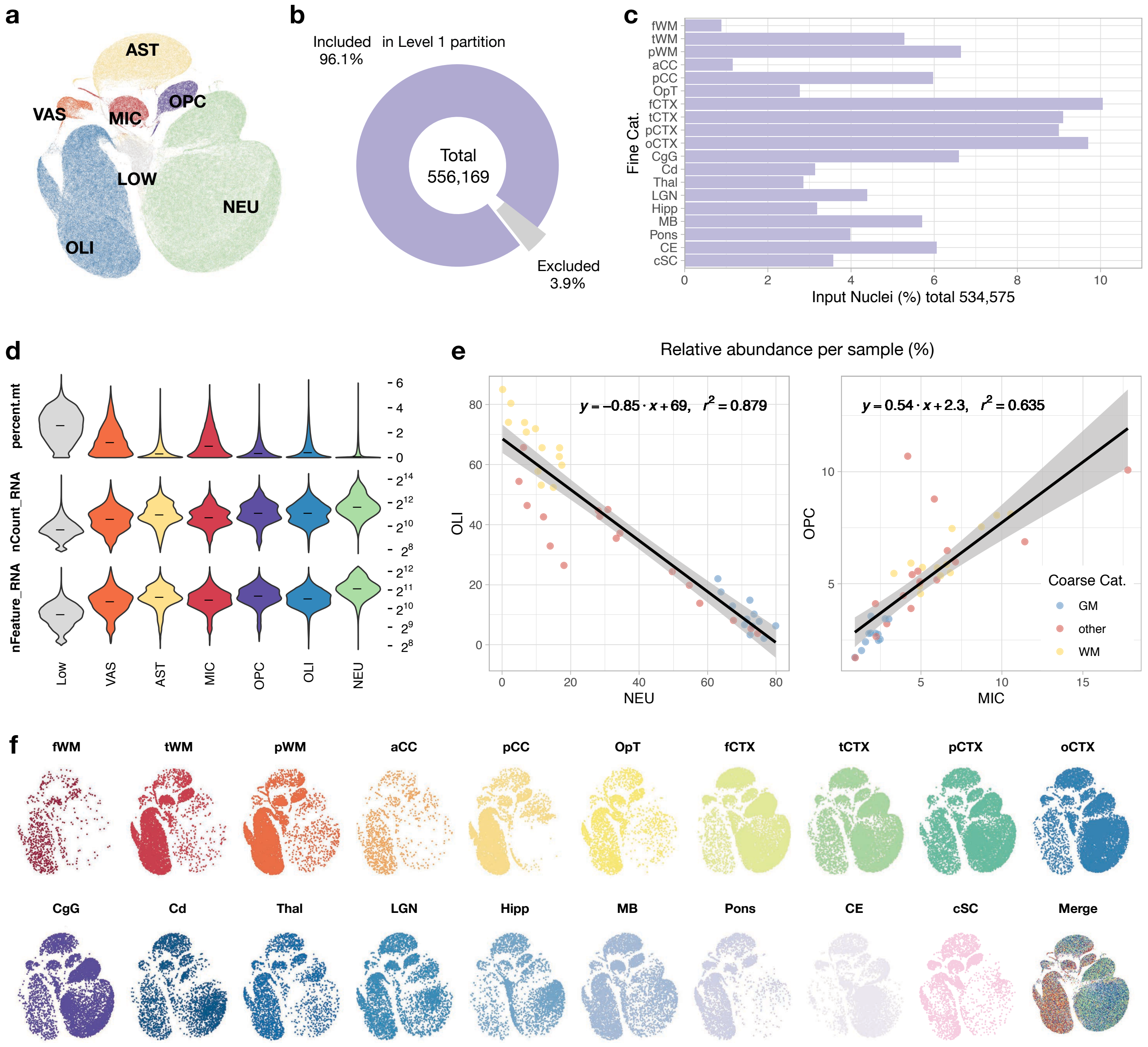

Supplementary Fig. 4. Related to Fig. 1. Basic feature assessment of Level 1 partition.

- **a.** Global UMAP scatter plot visualization of 556,169 nuclei after SoupX and DoubletFinder preprocessing steps. The UMAP space and nearest neighbor analyses were calculated from the top 5 Harmony embeddings. A "low quality" cluster was annotated and removed from further Level 1 analysis. The "low quality" cluster was informed by clustering multiple times with different parameters that met exclusion criteria, including high percentage of reads mapped to mitochondria genome, low RNA counts and features, and expressed genes that mapped to multiple canonical markers of different cell types.
- **b.** Donut plot showing the percentage of nuclei annotated as "low quality" and excluded from further analysis.
- **c.** Bar plot showing the nuclei input percentage of each tissue type in level 1 partition, where 2–3 samples were pooled from 2 animals per tissue type.
- **d.** Violin plots showing the percentage of mitochondrial derived RNA reads (percent.mt), the number of RNA detected (nCount_RNA), and the number of RNA species

(nFeature_RNA) in each partition. Median is annotated (–).

- **e.** Linear regression on the relative abundance of Level 1 annotated nuclei, showing strong to moderate correlations between OLI and NEU (left) and oligodendrocyte progenitor cells (OPC) and microglia (MIC) (right). Percentage of nuclei type per sample was calculated by normalizing to their initial nuclei input. Individual samples were colored by tissue type in

coarse category (see Fig. 1d for the full list).

- **f.** Global UMAP scatter plot visualization of 100K nuclei split by tissue types. The same object was used to make plots showed in Fig. 1e. As expected, white matter contains large numbers of oligodendrocytes (OLI) and gray matter large numbers of neurons (NEU).

SupFig. 5 - Related to Fig1

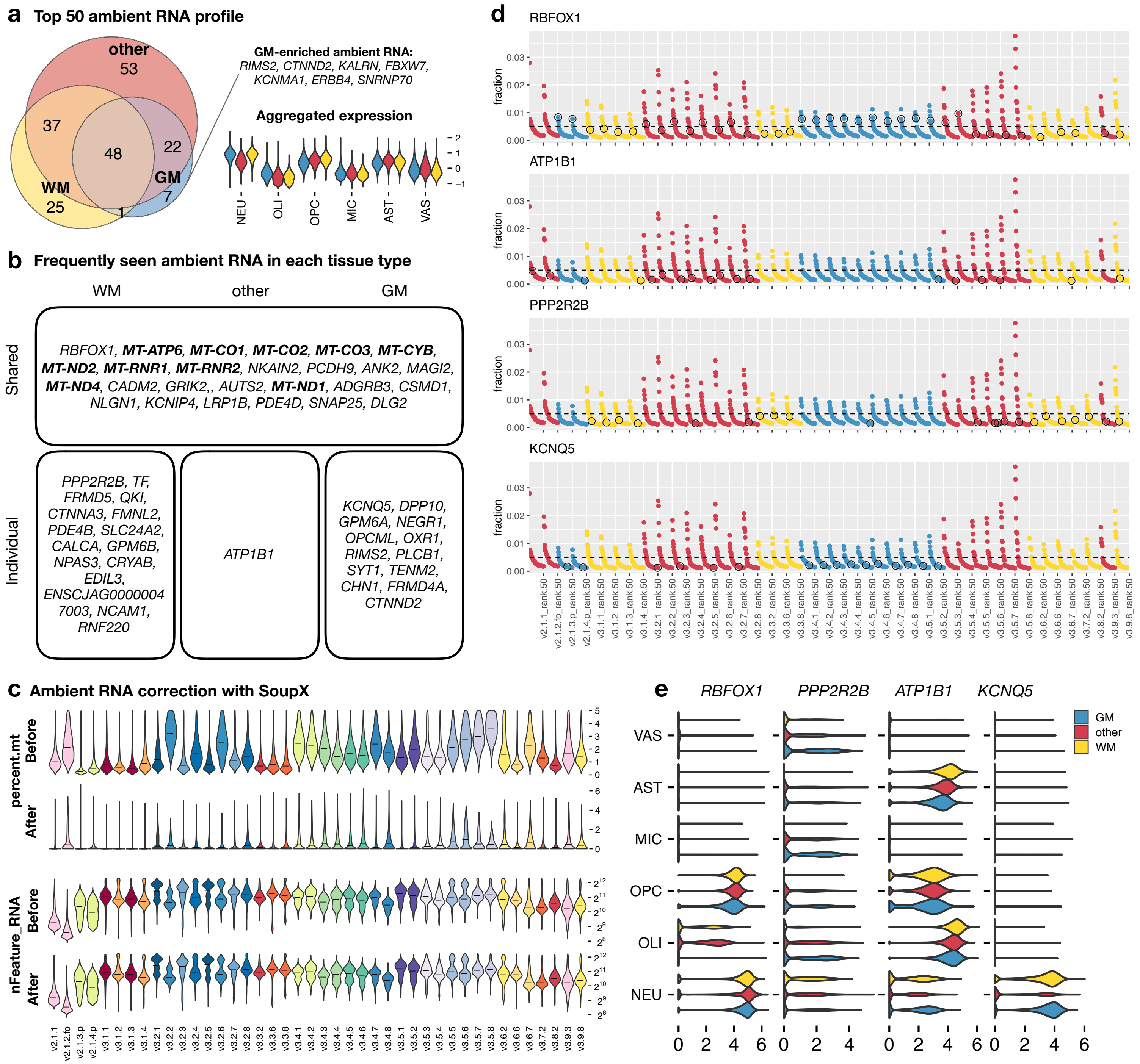

Supplementary Fig. 5. Related to Fig. 1. No systematic tissue type-specific contamination from ambient RNA was detected after background correction with SoupX.

- **a.** Left, profile of the top 50 ambient RNA transcripts in each sample, pooled and grouped by tissue type. More than 50% of these transcripts are shared across tissue type. Right, aggregated expression of 7 transcripts unique to GM presented as violin plots to assess the performance of SoupX in background correction. There is no preferentially high level of GM-enriched ambient RNA in any cell class of GM compared to other tissue types.
- **b.** The profile of frequently detected (>50% detection rate in the samples of each tissue type) ambient RNA in each tissue type. As expected, ambient RNA mapped to the mitochondrial genome is the major source of background signal across samples.
- **c.** Violin plots showing the percentage of mitochondrial derived RNA reads (percent.mt), and the number of RNA species (nFeature_RNA), before and after SoupX correction. Median is annotated (–). Top: to assess the performance of SoupX correction, the percentage of transcripts that mapped to mitochondrial genome in each sample. After correction, the median levels of mitochondrial transcripts are below 0.5% and near zero for all samples, demonstrating the effectiveness of background correction. Bottom, to demonstrate that the correction does not adversely impact the gross transcriptomic landscape, we plotted the number of detected transcripts and density before and after SoupX. The distribution was conserved, indicating that the performance of SoupX was as expected.
- **d.** The profile of ranked contribution of top 50 ambient RNA to total ambient RNA pool in each sample colored and grouped by tissue type (X-axis). The Y-axis indicates the cumulative fraction of counts for each transcript relative to the total number of transcripts found in all analyzed empty droplets. The open block circle of each panel indicates the level and its relative impact of the indicated ambient transcripts on each sample (D). We selected 1 shared transcript with differential detection between tissue types (*RBFOX1*) and 3 tissue type-specific transcripts (*ATP1B1*, *PPP2R2B*, *KCNQ5*) to demonstrate the performance of SoupX correction.
- **e.** Violin plots showing the expression level of 4 differentially detected ambient transcripts after SoupX correction. None of the 4 transcripts was preferentially detected in all cell classes across tissue types.

SupFig. 6 - Related to Fig1

a **Cumulative percentage of transcripts contributed by top-expressed neuronal or oligodendroglial genes in each cell class**

Top 20 expressed transcripts of NEU: *KCNIP4*, *RBFOX1*, *CADM2*, *PCDH9*, *NRXN3*, *CSMD1*, *FAM155A*, *NRG3*, *CNTNAP2*, *NRXN1*, *ADGRB3*, *DLG2*, *FGF14*, *TENM2*, *LRP1B*, *NEGR1*, *MAGI2*, *KCNQ5*, *DPP10*, and *OPCML*

Top 20 expressed transcripts of OLI: *PCDH9*, *PPP2R2B*, NKAIN2, FRMD5, *SPOCK3*, *CTNNA3*, *MBP*, *PDE4B*, *DLG2*, *SLC24A2*, *NTM*, *PLP1*, *RNF220*, *QKI*, *ENSCJAG00000050596*, *DOCK10*, *PLCL1*, *FMNL2*, *DLG1*, and *TMTC2*

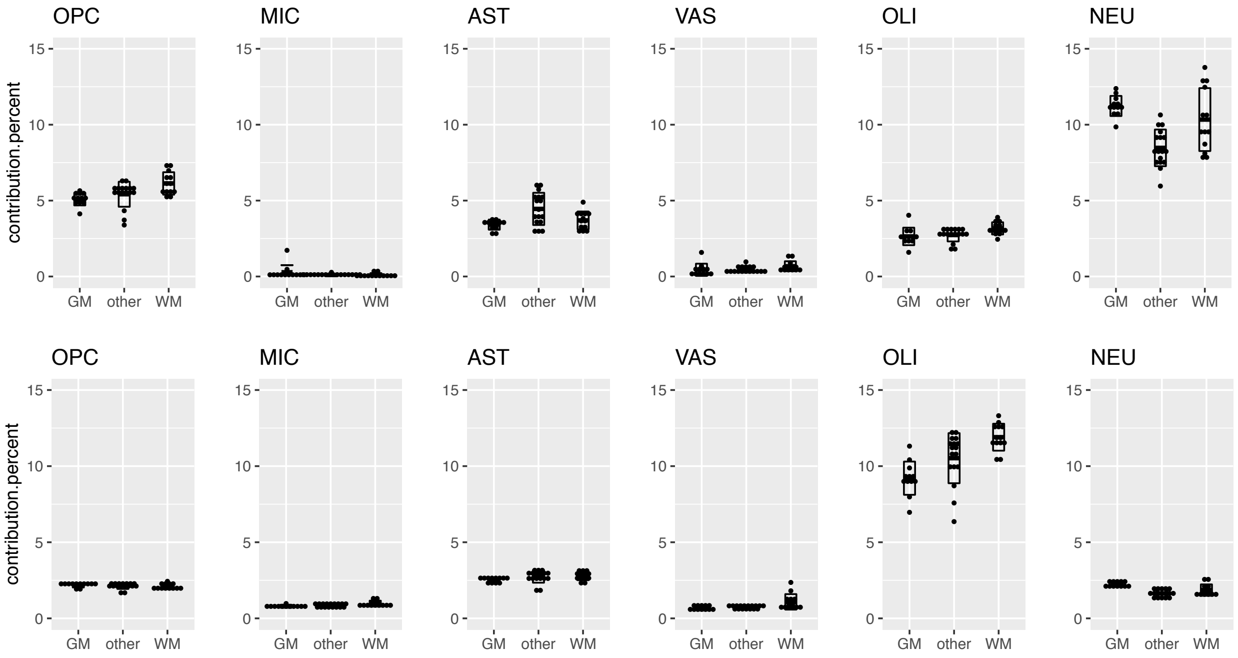

b **Expression pattern of high-ranked, differentially expressed genes for each cell class across 87 subclusters**

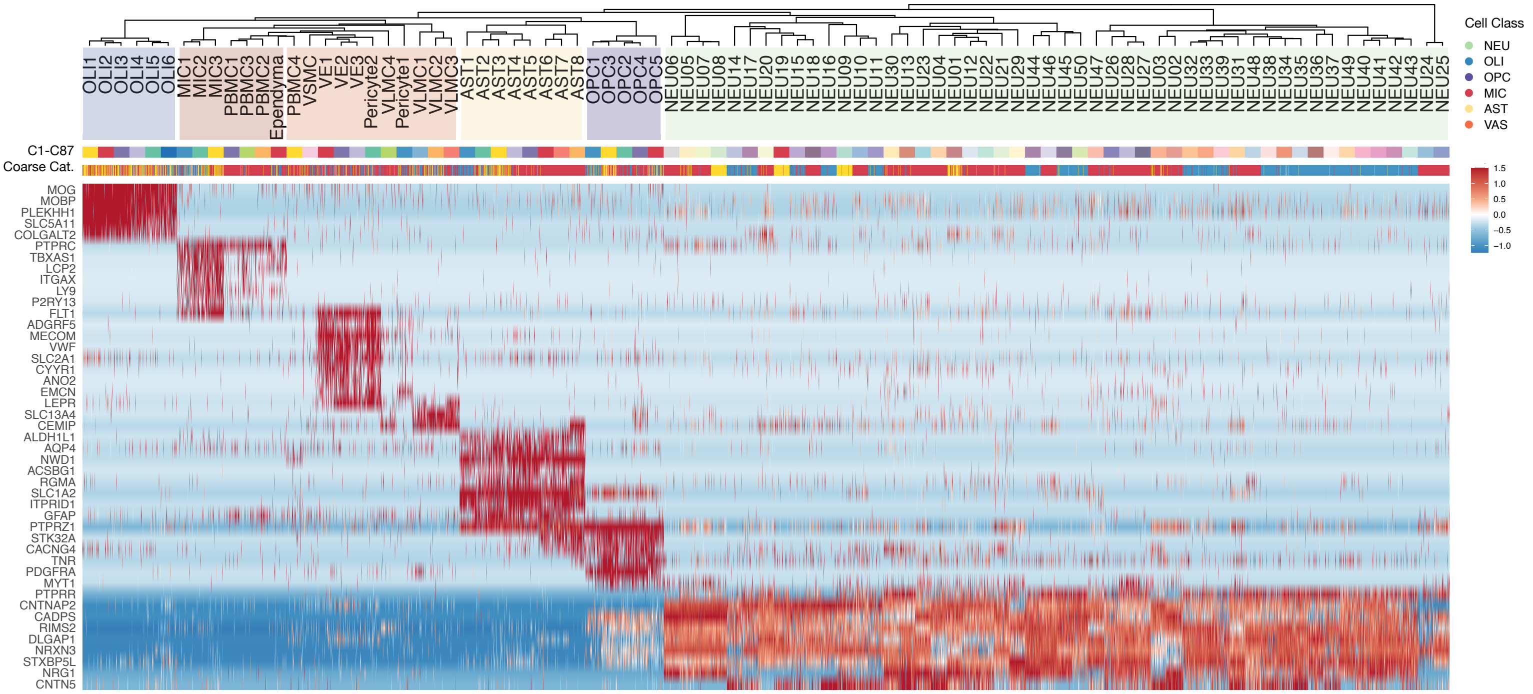

Supplementary Fig. 6. Related to Fig. 1. No systematic cross-contamination was found from top expressed or differentially detected genes from each cell class across subclusters.

a. Box plot showing the cumulative percentage of transcripts contributed by the 20 highest expressed neuronal or oligodendroglial genes in each of the cell classes. Each dot is one sample, grouped by coarse tissue type. There is no systematic tissue type-specific contamination from neuronal or oligodendroglial top-expressed transcripts across cell classes in each tissue-type. n = 42 independent samples; median is annotated. The lower and upper hinges of the box plot correspond to the 25th and 75th percentiles, whiskers extend from the hinges to maxima or minima at most 1.5 times inter-quartile range.

b. Heatmap showing the expression pattern of differentially detected genes of each cell class across subclusters. We found no evidence that subclusters were artifactually impacted through mixing with ambient neuronal transcripts or doublet formation with neurons. If this were the case, it should follow that top-ranked transcripts found in almost every neuron (e.g., *PTPRR*, *CNTNAP2*, *CADP2*, *RIMS2*, *DLGAP1*, *NRXN3*, *STXBP5L*, *NRG1*, *CNTN5*) should be present to a similar degree in glia. Similarly, there was no tissue- specific contribution of ambient transcripts from top-expressed genes found in each cell class. Each of the Level 1 classes was further subclustered (Level 2). Shown is a heatmap and dendrogram of cell types annotated from the Level 2 subclusters, comprising 50 NEU, 6 OLI, 5 OPC, 7 MIC, 8 AST, and 11 VAS subclusters. The heatmap presented here is derived from a pool of 4350 nuclei (50 per cluster, randomly selected; "C50 object"). Each column represents the expression pattern of one nucleus. The first color bar is a color code corresponding to the cluster labels at the endpoint of the dendrogram. The second color bar represents the tissue origin of the nuclei; the 22 sampling sites were grouped into 19 tissue types and further grouped in coarse category: "**WM**" (cerebral white matter), "**GM**" (cortical gray matter), and "**other**." See **Fig. 1d** for the full list. The top 50 differentially enriched genes in each Level 1 cell class were used to calculate Euclidean distances and generate the dendrogram. To aid cluster tracking, the branches of the dendrogram were reordered and colored to show cluster relatedness while retaining the structure of the tree.

SupFig. 7 - Related to Fig1

a **Cross-cluster comparison: averaged gene expression**

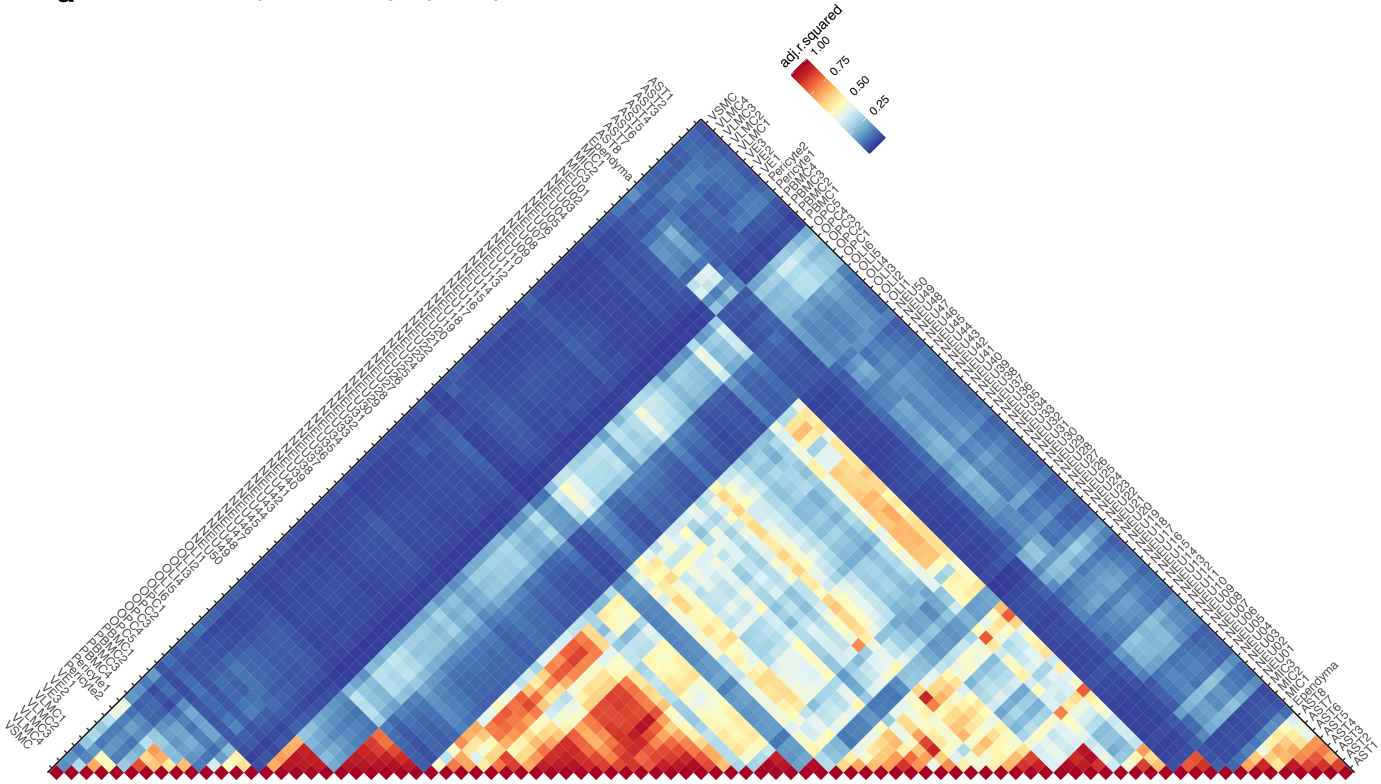

b **Cross-cluster comparison: differentially expressed gene number**

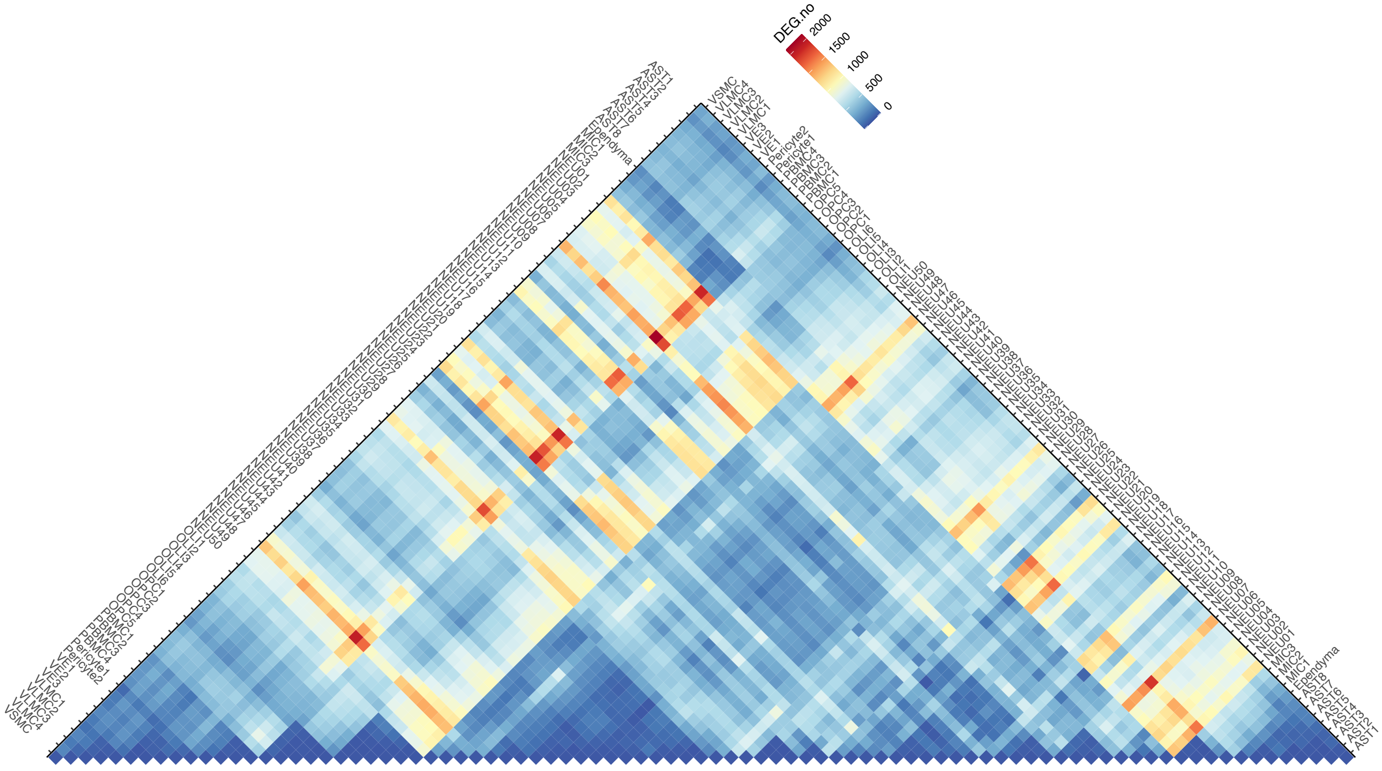

Supplementary Fig. 7. Related to Fig. 1. Assessment of the transcriptomic similarity across each cluster pair.

- **a.** Heatmap showing the paired linear correlation (adjusted r^2) between 87 subclusters derived from the C50 object, which contains 50 cells per subcluster. The expression levels of all genes within each subcluster were normalized and averaged before the comparison.
- **b.** Heatmap showing the number of genes differentially expressed between nuclei in each pair of the 87 subclusters. Both significantly increased and significantly decreased differentially expressed genes are included. The differentially expressed genes were further filtered by their log fold-change (ln(fold-change) > 0.25) and detection frequency (detected in at least 10% of the nuclei).

SupFig. 8 - Related to Fig1

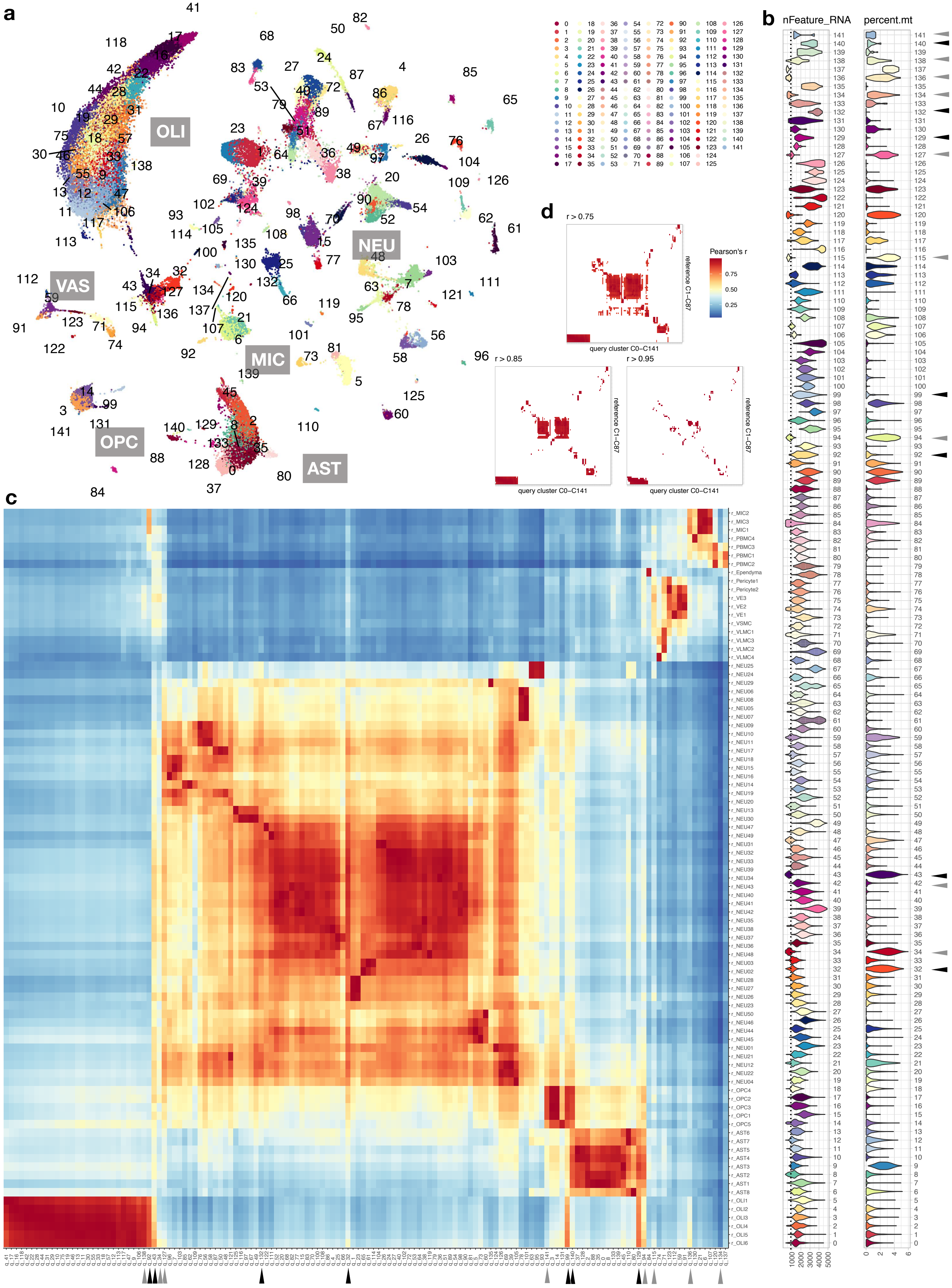

Supplementary Fig. 8. Related to Fig. 1. Unbiased classification of 10% a priori set-aside data yields clusters that recapitulate knowledge-based nuclei classes and subclusters.

- **a.** UMAP scatter-plot visualization of 10% of nuclei (61,803 nuclei) that were randomly set aside from our dataset after initial quality control but prior to further analysis. Analysis parameters were intentionally set to yield a large number of clusters. The top 5000 variable genes were used to calculate 100 principal components and harmonized over IL01_uniqueID labels, then UMAP space and nearest neighbor analyses were calculated on all 100 Harmony embeddings, yielding 142 unbiased clusters.
- **b.** Violin plot showing the percentage of RNA reads that mapped to mitochondria genome (percent.mt) and the number of RNA species (nFeature_RNA) in each cluster. Median is annotated (–).
- **c.** Heatmap showing the Pearson's correlation (r) between the 87 manually annotated subclusters (C50 object, vertical axis) and 142 unbiased clusters (cj10_0 – cj10_141) from the 10% set-aside data (horizontal axis). The expression levels of all genes within each subcluster were normalized and averaged before comparison. Hot spots in the heatmap show correspondence between unbiased and knowledge-derived clusters for all cell classes. Artifact clusters, with "low quality" reads (e.g., low nFeature_RNA or high percent.mt, gray arrowheads) or nuclei doublets (high correlation with multiple cell classes, black arrowheads) are easily spotted on the heatmap.
- **d.** Filtered heatmaps showing the distribution of the paired correlation of Pearson's r over 0.75, 0.85, and 0.95 thresholds. At 0.75 threshold, all 87 annotated clusters correspond to one or several clusters from the set-aside data. At 0.95 cutoff, subclusters of neurons can be distinguished.

SupFig. 9 - Related to Fig2

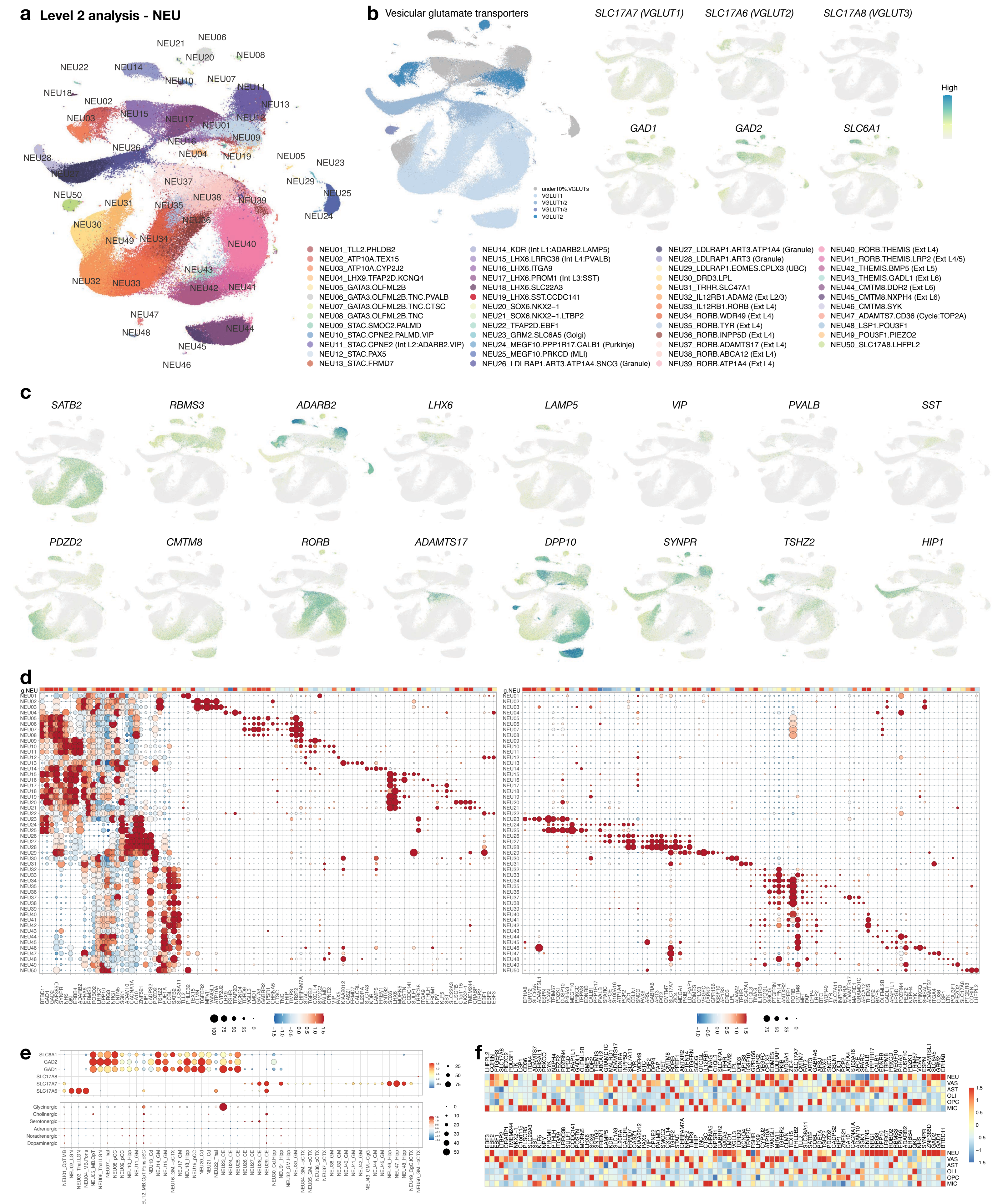

Supplementary Fig. 9. Related to Fig. 2. Although neuron subclusters in different cerebral cortices are mostly shared, there are subclusters unique to occipital cortex, lateral geniculate nucleus, thalamus, caudate, hippocampus, cerebellum, and posterior corpus callosum.

- **a.** UMAP scatter-plot visualization of neuron (NEU) nuclei colored by subcluster. Enriched markers selected from dot plot (Supplementary Fig. 9d) and reference indexed cell types are annotated. **L1 – L6**, layer 1 – layer 6; **Int**, inhibitory neurons; **Ext**, excitatory neurons; **MLI**, molecular layer interneurons; **UBC**, unipolar brush cell.
- **b.** Left, UMAP scatter-plot visualization of NEU nuclei colored by their primary VGLUT gene expression. Right, UMAP scatter-plot visualization of NEU nuclei colored by the expression of glutamatergic (*SLC17A6*, *SLC17A7*, *SLC17A8*) and GABAergic (*GAD1*, *GAD2*, *SLC6A1*) genes. See Supplementary Fig. 9e for detail.
- **c.** UMAP scatter-plot visualization of NEU nuclei colored by the expression of genes specific to small subsets of neuron subclusters.
- **d.** Dot plot showing mean-centered and z-score-scaled marker gene expression for NEU subclusters. Dot size indicates the percentage of nuclei in the subcluster in which each gene was detected. Scaling is relative to expression across all NEU nuclei in which a given gene was detected.
- **e.** Dot plots showing mean-centered and z-score-scaled expression of marker genes related to neurotransmission across NEU subclusters. Top, filtered dot plot; for clarity, the glutamatergic (*SLC17A6*, *SLC17A7*, *SLC17A8*) and GABAergic (*GAD1*, *GAD2*, *SLC6A1*) gene expression values were dropped if detected in <10% of nuclei in a given cluster (related to **Supplementary Fig. 9b**). Bottom, unfiltered dot plot: genes related to each neurotransmitter type — dopaminergic (*TH*, *DDC*, *SLC6A3*), noradrenergic (*TH*, *DCC*, *DBH*, *SLC6A2*), adrenergic (*TH*, *DDC*, *DBH*, *PNMT*), serotonergic (*TPH2*, *DDC*, *SLC6A4*), cholinergic (*CHAT*, *SLC5A7*), and glycinergic (*SLC6A9*, *SLC6A5*) — were aggregated.
- **f.** Heatmap showing mean-centered and z-score-scaled NEU marker gene expression for all Level 1 classes (C50 object, 50 cells per subcluster).

SupFig. 10 - Related to Fig2

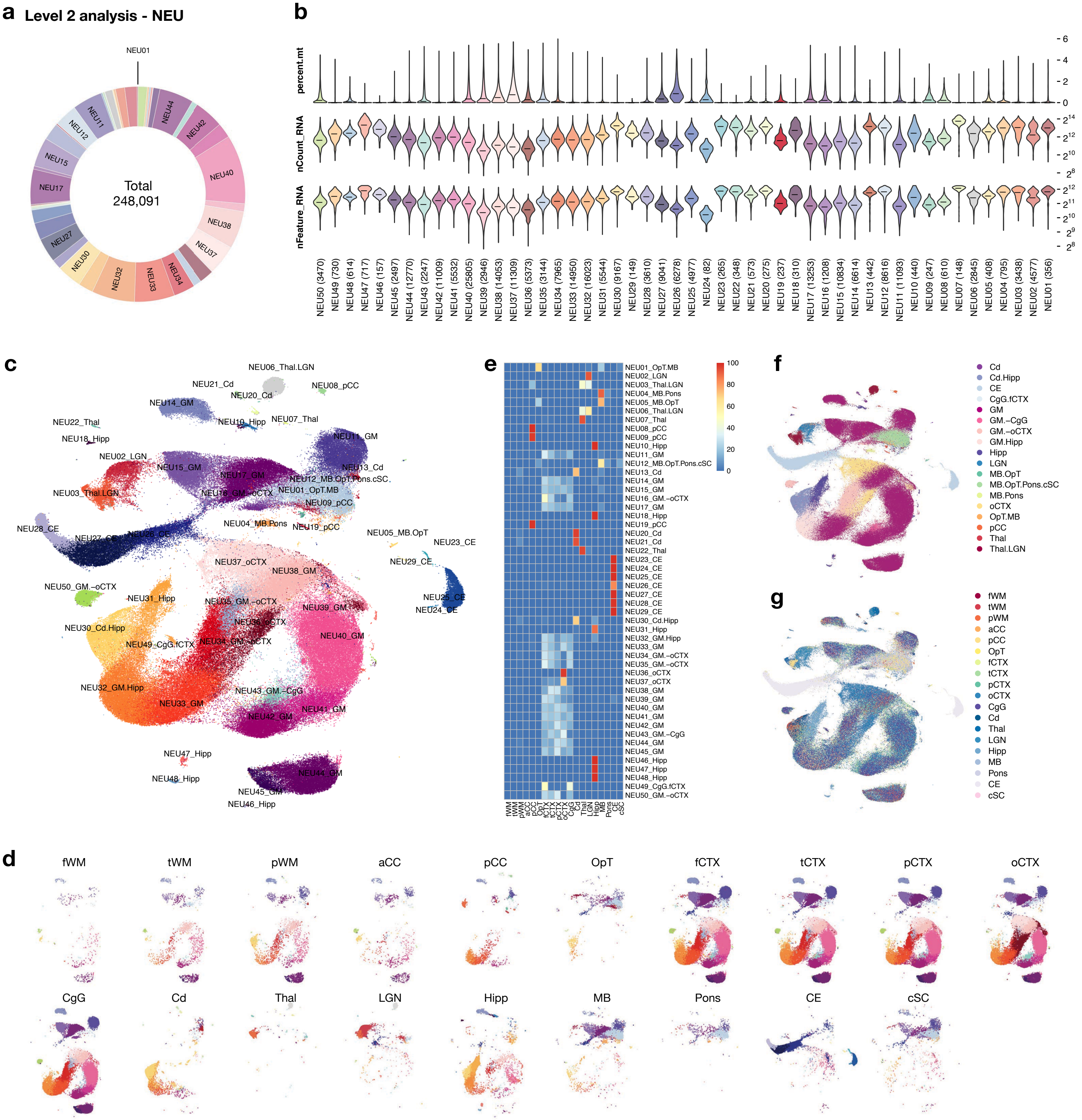

Supplementary Fig. 10. Related to Fig. 2. Nuclei features in neurons.

- **a.** Donut plot showing the number of NEU nuclei that passed Level 2 quality control, annotated by subcluster.
- **b.** Violin plot showing the percentage of RNA reads that mapped to mitochondria genome (percent.mt), the number of RNA detected (nCount_RNA), and the number of RNA species (nFeature_RNA) in each cluster. Median is annotated (–).
- **c.** UMAP scatter-plot visualization of NEU nuclei colored by subcluster.
- **d.** UMAP scatter-plot visualization of NEU nuclei colored by subcluster and split by sampling site.
- **e.** Heatmap showing the percentage of nuclei in each NEU subcluster drawn from different sampling sites; 100% per row (subcluster).
- **f.** UMAP scatter-plot visualization of NEU nuclei colored by the similarity of tissue type based on the heatmap showed in **Supplementary Fig. 10e**, regrouped by combining similar tissue types.
- **g.** UMAP scatter-plot visualization of NEU nuclei colored by tissue type.

SupFig. 11 - Related to Fig2

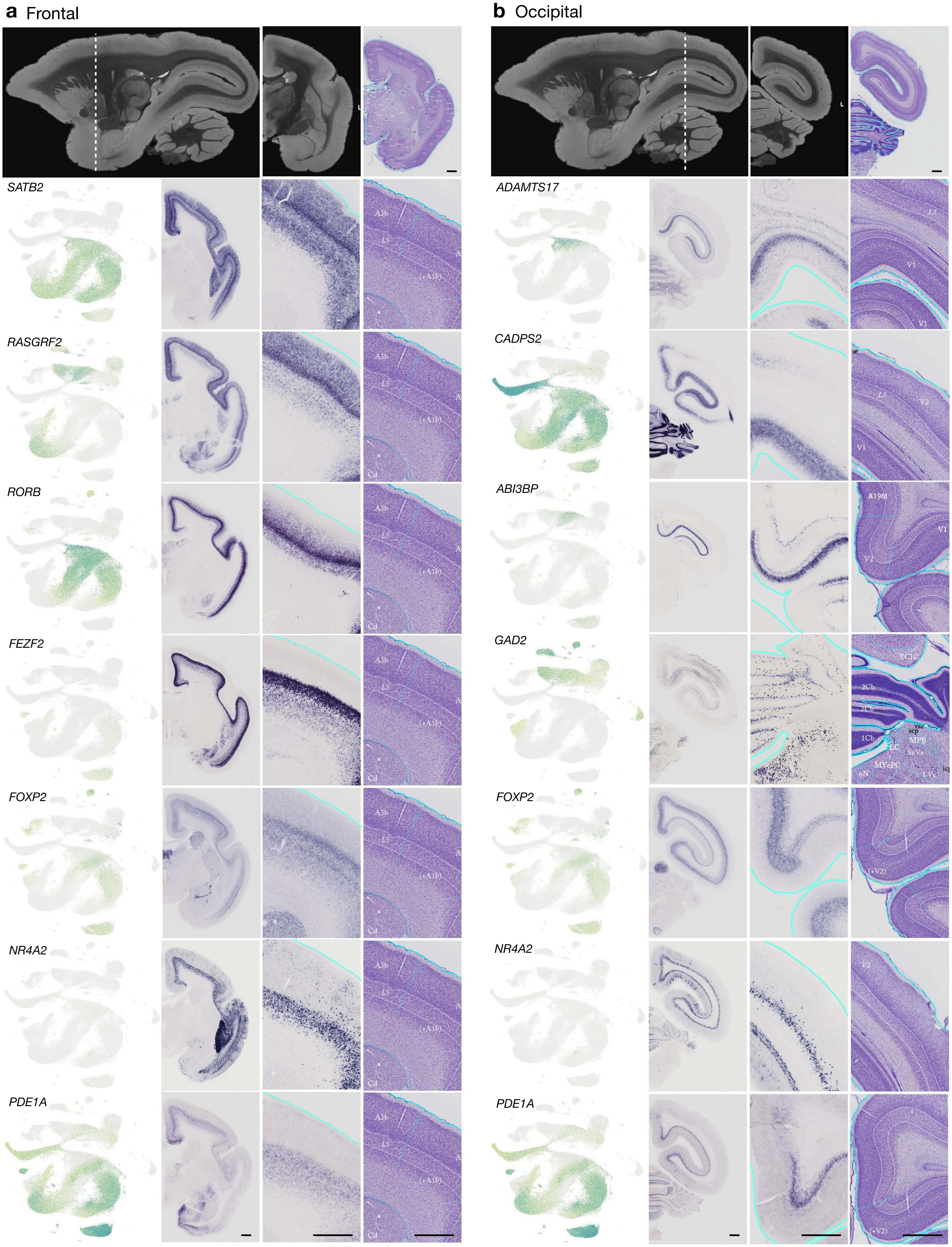

Supplementary Fig. 11. Related to Fig. 2. Cortical excitatory neurons are arranged into a continuous UMAP projection corresponding to lamination.

a – **b**. Approximate cross-sectional plane (white line; **a**, frontal; **b**, occipital) indicated on a sagittal view of an adult marmoset postmortem T2*-weighted MRI (row 1, left). Coronal view of the same adult marmoset T2*-weighted MRI (row 1, middle). Coronal histological section of a P0 marmoset stained with Nissl's method (right) and anatomically matched to the adult coronal T2*-weighted MRI slice (row 1, right). Rows 2–7: UMAP scatter-plot visualization of neurons colored by selected marker gene expression (column 1). Selected in situ hybridization (ISH) of genes enriched in all or a subset of cortical layers (column 2). Enlarged area of the ISH image was anatomically matched to a Nissl-stained section for regional reference (columns 3 and 4). Scale bar, 1mm. MRI scans are derived from the Marmoset Brain Mapping database (https:// marmosetbrainmapping.org/)[1,2]. Histological Nissl stain and ISH images are from the Marmoset Gene Atlas (https://gene-atlas.brainminds.riken.jp/)[3,4].

SupFig. 12 - Related to Fig2

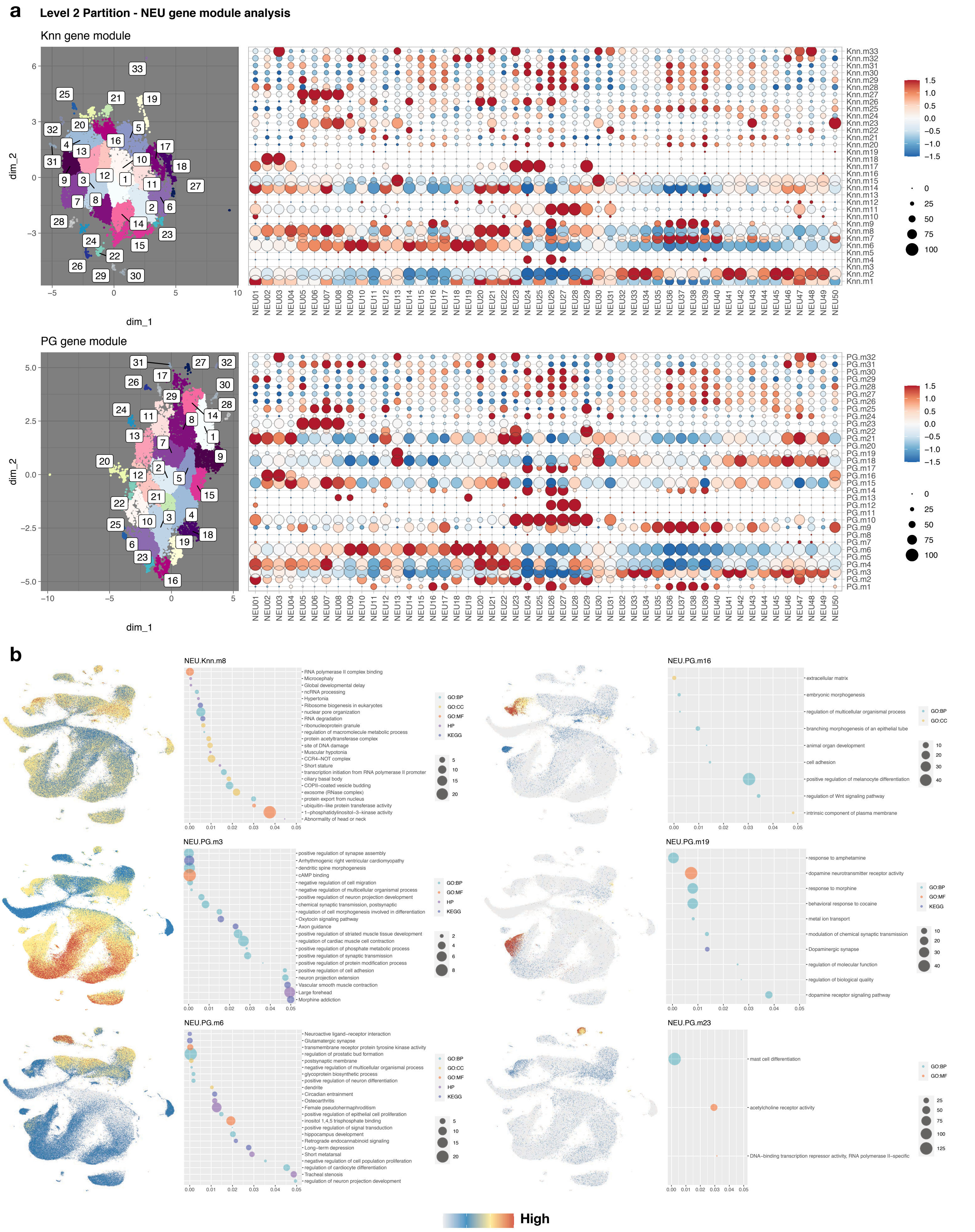

Supplementary Fig. 12. Related to Fig. 2. Gene module analysis in neurons.

a. UMAP scatter plot visualization of genes grouped by expression similarity across cells. Genes that passed Moran's *I* statistic spatial test (< 5% FDR) over *k*-nearest neighbor graph (Knn, k=25, left), or over trajectory-learned principal graph (PG, right), by Monocle3 graph_test function. Genes are grouped and colored by modules identified in each graph test by find_gene_modules function with resolution of 0.001. The list of genes in each module were aggregated through Seurat v3 AddModuleScore function. Dot plot showing the averaged and scaled expression of each module gene set across NEU subclusters.

b. UMAP scatter-plot visualization of nuclei colored by averaged expression of selected modules enriched in a subset of neurons (see Supplementary Data 2 for full list). Dot plot showing the enriched GO terms from the list of genes in each module. GO, gene ontology; BP, biological process; CC, cellular component; MF, molecular function; HP, human phenotype ontology; KEGG, Kyoto Encyclopedia of Genes and Genomes.

SupFig. 13 - Related to Fig2

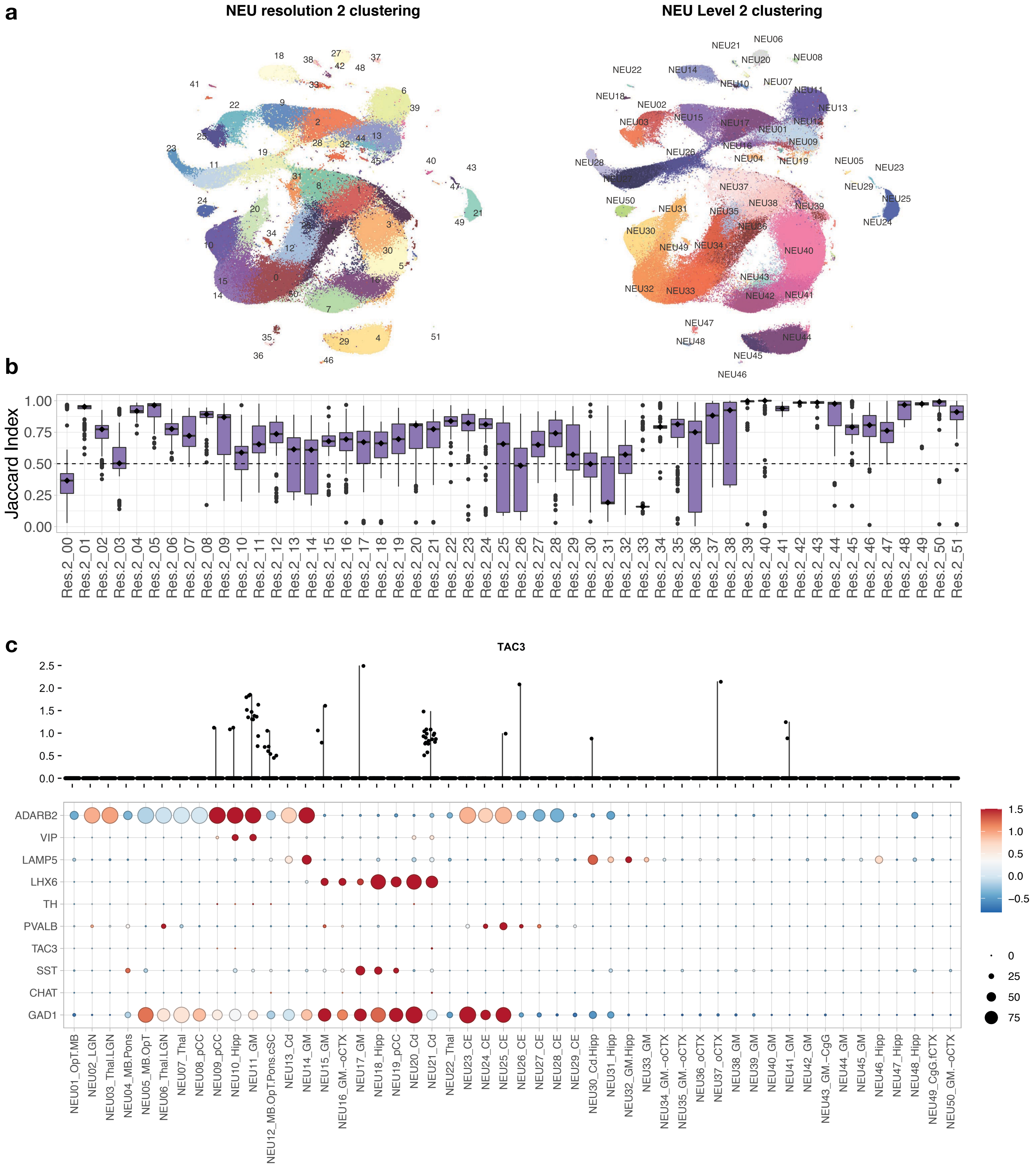

Supplementary Fig. 13. Related to Fig. 2. Neuron clustering stability test and the presence of TAC3^{+} inhibitory neurons.

- **a.** Left, UMAP scatter plot visualization of nuclei colored by clustering at resolution 2 (see Methods). Right, UMAP scatter plot visualization of nuclei colored by Level 2 clustering.
- **b.** Box-and-whisker plot showing the Jaccard index as a measure of clustering stability (see Methods). Total nuclei in the NEU partition were sampled at 90% repeatedly and reclustered over 100 iterations at resolution of 2. n = 100 of independent iterations, median is annotated (black diamond shape). The lower and upper hinges of the box plot correspond to the 25th and 75th percentiles, whiskers extend from the hinges to maxima or minima at most 1.5 times inter-quartile range.
- **c.** Bottom, dot plots showing mean-centered and z-score-scaled expression of marker genes across NEU subclusters. Top, dot plot showing the expression of a primate-specific gene (*TAC3*)[5].

SupFig 14 - Related to Fig3

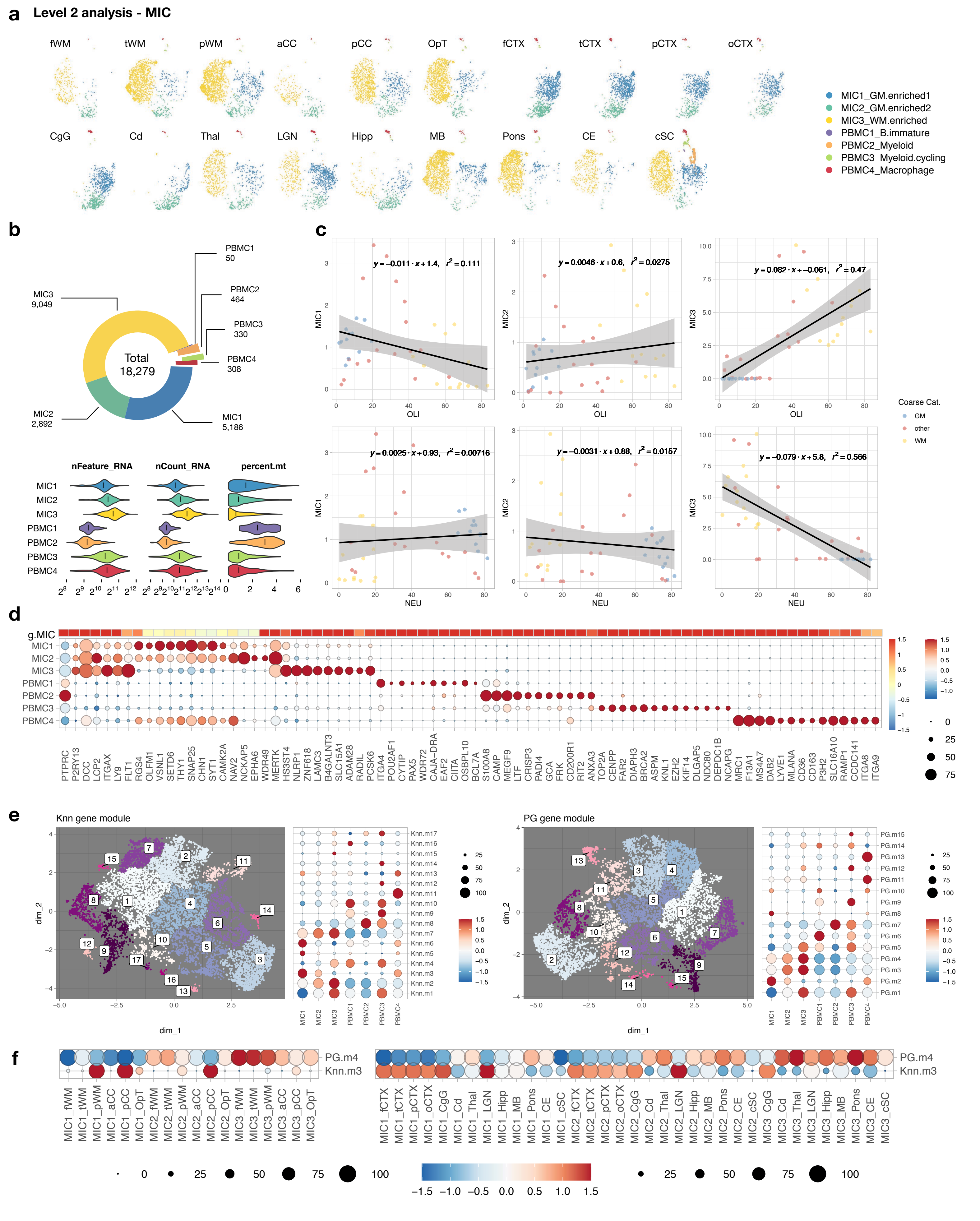

Supplementary Fig. 14. Related to Fig. 3. Nuclei features and gene module analysis in microglia/myeloid lineage cells (MIC).

- **a.** UMAP scatter plot visualization of microglia/immune cells (MIC) colored by subcluster and split by sampling site.
- **b.** Top, donut plot showing the number of nuclei that passed Level 2 quality control, annotated by subcluster. Bottom, violin plot showing the percentage of RNA reads that mapped to mitochondria genome (percent.mt), the number of RNA detected (nCount_RNA), and the number of RNA species (nFeature_RNA) in each cluster. Median is annotated (–).
- **c.** Linear regression on the normalized abundance of MIC1, MIC2, and MIC3 subclusters with oligodendrocyte (OLI) or neuron (NEU) clusters. The abundance of WM-microglia (MIC3) corelates with the abundance of OLI and NEU. The density of GM-microglia (MIC1 and MIC2) is independent of tissue type. Data are derived from the C50 object (50 cells per subcluster).
- **d.** Heatmap and dot plot showing mean-centered and z-score-scaled marker gene expression in global space (heatmap, MIC average expression among Level 1 classes) and local space (dot plot, among Level 2 subclusters). The portion of the heatmap that represents global microglia/immune cells (g.MIC) is reproduced here from the full heatmap shown in **Supplementary Fig. 15b**.
- **e.** UMAP scatter-plot visualization of genes grouped by expression similarity across cells. Genes that passed Moran's *I* statistic spatial test (< 5% FDR) over *k*-nearest neighbor graph (Knn, k=25, left), or over trajectory learned principal graph (PG, right), were identified by Monocle3 graph_test function. Genes are grouped and colored by modules identified in each graph test by find_gene_modules function with resolution of 0.001. The list of genes of a given module was aggregated through Seurat v3 AddModuleScore function. Dot plot showing the averaged and scaled expression of each module gene set across MIC subclusters.
- **f.** Dot plot showing the averaged and scaled expression of PG.m4 (WM-microglia enriched) and Knn.m3 (GM-microglia enriched) modules across MIC subclusters in each tissue type. Compared to deep GM, the module involved in neuronal activity (Knn.m3) is highly enriched in MIC1 and MIC2 of cortical areas and LGN. In WM, the module involved in stimulus response (PG.m4) is mostly enriched in fWM, tWM, and pWM. Although there appears to be high expression of Knn.m3 in CgG for MIC3, this is due to low abundance of MIC3 (only 4 nuclei) in CgG. Taken together, these results further emphasize the presence of regional transcriptomic diversity.

SupFig 15 - Related to Fig3

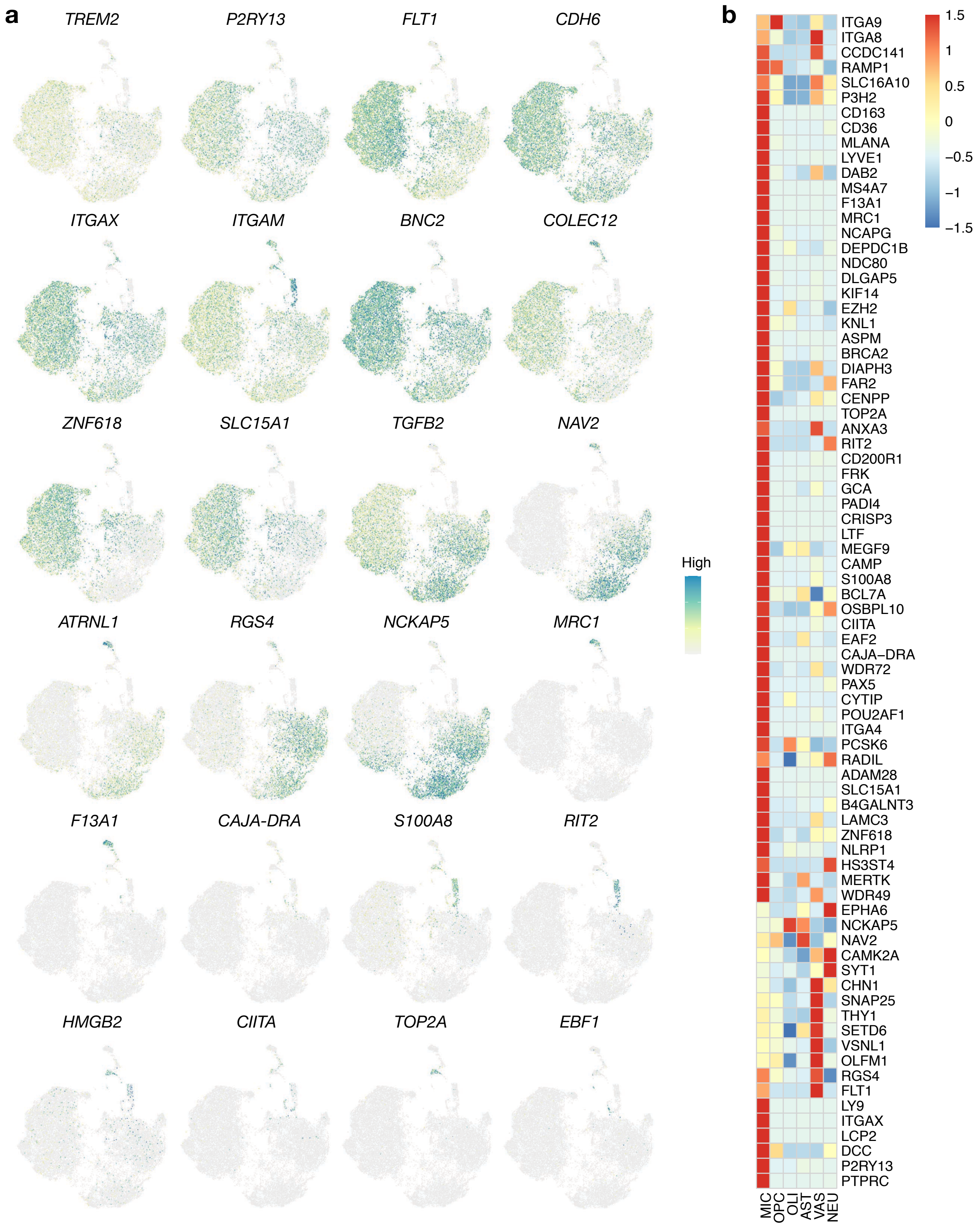

Supplementary Fig. 15. Related to Fig. 3. Selected gene expression in microglia/myeloid lineage cells (MIC) and compared across 6 cell classes.

- **a.** UMAP scatter plot visualization of MIC nuclei colored by selected marker gene expression.
- **b.** Heatmap showing averaged and z-score-scaled expression of MIC genes in other Level 1 clusters. Data are derived from the C50 object (50 cells per subcluster).

SupFig 16 - Related to Fig3

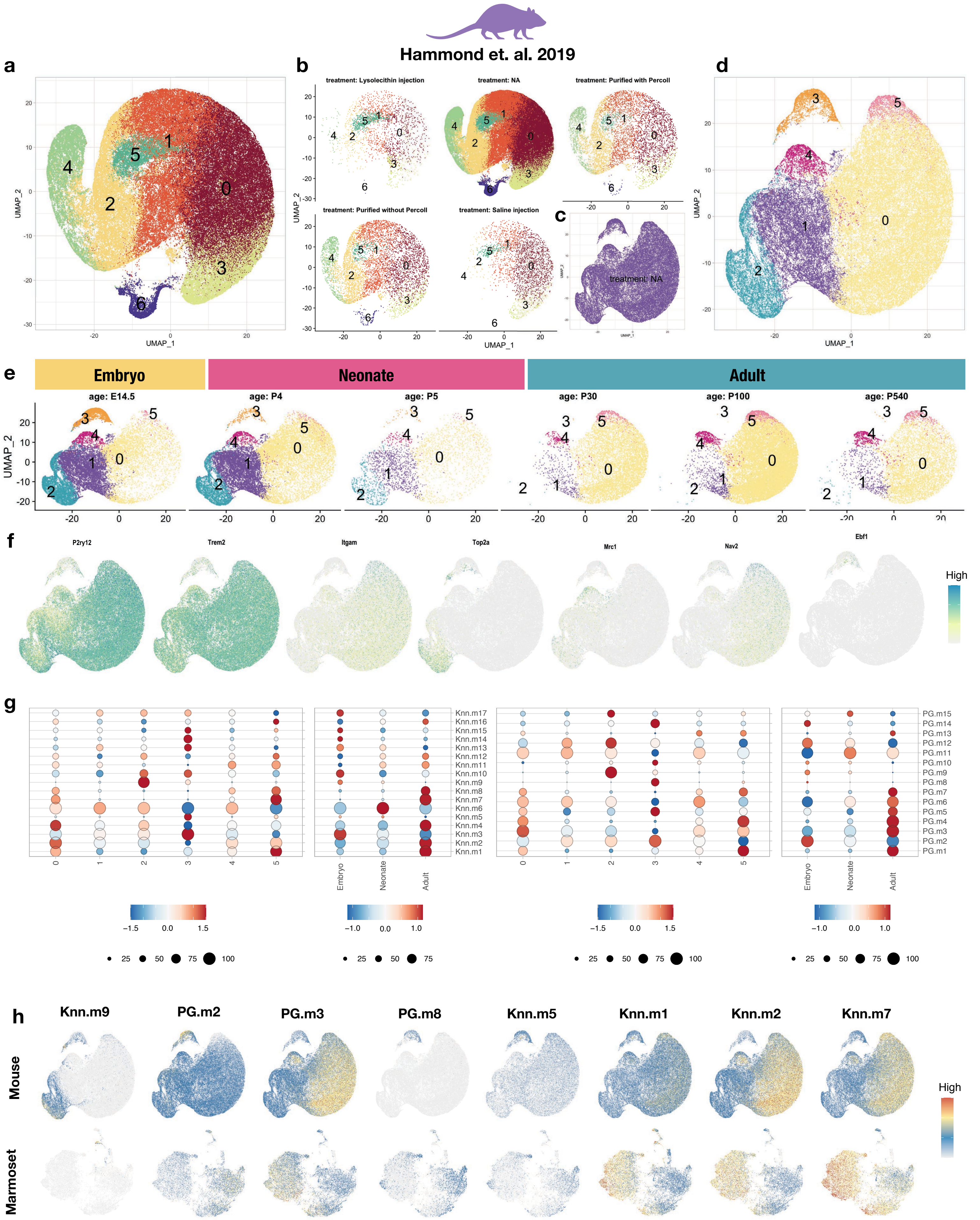

Supplementary Fig. 16. Related to Fig. 3. The expression of marmoset GM-microglia gene modules is also elevated in microglia of young mice, whereas the expression of marmoset WM-microglia gene modules is elevated in microglia of adult mice.

- **a.** Previously published and deposited data were reanalyzed and reclustered with a pipeline similar to that used in the current study. UMAP scatter-plot visualization of mouse microglia colored by subclusters identified by our pipeline.
- **b.** UMAP scatter-plot visualization of mouse microglia colored by subclusters identified by our pipeline and split by treatment condition defined in the original publication.
- **c.** Mouse homeostatic microglia (collected from untreated animals) were selected, reanalyzed, and reclustered with our pipeline.
- **d.** UMAP scatter-plot visualization of mouse homeostatic microglia colored by subclusters identified by our pipeline.
- **e.** UMAP scatter-plot visualization of mouse homeostatic microglia colored by subclusters identified by our pipeline and split by the age of the cell as defined in the original publication.
- **f.** UMAP scatter-plot visualization of mouse microglia colored by selected marker-gene expression.
- **g.** Dot plot showing the averaged and scaled expression of each module's gene set defined in marmoset microglia across subclusters (first and third panels) and age (second and fourth panels) of mouse homeostatic microglia.
- **h.** UMAP scatter plot visualization of mouse microglia (top) and marmoset MIC nuclei (bottom) colored by averaged expression of selected modules found in marmoset.

SupFig 17 - Related to Fig3

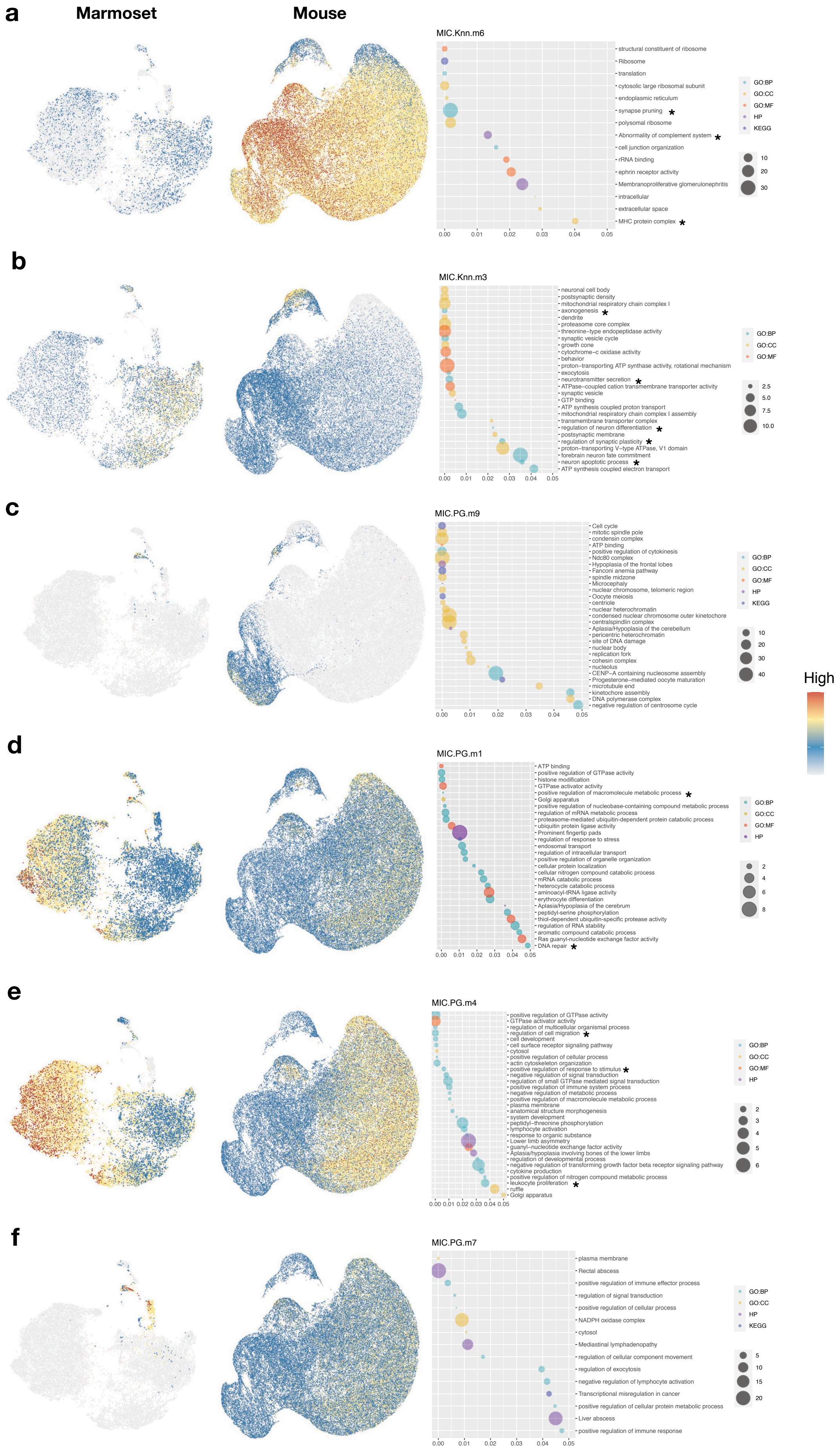

Supplementary Fig. 17. Related to Fig. 3. The expression of gene modules defined in marmoset immune cells in both marmoset and mouse immune cells.

a – f. UMAP scatter plot visualization of marmoset (left) and mouse microglia (middle) colored by aggregated expression of genes in each module. Right, dot plot showing the enriched GO terms from the list of genes in each module. GO, gene ontology; BP, biological process; CC, cellular component; MF, molecular function; HP, human phenotype ontology; KEGG, Kyoto Encyclopedia of Genes and Genomes. *Terms mentioned in the results section.

See also Supplementary Data 2 and 4.

SupFig 18 - Related to Fig4

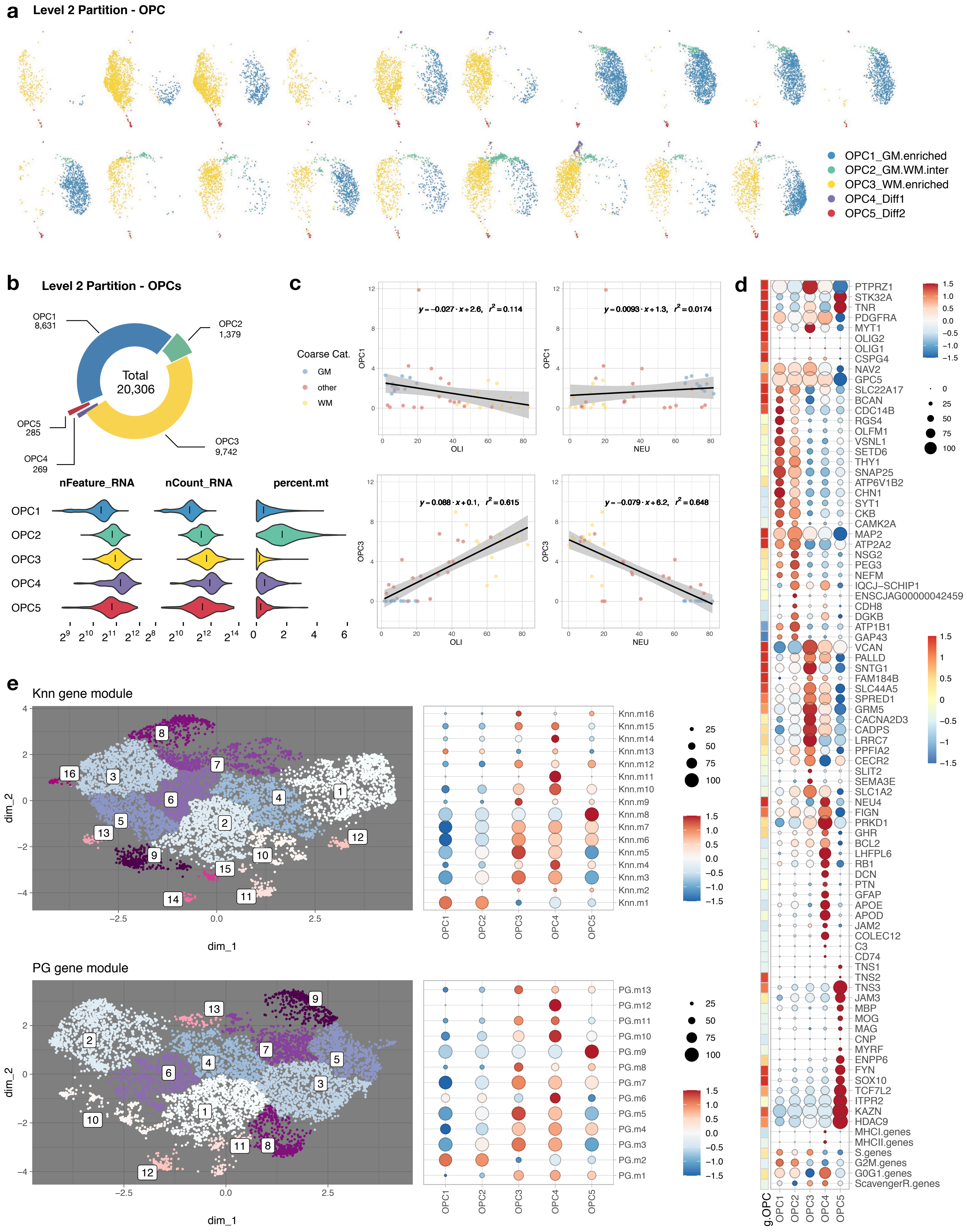

Supplementary Fig. 18. Related to Fig. 4. Oligodendrocyte progenitor cell (OPC) subclusters are spatially segregated.

- **a.** UMAP scatter-plot visualization of oligodendrocyte precursor cells (OPC) colored by Level 2 subclustering and split by sampling site.
- **b.** Top, donut plot showing the number of nuclei that passed the level 2 quality control, annotated by subcluster. Bottom, violin plot showing the percentage of RNA reads that mapped to mitochondria genome (percent.mt), the number of RNA molecules detected (nCount_RNA), and the number of RNA species (nFeature_RNA) in each cluster. Median is annotated (–).
- **c.** Linear regression on the normalized abundance of Level 2 subclusters (OPC1 and OPC3) with OLI or NEU partition annotated in Level 1 analysis. Individual samples are colored by coarse category. The abundance of WM-enriched OPC (OPC3) corelates with the abundance of OLI and NEU. The abundance of GM-enriched OPC (OPC1) is proportional to total nuclei number per sample across different tissue types.
- **d.** Heatmap and dot plot showing marker gene expression in global space (heatmap, OPC average expression among Level 1 classes) and local space (dot plot, Level 2 subclusters). See Supplementary Data 4 for gene set list. The portion of the heatmap that represents global OPC (g.OPC) is reproduced here from the full heatmap shown in Supplementary Fig. 20b.
- **e.** UMAP scatter-plot visualization of genes grouped by expression similarity across cells. Genes that passed Moran's *I* statistic spatial test (< 5% FDR) over the *k*-nearest neighbor graph (Knn, k=25, left), or over the trajectory learned principal graph (PG, right), were identified by Monocle3 graph_test function. Genes are grouped and colored by modules that were identified in each graph test by find_gene_modules function with resolution of 0.001. The list of genes of each module was aggregated through Seurat v3 AddModuleScore function. Dot plot showing the averaged and scaled expression of each module gene set across OPC subclusters.

SupFig 19 - Related to Fig4

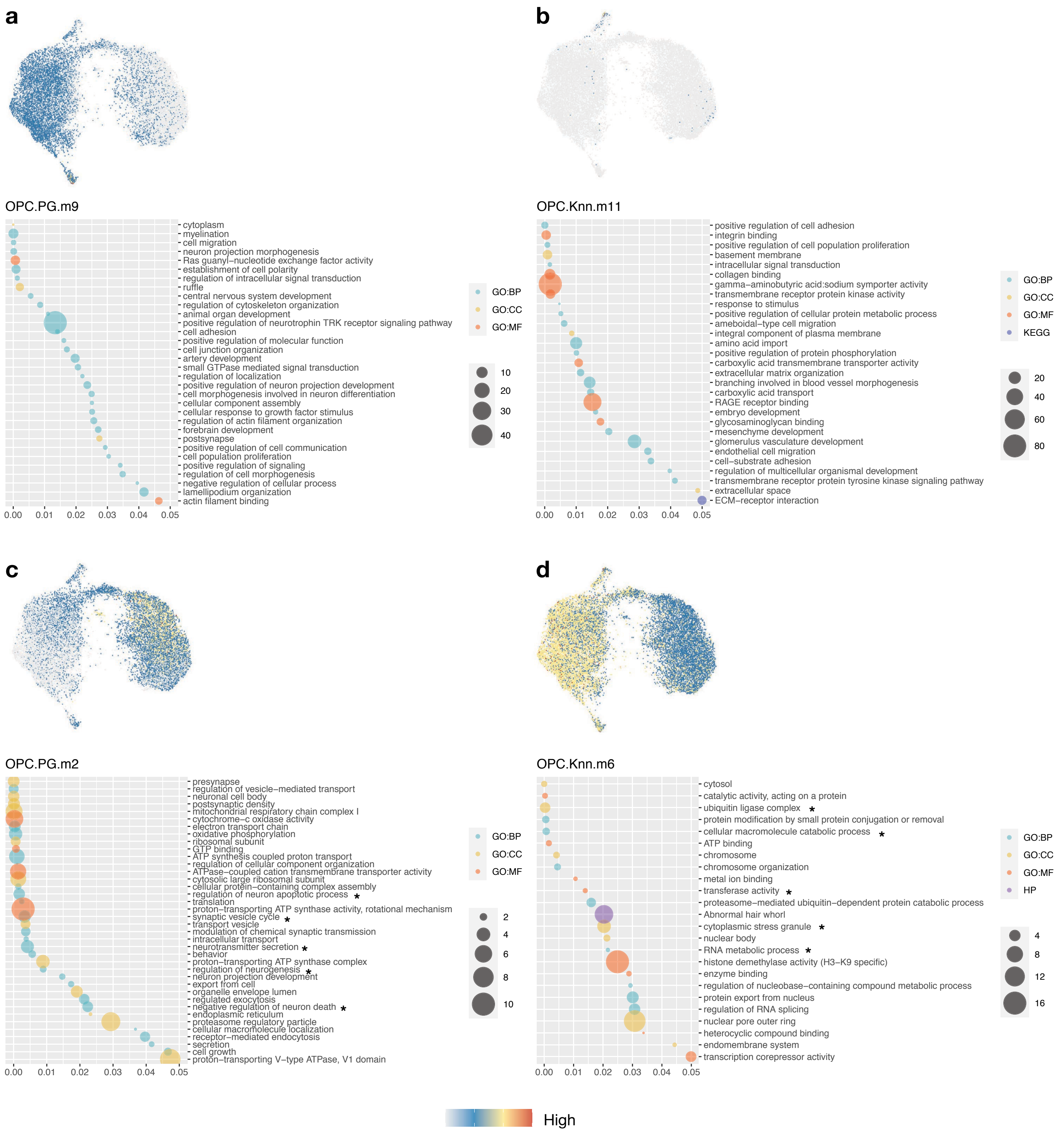

Supplementary Fig. 19. Related to Fig. 4. GM-OPC are enriched with pathways related to neuronal support, and WM-OPC are enriched with pathways related to component organization, molecule modification, and cell motility.

a – d. Top, UMAP scatter plot visualization of marmoset OPC colored by aggregated expression of gene in each module. Bottom, dot plot showing the enriched GO terms from the list of genes in each module. GO, gene ontology; BP, biological process; CC, cellular component; MF, molecular function; HP, human phenotype ontology; KEGG, Kyoto Encyclopedia of Genes and Genomes. *Terms mentioned in the results section.

SupFig 20 - Related to Fig4

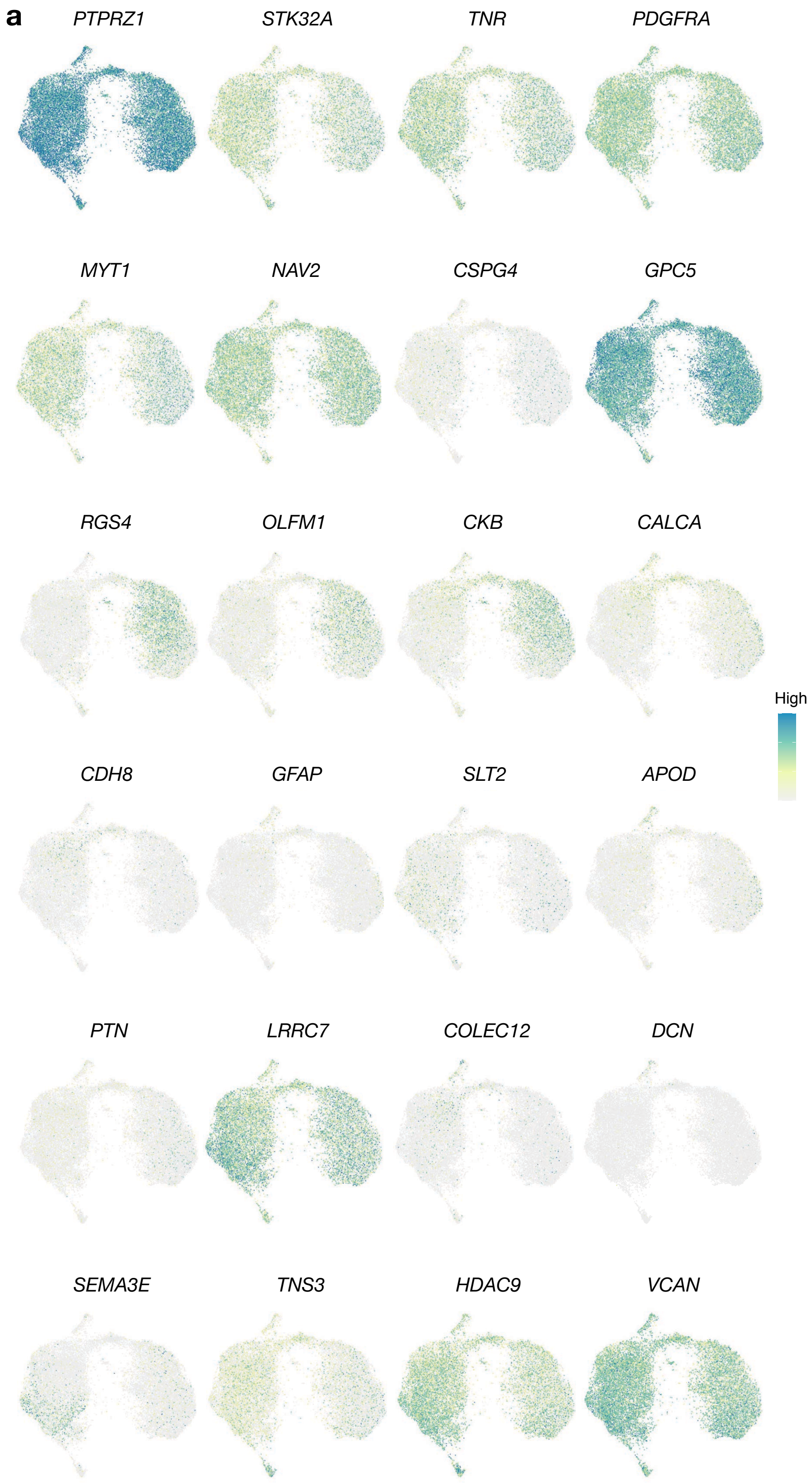

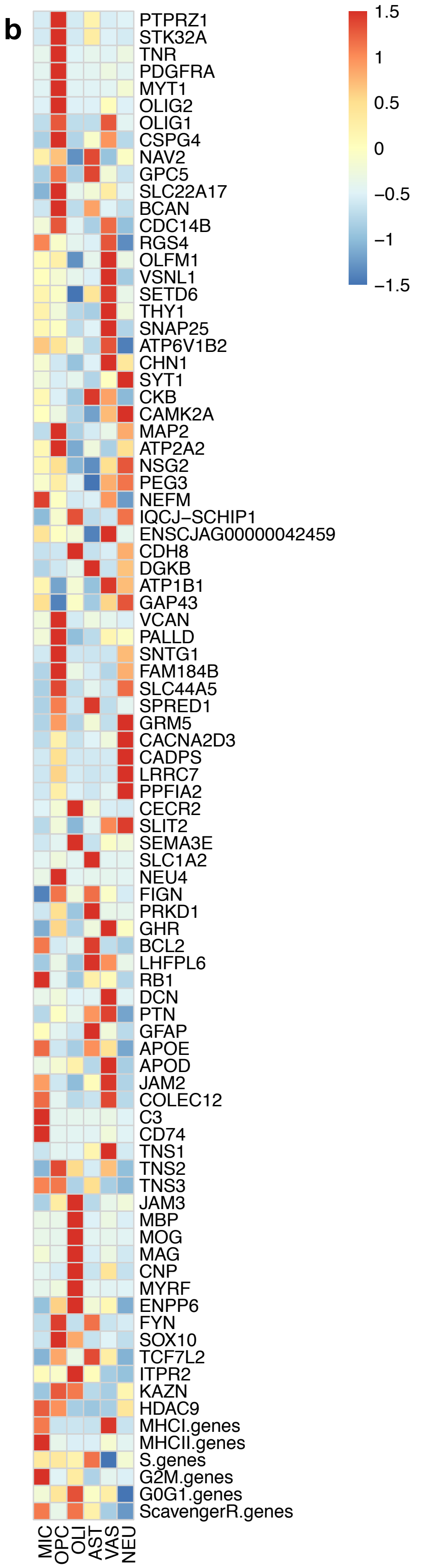

Supplementary Fig. 20. Related to Fig. 4. Selected gene expression in OPC and compared across 6 cell classes.

- **a.** UMAP scatter-plot visualization of OPC nuclei colored by selected marker-gene expression.
- **b.** Heatmap showing the averaged and z-score scaled marker of OPC genes in other Level 1 clusters. The same object (C50 object) was used to make plots shown in Supplementary Fig. 18d.

SupFig 21 - Related to Fig4

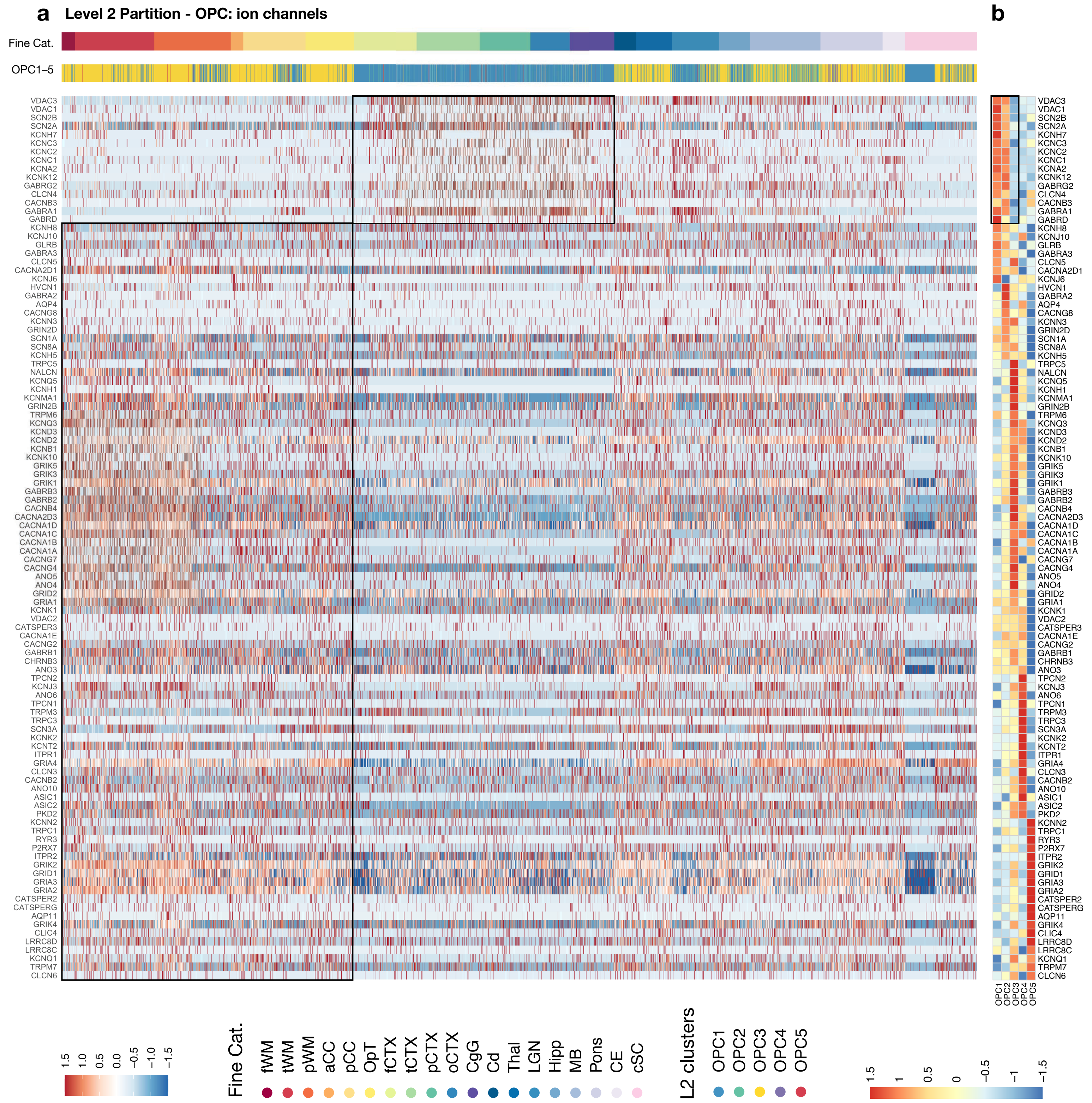

Supplementary Fig. 21. Related to Fig. 4. Oligodendrocyte progenitor cells (OPC) exhibit different ion channel profiles across tissue types.

a. Heatmap showing the z-score-scaled gene expression in OPC, with each vertical line representing the expression pattern of one nucleus. The first color bar represents the tissue origin of the OPC, and the second color bar the OPC subcluster annotation. The black boxes highlight two sets of ion channels with markedly different expression levels in white matter (higher for genes in the box on the lower left) and gray matter (higher in the box on the upper right).

b. Heatmap showing the averaged and z-score-scaled expression in OPC subclusters of the same genes shown.

SupFig 22 - Related to Fig4

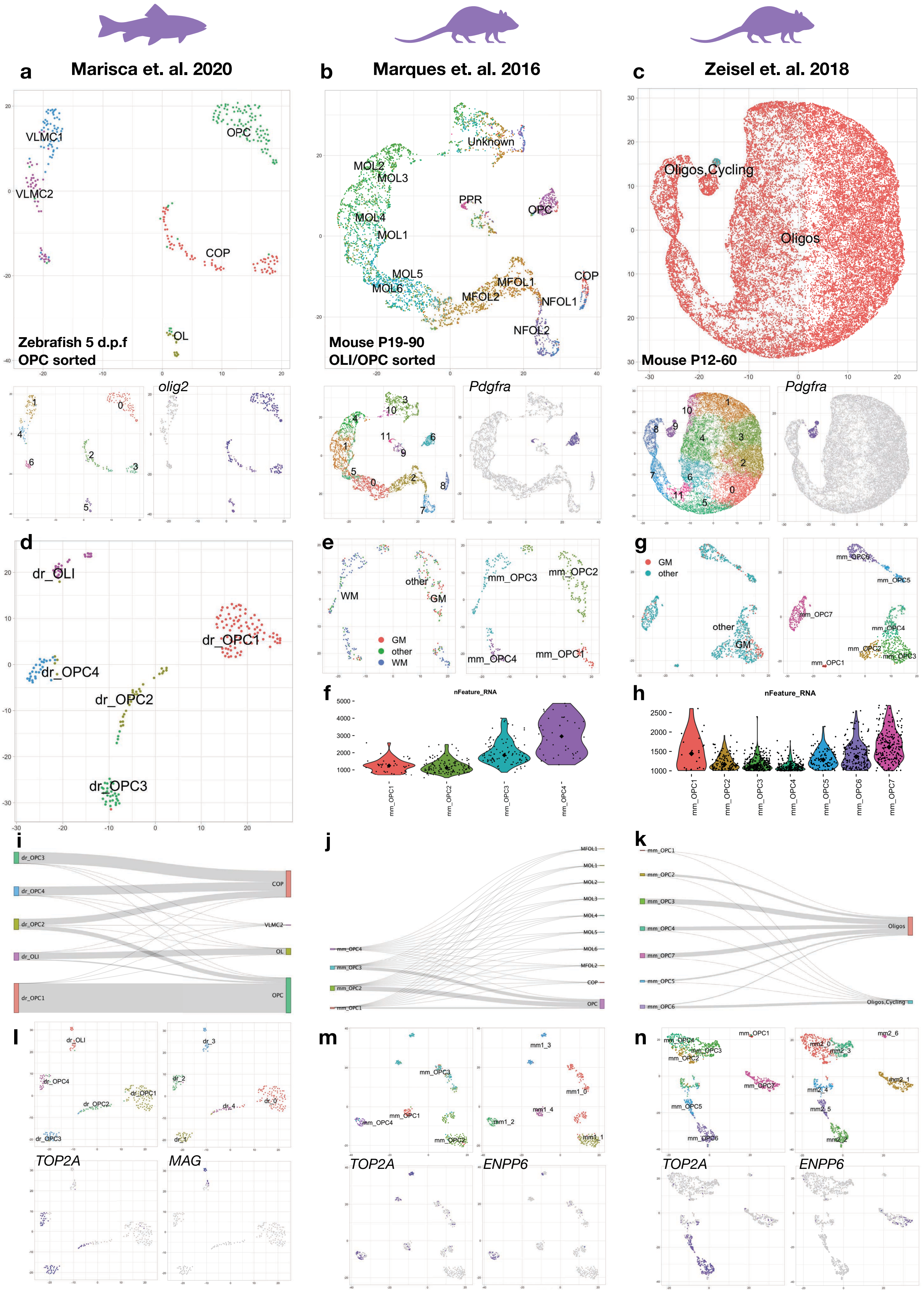

Supplementary Fig. 22. Related to Fig. 4. Reanalysis of zebrafish and mouse oligodendrocyte progenitor cells (OPC).

a – **c**. Data deposited from published studies in zebrafish sorted OPC (**a**), and mouse oligodendrocyte lineage cells (**b** and **c**), were reanalyzed and reclustered with a pipeline similar to that used in the marmoset study. The original annotation defined by the authors is overlaid onto the UMAP scatter plots in the top row. In the bottom row, for each dataset, the left panel shows our new cluster annotations. The right panel shows the OPC clusters further analyzed in subsequent panels of this figure, specifically clusters 0, 2, 3, 5 (zebrafish; *Olig2*$^{+}$), 5 (mouse, Marques et. al. 2016; *Pdgfra*$^{+}$), and 9 (mouse, Zeisel et. al. 2018; *Pdgfra*$^{+}$).

d. New UMAP scatter plot showing reclustering of the selected zebrafish OPCs and oligodendrocytes (Clusters 0, 2, 3, 5 in lower-right panel of A) with new labels.

e. New UMAP scatter plot showing reclustering of the selected mouse OPC clusters (Cluster 5 in lower-right panel of B) colored by new subclusters (right). Using information about the tissue origin provided by the original authors, subclusters were colored and annotated with new labels corresponding to the marmoset OPC subclusters in the current study (tissue in coarse category, left).

f. Violin plot showing the number of genes detected in each mouse OPC subcluster. Median is annotated (◆).

g. New UMAP scatter plot showing reclustering of the selected mouse OPC clusters (Cluster 9 in lower-right panel of C) colored by new subclusters (right). Using information about the tissue origin provided by the original authors, subclusters were colored and annotated with new labels corresponding to the marmoset OPC subclusters in the current study (tissue label in coarse category, left).

h. Violin plot showing the number of genes detected in each mouse OPC subcluster. Median is annotated (◆).

i – k. Sankey diagrams showing the proportional correspondence between the new labeling (left) and the original labeling (right).

l – n. New UMAP scatter plots showing the result of reclustering after humanizing gene names of each species with a one-to-one ortholog index based on BioMart information to facilitate cross-species analysis. The identity of cells before and after gene label transfer is mostly preserved. Upper left, UMAP colored by cluster identity as annotated before gene name translation (d, e, and g). Upper right, UMAP colored by cluster identity as annotated after gene name translation (used in Fig. 4b). Lower panels, UMAP colored by selected marker-gene expression to identify cycling (*TOP2A*$^{+}$) and differentiating (*ENPP6*$^{+}$) OPC

SupFig 23 - Related to Fig4

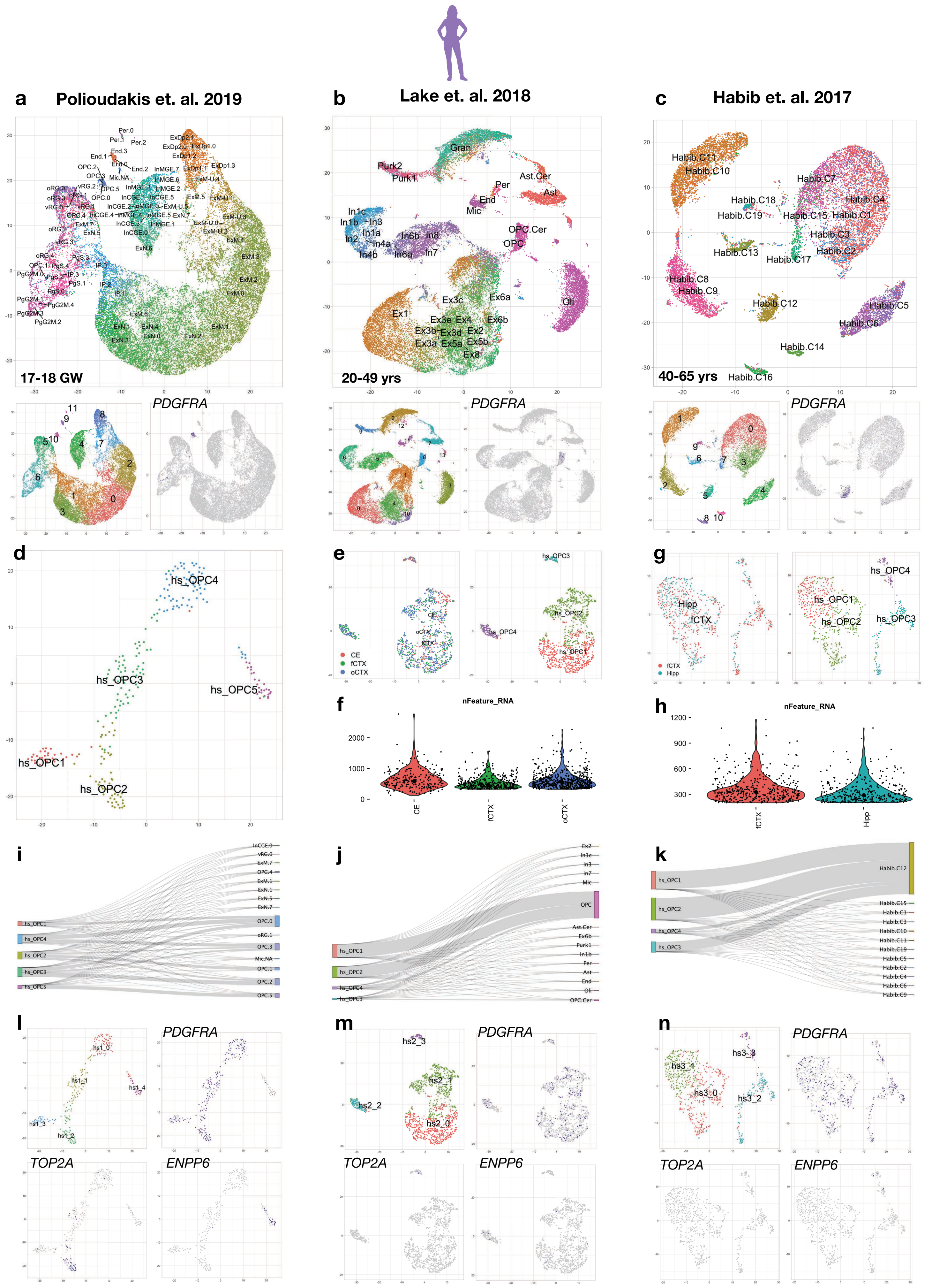

Supplementary Fig. 23. Related to Fig. 4. Reanalysis of human derived oligodendrocyte progenitor cells (OPC).

a – c. Data deposited from published studies in human brain were reanalyzed and reclustered with a pipeline similar to that used in our marmoset study. The original annotation defined by the authors is overlaid onto the UMAP scatter plots in the top row. In the bottom row, for each dataset, the left panel shows our new cluster annotations, and the right panel shows the *PDGFRA*$^{+}$ OPC clusters further analyzed in subsequent panels of this figure, specifically clusters 10 (**a**), 8 (**b**), 5 (**c**).

d. New UMAP scatter plot showing reclustering of the selected human OPC from panel A (Cluster 10) with new labels.

e. New UMAP scatter plot showing reclustering of the selected human OPC from panel B (Cluster 8) colored by new subclusters (right) and tissue type (left).

f. Violin plot showing the number of genes detected in human OPC grouped by tissue type. Median is annotated (◆).

g. New UMAP scatter plot showing reclustering of the selected human OPC from panel C (Cluster 5) colored by new subclusters (right) and tissue type (left).

h. Violin plot showing the number of genes detected in human OPC grouped by tissue type. Median is annotated (◆).

i – k. Sankey diagrams showing the proportional agreement between new labeling (left) and original labeling (right).

l – n. Upper left, UMAP colored by cluster identity as used in Fig. 4b. Other panels, UMAP colored by selected marker-gene expression to identify cycling (*TOP2A*$^{+}$) and differentiating (*ENPP6*$^{+}$) OPC (*PDGFRA*$^{+}$).

SupFig 24 - Related to Fig4

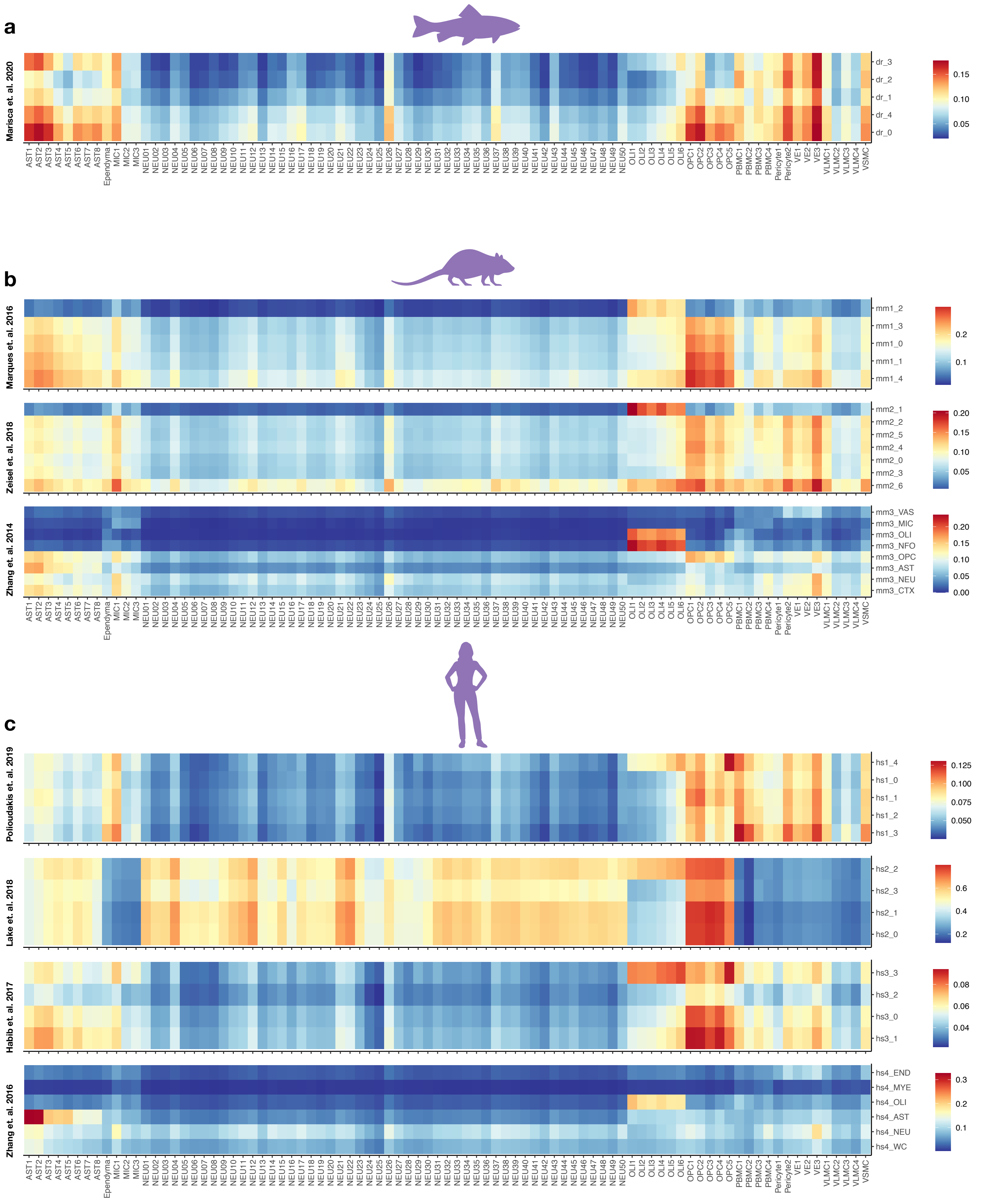

Supplementary Fig. 24. Related to Fig. 4. Cross-cluster comparison of marmoset nuclei to zebrafish, mouse, and human derived oligodendrocyte progenitor cells (OPC).

a – c. Heatmap showing the Pearson's correlation (r) between the 87 marmoset subclusters (C50 object) and newly defined OPC subclusters from zebrafish (*Danio rerio*, dr), mouse (*Mus musculus*, mm), and human (*Homo sapiens*, hs).

SupFig 25 - Related to Fig5

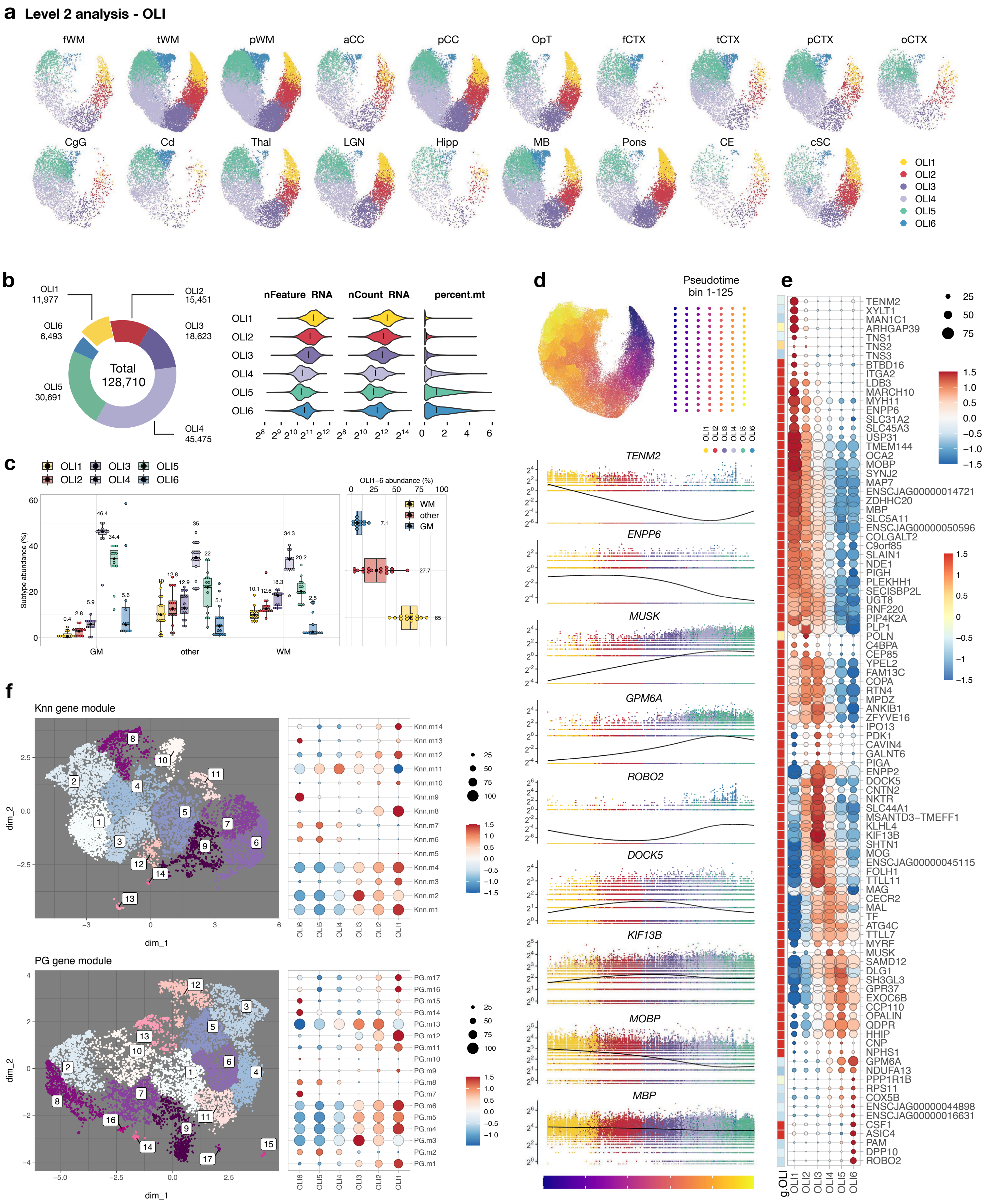

Supplementary Fig. 25. Related to Fig. 5. Nuclei features and gene module analysis in oligodendrocytes (OLI).

a. 2D UMAP scatter-plot visualization of oligodendrocytes (OLI) colored by Level 2 subcluster and split by sampling site.

b. Left, donut plot showing the number of nuclei that passed Level 2 quality control and were annotated in each subcluster. Right, violin plot showing the percentage of RNA reads that mapped to the mitochondria genome (percent.mt), the number of RNA molecules detected (nCount_RNA), and the number of RNA species (nFeature_RNA) in each cluster. Median is annotated (–).

c. Box plot showing the relative abundance of OLI subclusters in each tissue type (coarse category; see Fig. 1d for list). Putative early (young) populations (OLI1 and OLI2, *ENPP6*^{high}) are low in GM compared to WM and other tissue types. OLI4 is the major cluster in all tissue types. n = 42 independent samples; median is annotated (black diamond shape) and listed. The lower and upper hinges of the box plot correspond to the 25th and 75th percentiles, whiskers extend from the hinges to maxima or minima at most 1.5 times inter-quartile range.

d. Top, 2D UMAP scatter-plot visualization of OLI colored by pseudotime. Bottom, jitter plots of gene expression as a function of pseudotime colored by OLI subcluster identity.

e. Heatmap and dot plot showing mean-centered and z-score-scaled marker gene expression in global space (heatmap, OLI average expression among Level 1 classes) and local space (dot plot, Level 2 OLI subclusters). The portion of the heatmap that represents global OLI (g.OLI) is reproduced here from the full heatmap shown in **Supplementary Fig. 27b**.

f. UMAP scatter-plot visualization of genes grouped by expression similarity across cells. Genes that passed Moran's *I* statistic spatial test (< 5% FDR) over the *k*-nearest neighbor graph (Knn, k=25, left), or over the trajectory learned principal graph (PG, right), by Monocle3 graph_test function. Genes are grouped and colored by modules identified in each graph test by find_gene_modules function with resolution of 0.001. The list of genes of each module was aggregated through Seurat v3 AddModuleScore function. Dot plot showing the averaged and scaled expression of each module gene set across OLI subclusters.

SupFig 26 - Related to Fig5

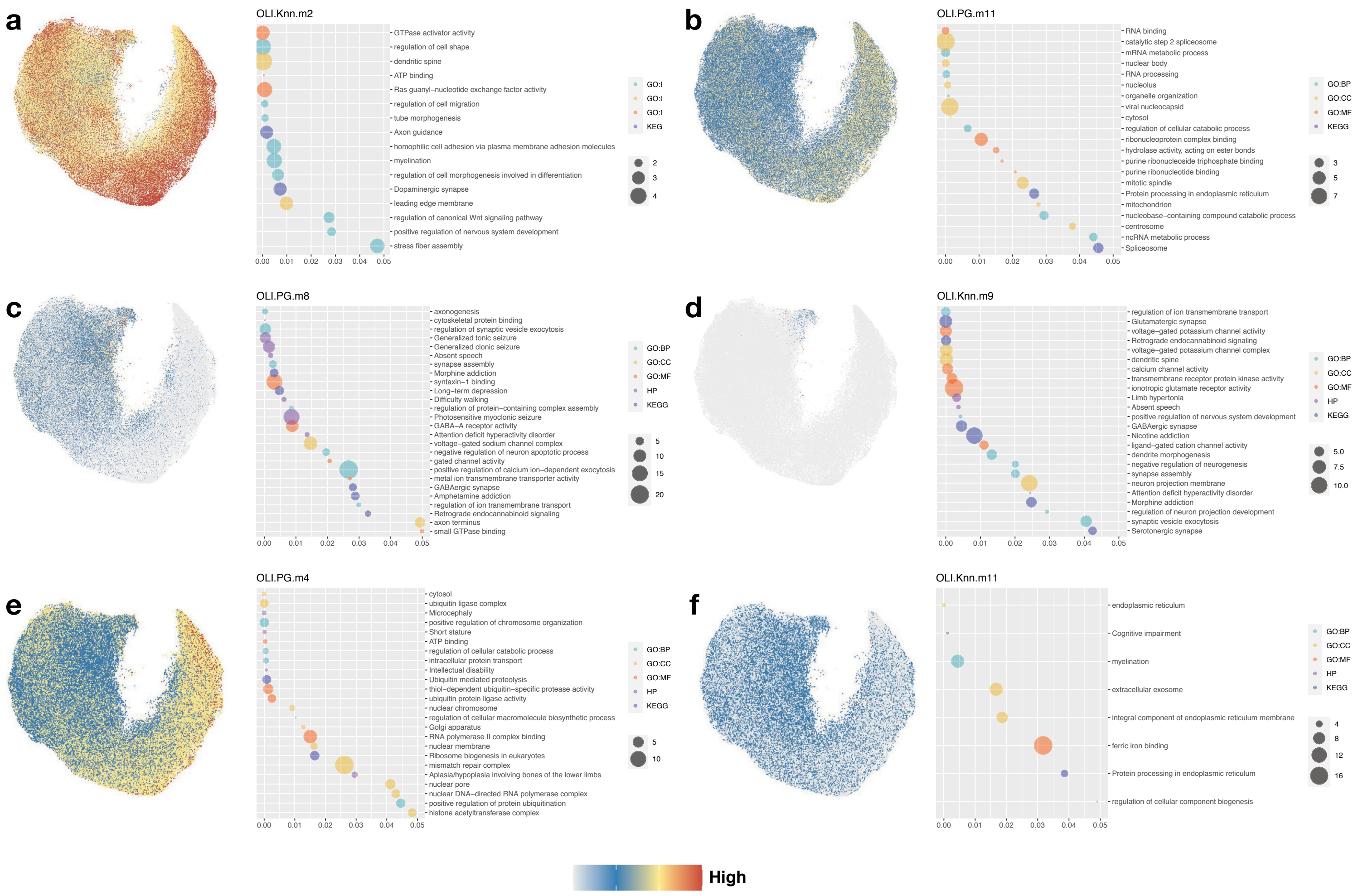

Supplementary Fig. 26. Related to Fig. 5. GO analysis on selected gene modules in oligodendrocytes.

a – f. UMAP scatter-plot visualization of nuclei colored by averaged expression of each module (color bar is in the center of the bottom row). Dot plot showing the enriched GO terms from the list of genes in each selected module. GO, gene ontology; BP, biological process; CC, cellular component; MF, molecular function; HP, human phenotype ontology; KEGG, Kyoto Encyclopedia of Genes and Genomes.

See also Supplementary Data 2 and 4.

SupFig 27 - Related to Fig5

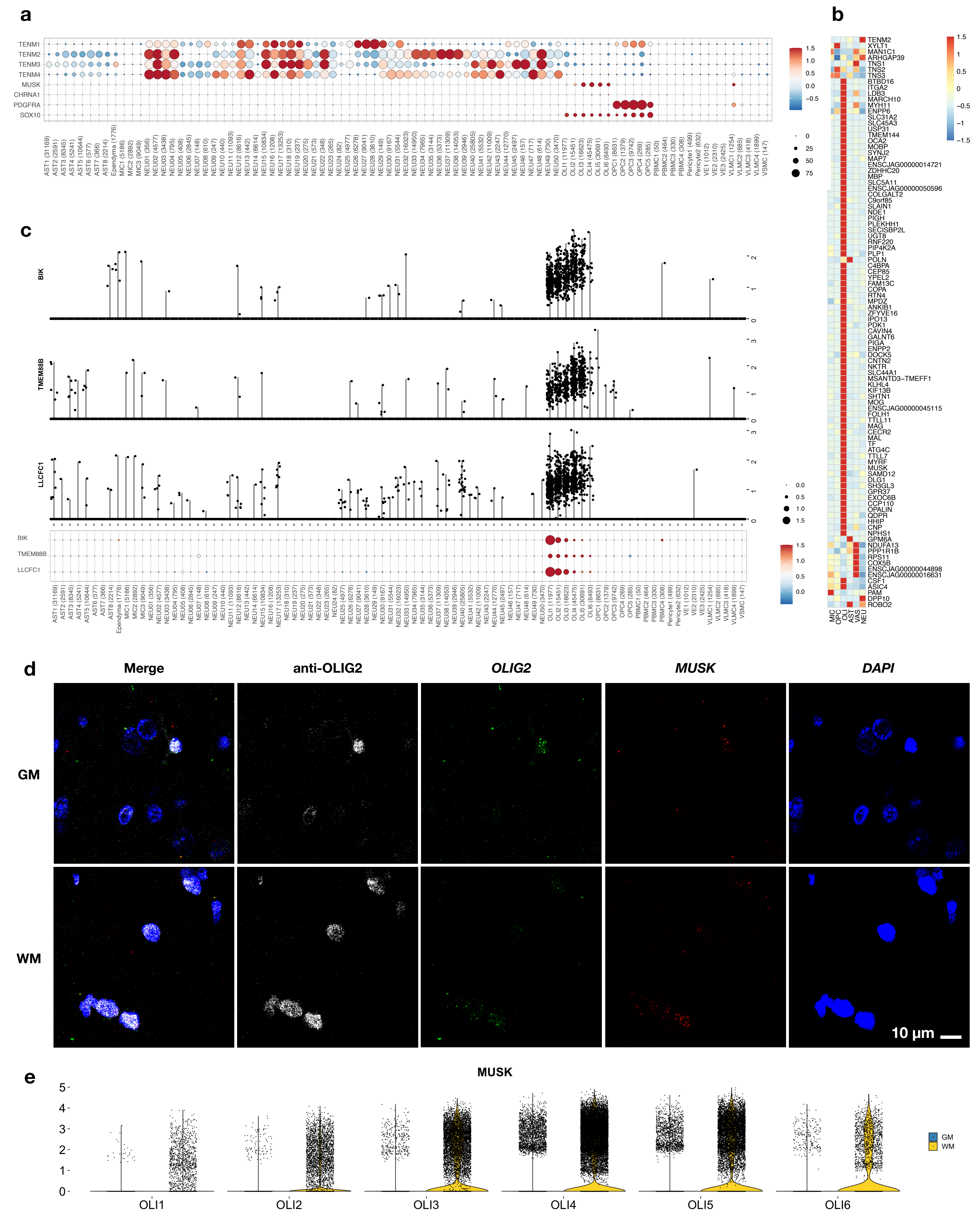

Supplementary Fig. 27. Related to Fig. 5. Selected gene expression in oligodendrocytes.

- **a.** Dot plot showing the expression across all 87 subclusters of teneurin family (*TENM1 – 4*), genes associated with muscle weakness (*MUSK*, *CHRNA1*), and oligodendrocyte lineage/OPC genes (*SOX10*, *PDGFRA*).
- **b.** Heatmap showing the mean-centered and z-score-scaled OLI marker-gene expression across Level 1 classes. The same object (C50 object) was used to make plots shown in Supplementary Fig. 25e.
- **c.** Dot plot showing the expression of rare and understudied genes (*BIK*, *TMEM88B*, and *LLCFC1*) enriched in OLI.
- **d.** The expression of MUSK is detected in OLIG2+ cells in adult marmoset brain by combined immunofluorescent staining and fluorescent in situ hybridization (Hybridization chain reaction v3.0). Experiment was repeated independently for 3 times with similar results.
- **e.** Violin plot showing the expression of MUSK across OLI subclusters in GM and WM.

SupFig 28 - Related to Fig5

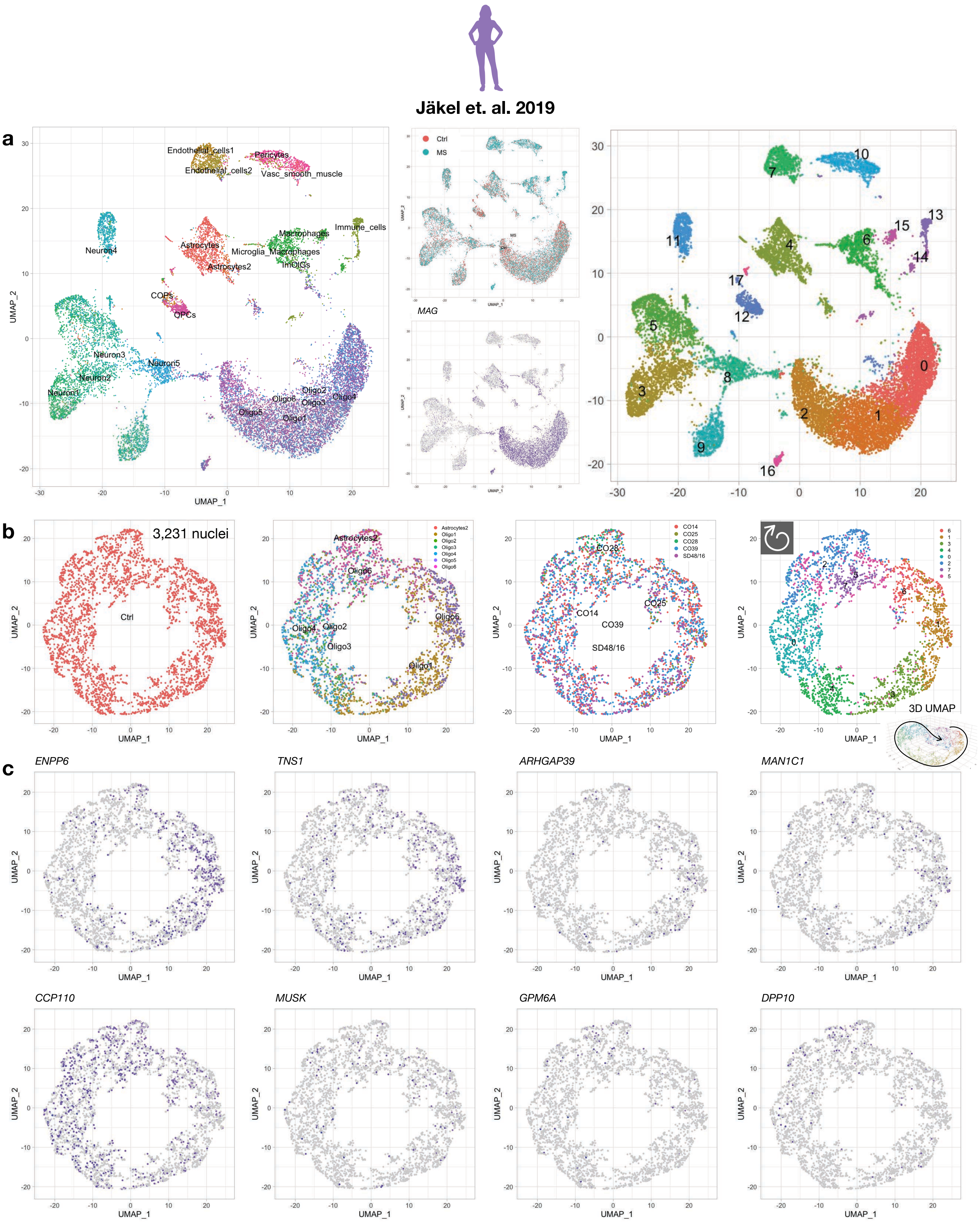

Supplementary Fig. 28. Related to Fig. 5. Adult human oligodendrocytes are arranged into a spiral pattern in UMAP space.

- **a.** Previously published and deposited data were reanalyzed and reclustered with a pipeline similar to that used in our marmoset study. UMAP scatter-plot visualization of cells colored by the original annotations defined by the authors (left, cell type; middle top, tissue source, control [Ctrl] or multiple sclerosis [MS]). UMAP plot of human oligodendrocytes colored by *MAG* expression to facilitate subset selection (middle bottom). UMAP plot colored by newly defined cluster number (right).
- **b.** Newly defined clusters 0, 1, 2 from control samples (excluding samples from MS patients) were selected for further subset analysis. UMAP scatter-plot visualization of cells colored by the original annotations defined by the authors (first three panels) and newly defined cluster numbers (right-most panel). 3D UMAP scatter-plot visualization of the nuclei colored by the newly defined cluster numbers (right bottom).
- **c.** UMAP scatter-plot visualization of human oligodendrocyte lineage cells colored by selected marker-gene expression.

SupFig 29 - Related to Fig5

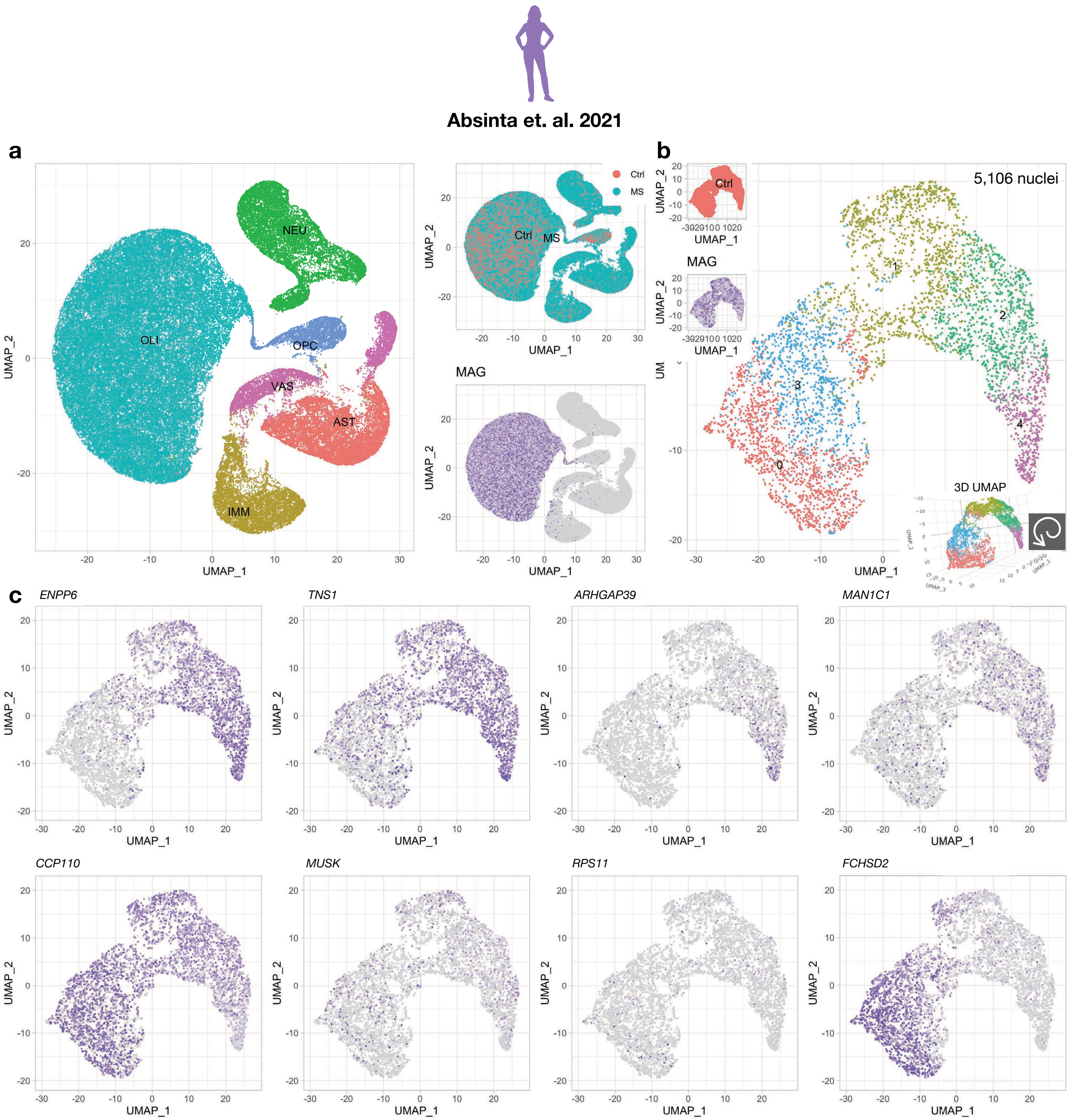

Supplementary Fig. 29. Related to Fig. 5. Adult human oligodendrocytes are arranged into a spiral pattern in UMAP space.

- **a.** Previously published and deposited data were reanalyzed and reclustered with a pipeline similar to that used in our marmoset study. UMAP scatter-plot visualization of cells colored by cell class (left), tissue source (top right; Ctrl, control; MS, multiple sclerosis), and the expression of MAG (bottom right) to facilitate subset selection (middle bottom).
- **b.** Oligodendrocytes from control human tissue were reanalyzed; the UMAP plot is colored by newly defined cluster number.
- **c.** UMAP scatter-plot visualization of human oligodendrocyte lineage cells colored by selected marker-gene expression.

SupFig 30 - Related to Fig5

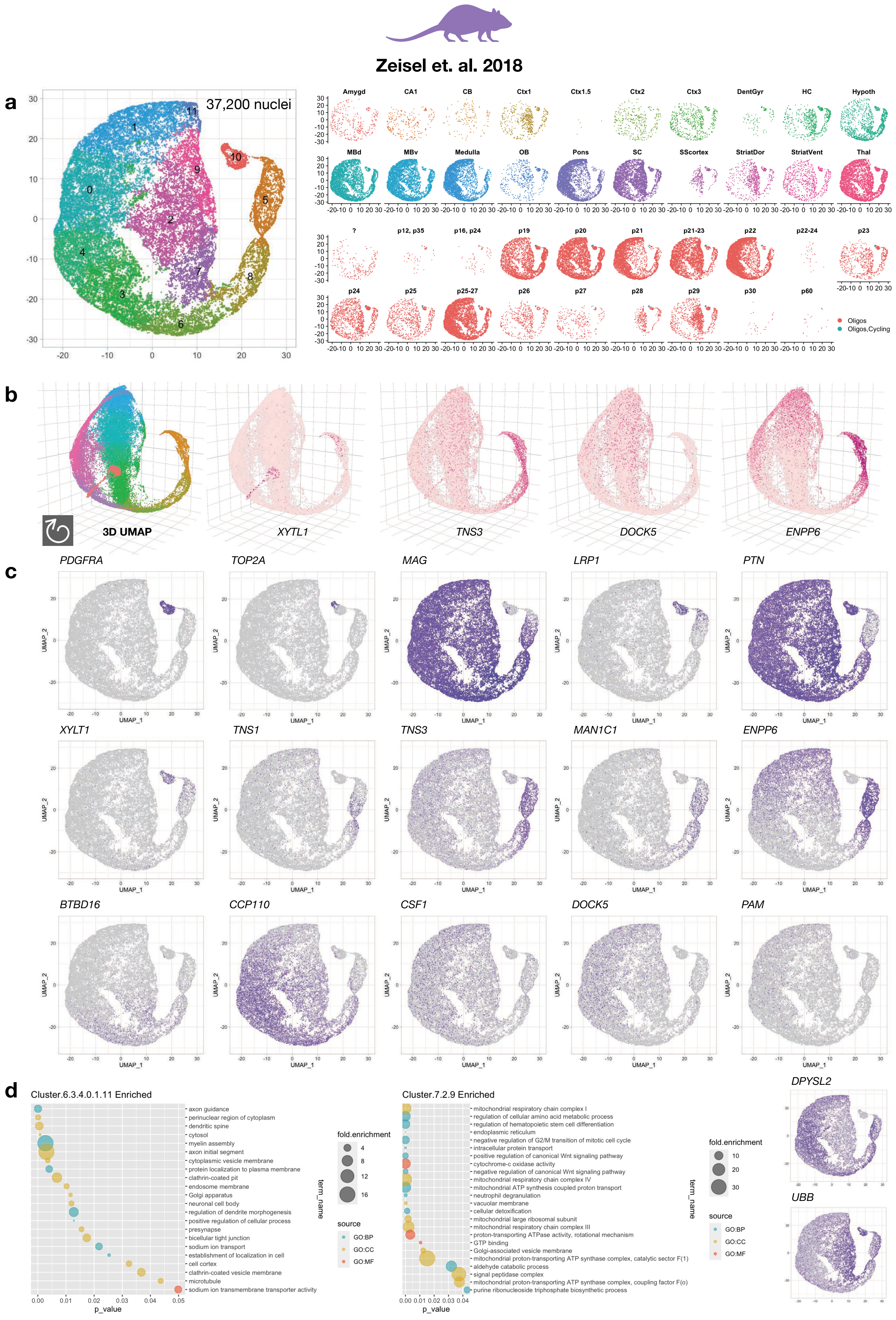

Supplementary Fig. 30. Related to Fig. 5. Mouse oligodendrocyte lineage cells are arranged into a spiral pattern in UMAP space along a putative differentiation trajectory.

- **a.** Previously published and deposited data were reanalyzed and reclustered with a pipeline similar to that used in our marmoset study. New UMAP scatter plots showing the result of reclustering after humanizing gene names of mouse with a one-to-one ortholog index based on BioMart information to facilitate cross-species analysis. UMAP scatter-plot visualization of cells colored by newly defined cluster numbers (left). The annotations defined by the original authors, including sampling source (top right) and age (bottom right), were overlaid onto the UMAP scatter plots.
- **b.** 3D UMAP scatter-plot visualization of mouse nuclei colored by the newly defined cluster numbers, highlighting expression of four selected marker genes.
- **c.** UMAP scatter-plot visualization of mouse oligodendrocyte lineage cells colored by selected marker-gene expression.
- **d.** Dot plot showing the enriched GO terms from the list of differentially expressed genes between left (combining mouse OLI subclusters 6, 3, 4, 0, 1, 11) and right (combining subclusters 7, 2, 9) branches. Left, GO terms related to genes increased in the left branch, including pathways required for myelin formation and neural cell interaction. Middle, GO terms related to genes increased in the right branch, including pathways related to mitochondrial and metabolic functions. Right, UMAP scatter-plot visualization of oligodendrocyte lineage cells colored by markers enriched in each branch. *UBB* expression is elevated in the subset of oligodendrocyte lineage cells (right branch), indicating active protein turnover.

SupFig 31 - Related to Fig5

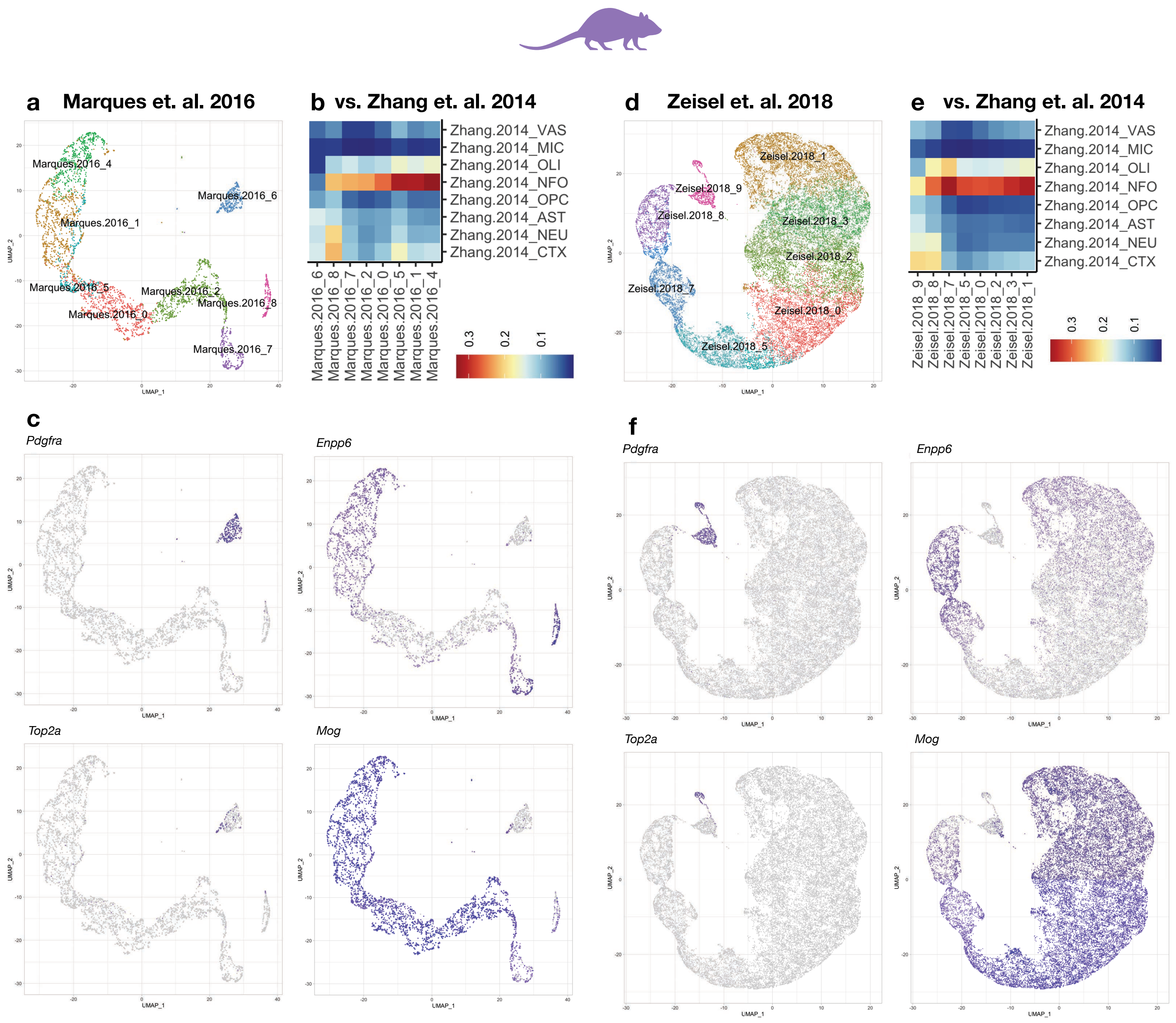

Supplementary Fig. 31. Related to Fig. 5. Cross-dataset comparison of mouse oligodendrocyte lineage cells.

- **a.** Previously published and deposited data were reanalyzed and reclustered with a pipeline similar to that used in our marmoset study. UMAP scatter-plot visualization of cells colored by newly defined cluster numbers. Clusters are reordered from young to old (Marques.2016_6, 8, 7, 2, 0, 5, 2, 4) along the putative trajectory of oligodendrocyte lineage cell differentiation.
- **b.** Heatmap showing the Pearson's correlation (r) of transcriptome between 8 subclusters of oligodendrocyte lineage cells from Marques et. al. 2016 and 8 types of immunopanned cells from Zhang et. al 2014 (VAS, endothelial cell; MIC, immune cell; OLI, myelinating oligodendrocyte; NFO, newly formed oligodendrocyte; OPC, oligodendrocyte progenitor cell; AST, astrocyte; NEU, neuron; CTX, unpurified total cell from cortex). NFO identified by immunopanning (Zhang.2014_NFO) correlate highest with the putative "older" oligodendrocyte (Marques.2016_5, 1, 4) identified by single cell analysis.
- **c.** UMAP scatter-plot visualization of mouse oligodendrocyte lineage cells from Marques et. al. 2016 colored by *Pdgfra*, *Enpp6*, *Top2a*, and *Mag* expression.
- **d.** Previously published and deposited data were reanalyzed and reclustered with a pipeline similar to that used in our marmoset study. Putative stressed oligodendrocytes (*UBB*^{high}) were removed from the previous analysis in Supplementary Fig. 30d and reclustered. UMAP scatter-plot visualization of cells colored by newly defined cluster numbers. Clusters are reordered from young to old (Zeisel.2018_9, 8, 7, 5, 0, 2, 3, 1) along the putative trajectory of oligodendrocyte lineage cell differentiation.
- **e.** Heatmap showing the Pearson's correlation (r) of transcriptome between 8 subclusters of oligodendrocyte lineage cells from Zeisel et. al. 2018 and 8 types of immunopanned cells from Zhang et. al 2014. NFO identified by immunopanning (Zhang.2014_NFO) highly correlate with both putative "older" (Zeisel.2018_1) and "younger" (Zeisel.2018_7) oligodendrocyte identified by single cell analysis.
- **f.** UMAP scatter-plot visualization of mouse oligodendrocyte lineage cells from Zeisel et. al. 2018 colored by *Pdgfra*, *Enpp6*, *Top2a*, and *Mag* expression.

SupFig 32 - Related to Fig6

a Combine AST and VAS for L2 QC

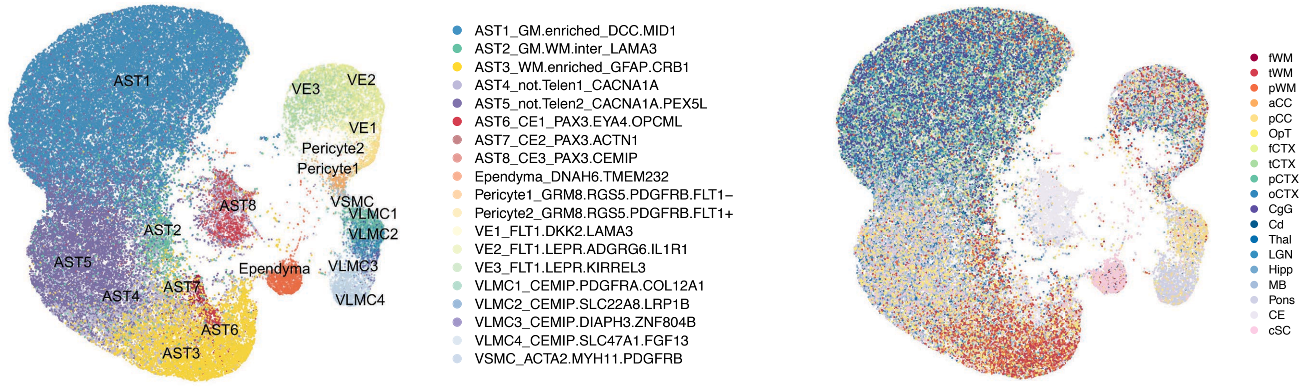

b

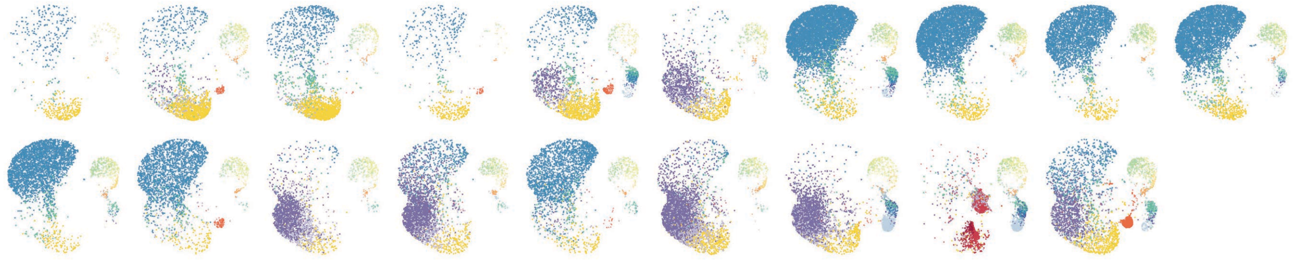

c

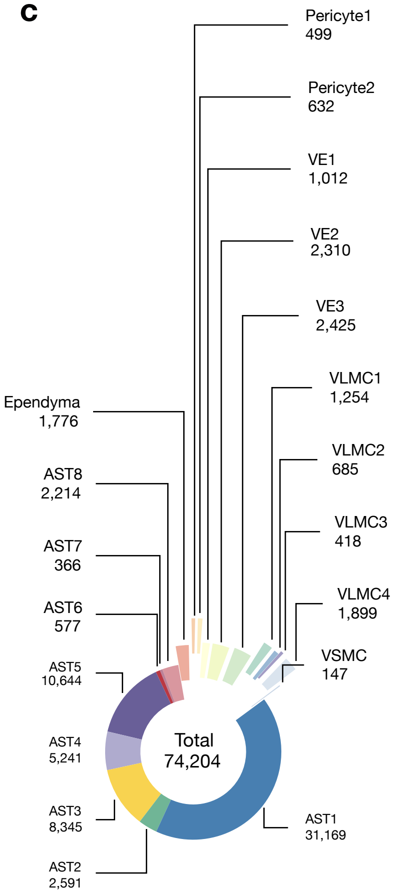

d

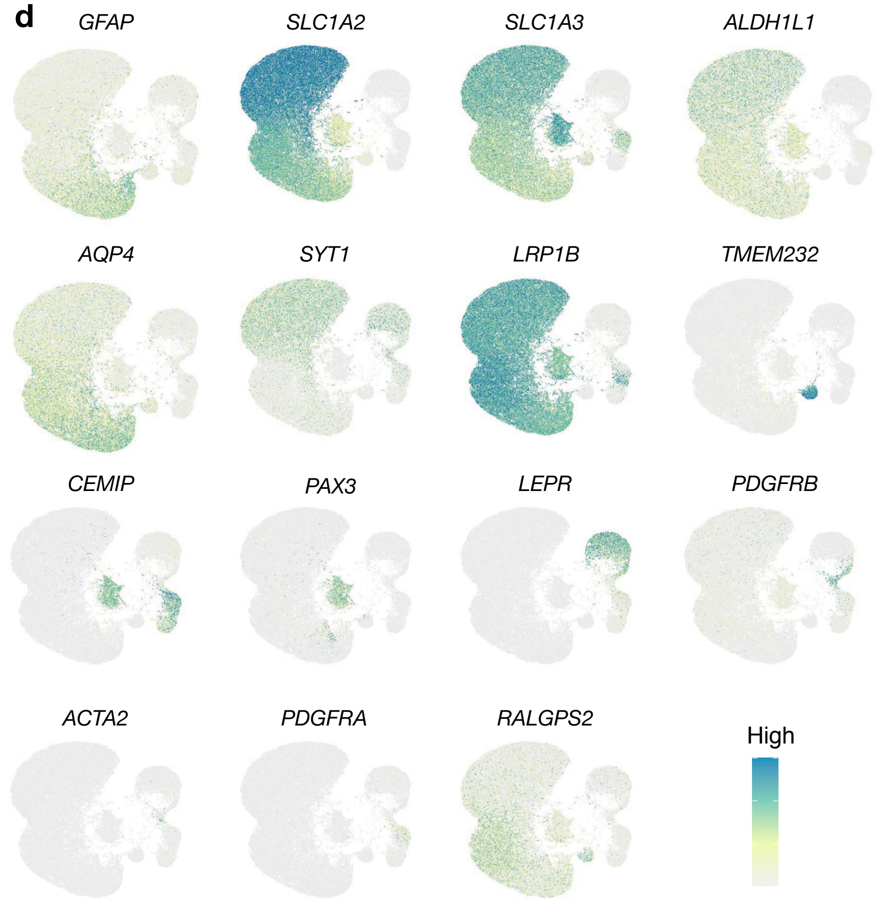

Supplementary Fig. 32. Related to Fig. 6. Nuclei features in cells at the barriers of the CNS.

- **a.** UMAP scatter-plot visualization of combined astrocyte/vascular (AST/VAS) nuclei, colored by subcluster (upper left) and sampling site (upper right).
- **b.** UMAP scatter-plot visualization of AST/VAS subclusters split by sampling site, see Supplementary Fig. 32a for label.
- **c.** Donut plot showing the number of AST/VAS nuclei that passed Level 2 quality control and were annotated into subclusters.
- **d.** UMAP scatter-plot visualization of AST/VAS nuclei colored by selected marker gene expression.

SupFig 33 - Related to Fig6

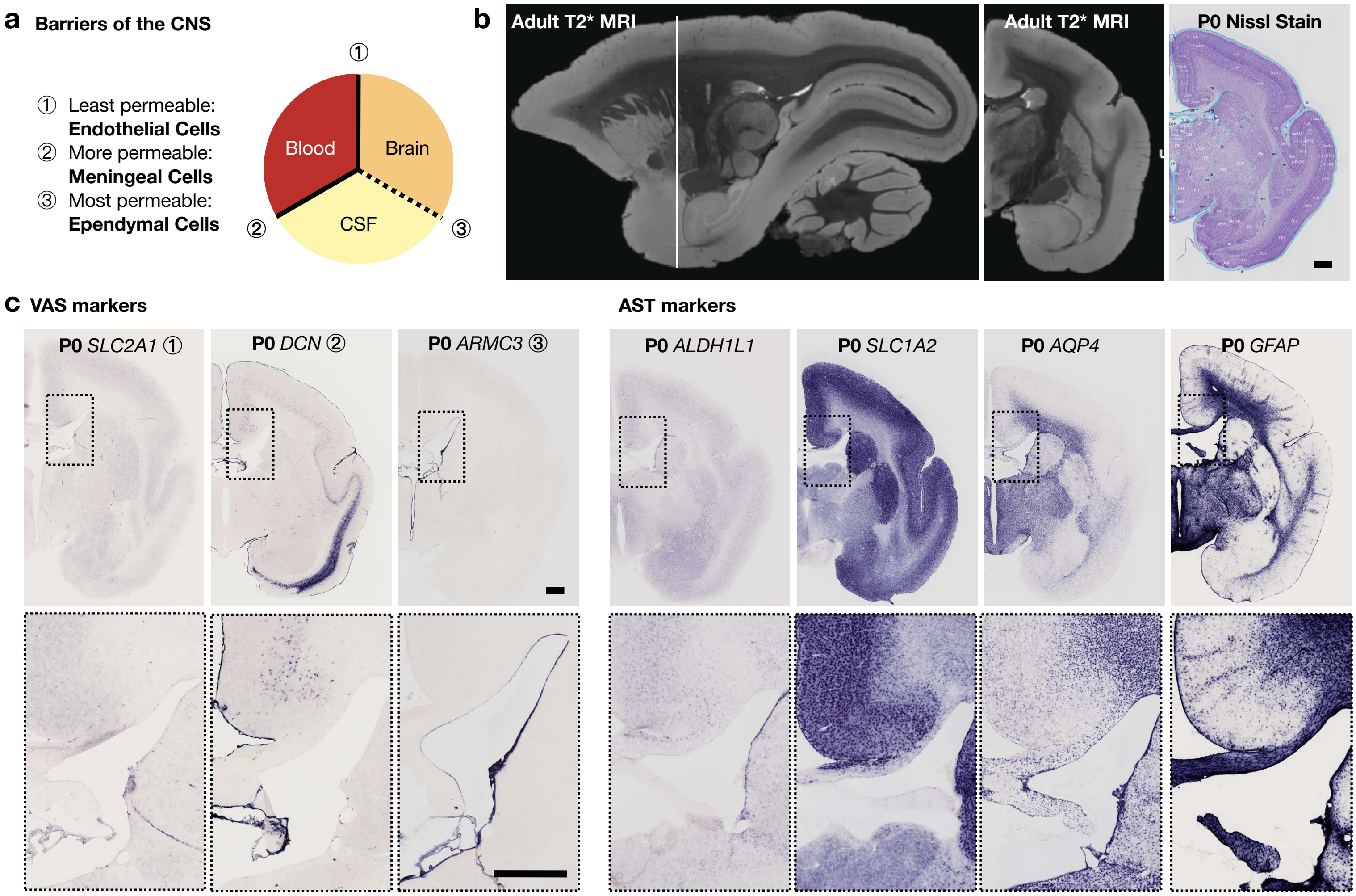

Supplementary Fig. 33. Related to Fig. 6. Cell types and their gene expression patterns at the barriers of the CNS.

a. Schematic illustration of the barriers of the CNS. The least permeable blood-brain barrier (BBB, ①) is formed mostly by vascular endothelial cells, the somewhat more permeable blood-CSF barrier (②) by meningeal cells, and the most permeable CSF-brain barrier (ventricle, ③) by ependymal cells[6,7].

b. Approximate cross-sectional plane (white line) indicated on a sagittal view of an adult marmoset postmortem T2*-weighted MRI (left). Coronal view of the same adult marmoset T2*-weighted MRI (middle). Coronal histological section of a P0 marmoset stained with Nissl's method (right) and anatomically matched to the adult coronal T2*- weighted MRI slice.

c. Selected in situ hybridization (ISH) of genes enriched in endothelial, meningeal, and ependymal cells (left, VAS markers) and astrocytes (right, AST markers). Bottom row, enlarged areas are indicated by the box with dashed edge. Scale bar, 1mm. MRI scans are derived from the Marmoset Brain Mapping database (https:// marmosetbrainmapping.org/)[1,2], and Nissl stain and ISH images from the Marmoset Gene Atlas (https://gene-atlas.brainminds.riken.jp/)[3,4].

SupFig 34 - Related to Fig6

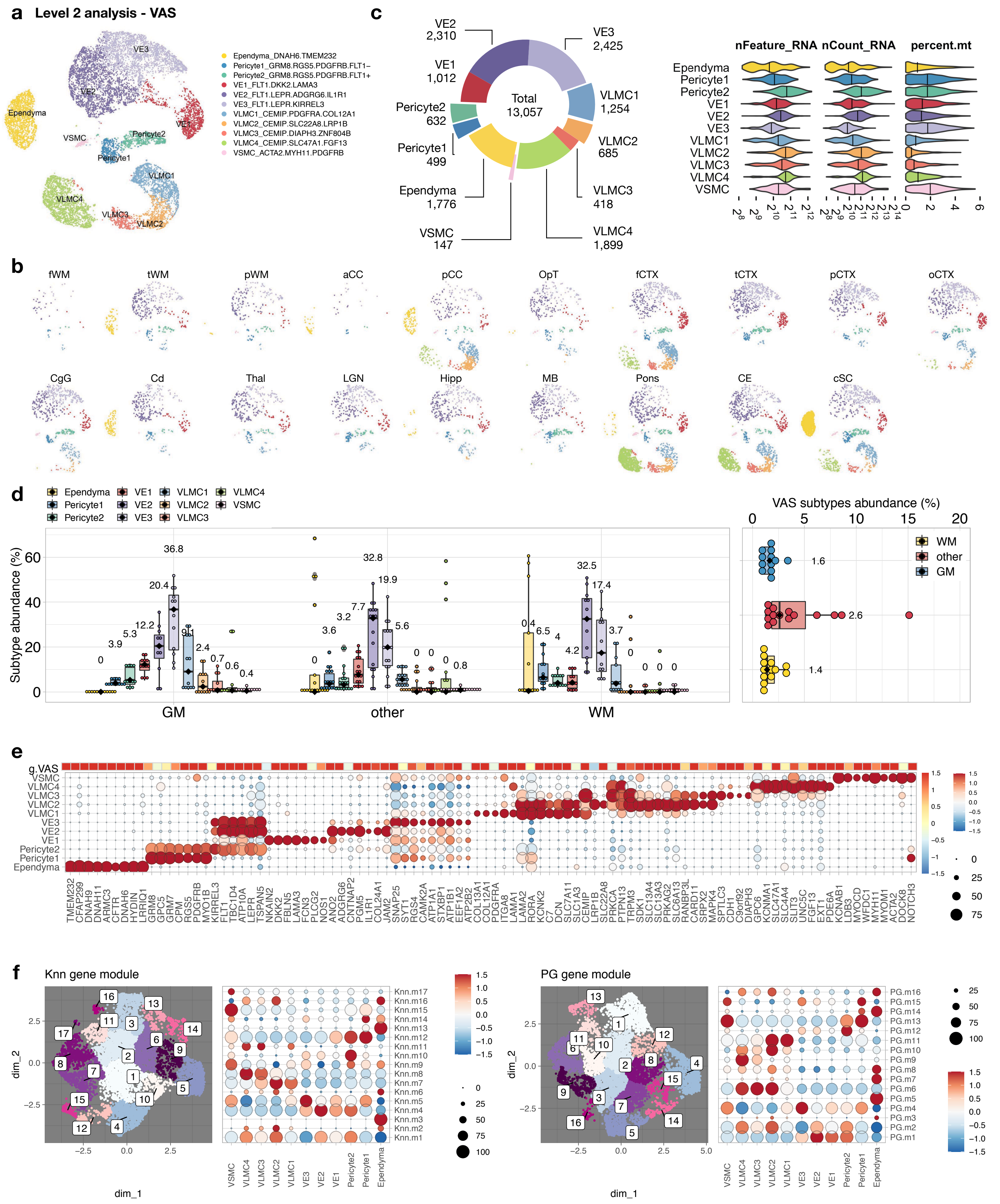

Supplementary Fig. 34. Related to Fig. 6. Nuclei features, gene expression, and gene modules in vascular and other cells at the CNS barrier cells (VAS).

a. Local UMAP scatter-plot visualization of cell types enriched at the CNS barrier colored by subcluster identity.

b. UMAP colored by subcluster identity and split by sampling site.

c. Left, donut plot showing the number of nuclei that passed Level 2 quality control and were annotated into subclusters. Right, violin plot showing the percentage of RNA reads that mapped to mitochondria genome (percent.mt), the number of RNA molecules detected (nCount_RNA), and the number of RNA species (nFeature_RNA) in each cluster. Median is annotated (–).

d. Box plot showing the relative abundance of VAS subclusters in each coarse tissue category (see Supplementary Fig. 1d for full list). n = 42 independent samples; median is annotated (black diamond shape) and listed. The lower and upper hinges of the box plot correspond to the 25th and 75th percentiles, whiskers extend from the hinges to maxima or minima at most 1.5 times inter-quartile range.

e. Heatmap and dot plot showing mean-centered and z-score-scaled marker gene expression in global space (heatmap, VAS average expression among Level 1 classes, C50) and local space (dot plot, VAS subclusters). The portion of the heatmap that represents global VAS (g.VAS) is reproduced here from the full heatmap shown in Supplementary Fig. 35b. Dot size indicates the percentage of nuclei that expressed each gene in each subcluster. Scaling is relative to expression across all VAS nuclei in which a given gene was detected.

f. UMAP scatter plot visualization of genes grouped by expression similarity across cells. Genes that passed the Moran's *I* statistic spatial test (< 5% FDR) over *k*-nearest neighbor graph (Knn, k=25, left), or the over trajectory learned principal graph (PG, right), by Monocle3 graph_test function. Genes are grouped and colored by modules identified in each graph test by find_gene_modules function with resolution of 0.001. The list of genes in each module was aggregated through Seurat v3 AddModuleScore function. Dot plot showing the averaged and scaled expression of each module's gene set across VAS subclusters.

SupFig 35 - Related to Fig6

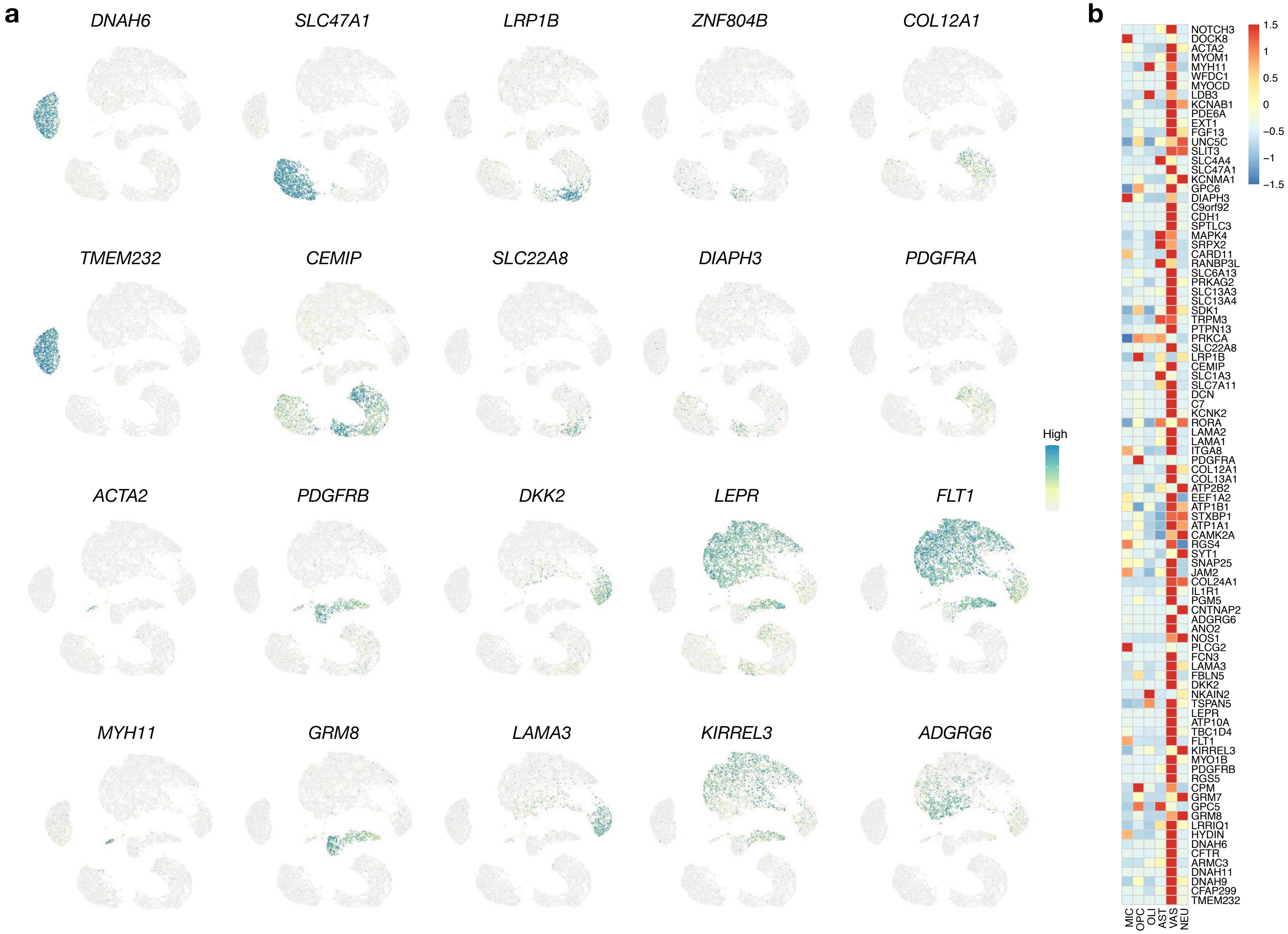

Supplementary Fig. 35. Related to Fig. 6. Further assessment of nuclei features and gene module analysis of vascular and other cells at the CNS barriers (VAS).

- **a.** UMAP scatter-plot visualization of VAS nuclei colored by selected marker-gene expression.
- **b.** Heatmap showing mean-centered and z-score-scaled VAS marker gene expression across Level 1 clusters.

SupFig 36 - Related to Fig6

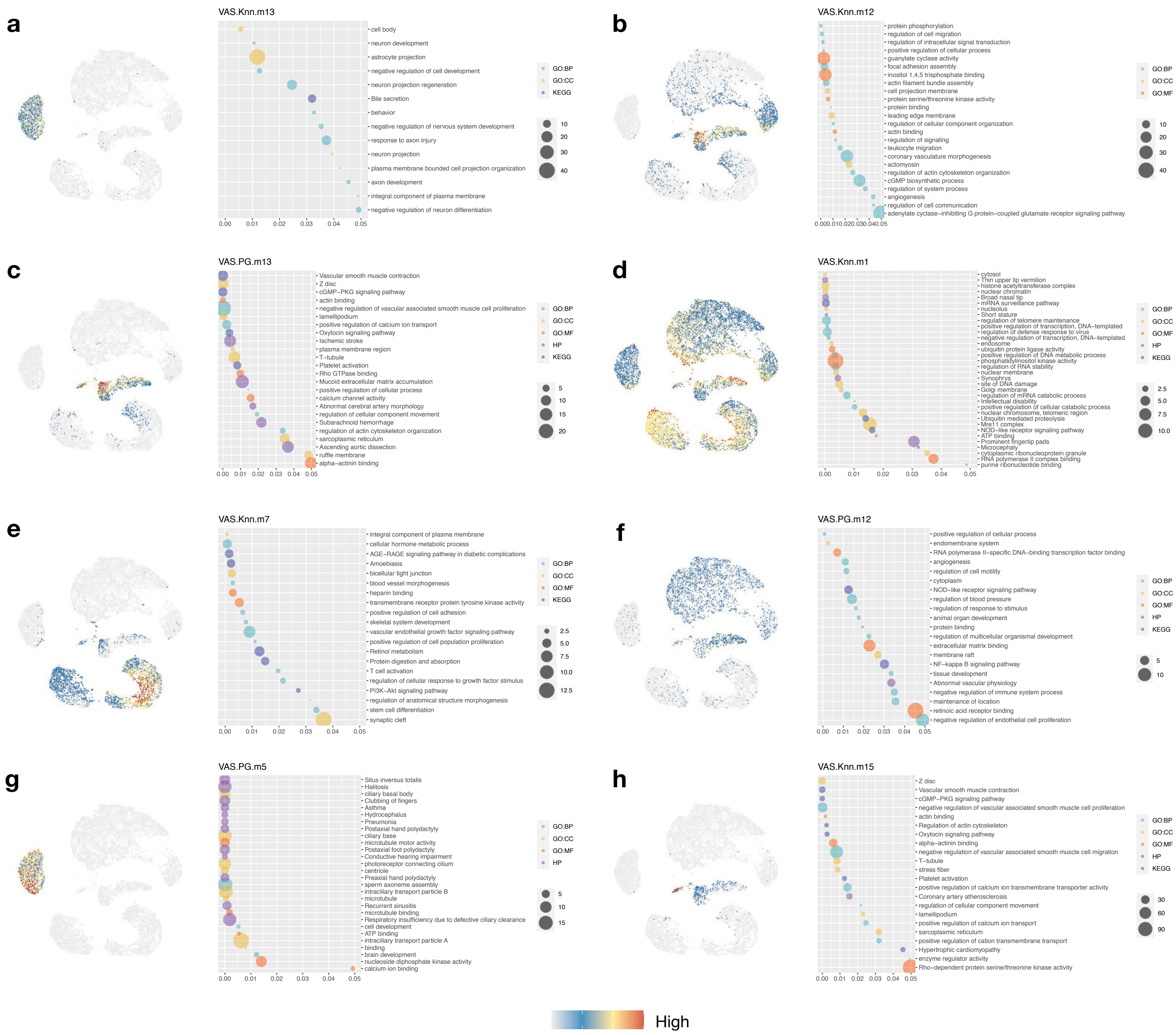

*

Supplementary Fig. 36. Related to Fig. 6. The expression of gene modules in VAS subclusters.

a – h. Left, UMAP scatter plot visualization of VAS nuclei colored by aggregated expression of genes in each module. Right, dot plot showing the enriched GO terms from the list of genes in each module. GO, gene ontology; BP, biological process; CC, cellular component; MF, molecular function; HP, human phenotype ontology; KEGG, Kyoto Encyclopedia of Genes and Genomes.

SupFig 37 - Related to Fig6

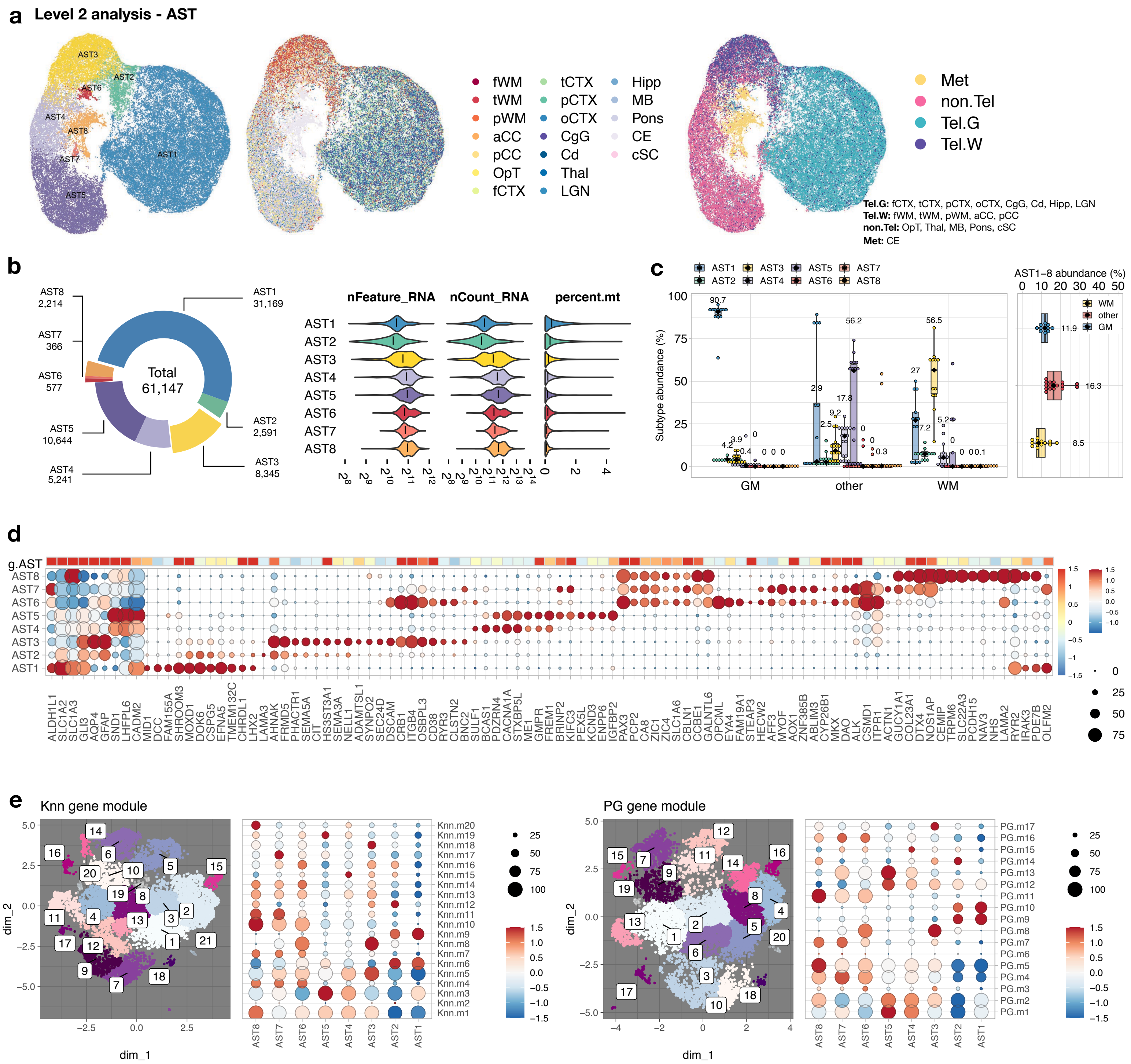

Supplementary Fig. 37. Related to Fig. 6. Assessment of nuclei features and gene module analysis of astrocytes (AST).

a. UMAP scatter-plot visualization of astrocytes (AST) colored by subcluster identity, fine tissue type, and developmental tissue types. **Tel.G**, telencephalon gray; **Tel.W**, telencephalon white; **non.Tel**, non-telencephalon; **Met**, metencephalon.

b. Left, donut plot showing the number of nuclei that passed the Level 2 quality control and were annotated into AST subclusters. Right, violin plot showing the percentage of RNA reads that mapped to mitochondria genome (percent.mt), the number of RNA molecules detected (nCount_RNA), and the number of RNA species (nFeature_RNA) in each cluster. Median is annotated (–).

c. Box plot showing the relative abundance of AST subclusters in each coarse sampling category (coarse category; see Fig. 1d). n = 42 independent samples; median is annotated (black diamond shape) and listed. The lower and upper hinges of the box plot correspond to the 25th and 75th percentiles, whiskers extend from the hinges to maxima or minima at most 1.5 times inter-quartile range.

d. Heatmap and dot plot showing mean-centered and z-score-scaled marker gene expression in global space (heatmap, AST average expression among Level 1 classes, C50) and local space (dot plot, AST subclusters). The portion of the heatmap that represents global AST (g.AST) is reproduced here from the full heatmap shown in Supplementary Fig. 38b.

e. UMAP scatter-plot visualization of genes grouped by expression similarity across cells. Genes that passed Moran's *I* statistic spatial test (< 5% FDR) over the *k*-nearest neighbor graph (Knn, k=25, left), or over the trajectory learned principal graph (PG, right), by Monocle3 graph_test function. Genes are grouped and colored by modules identified in each graph test by find_gene_modules function with resolution of 0.001. The list of genes in each module was aggregated through Seurat v3 AddModuleScore function. Dot plot showing the averaged and scaled expression of each module's gene set across AST subclusters.

SupFig 38 - Related to Fig6

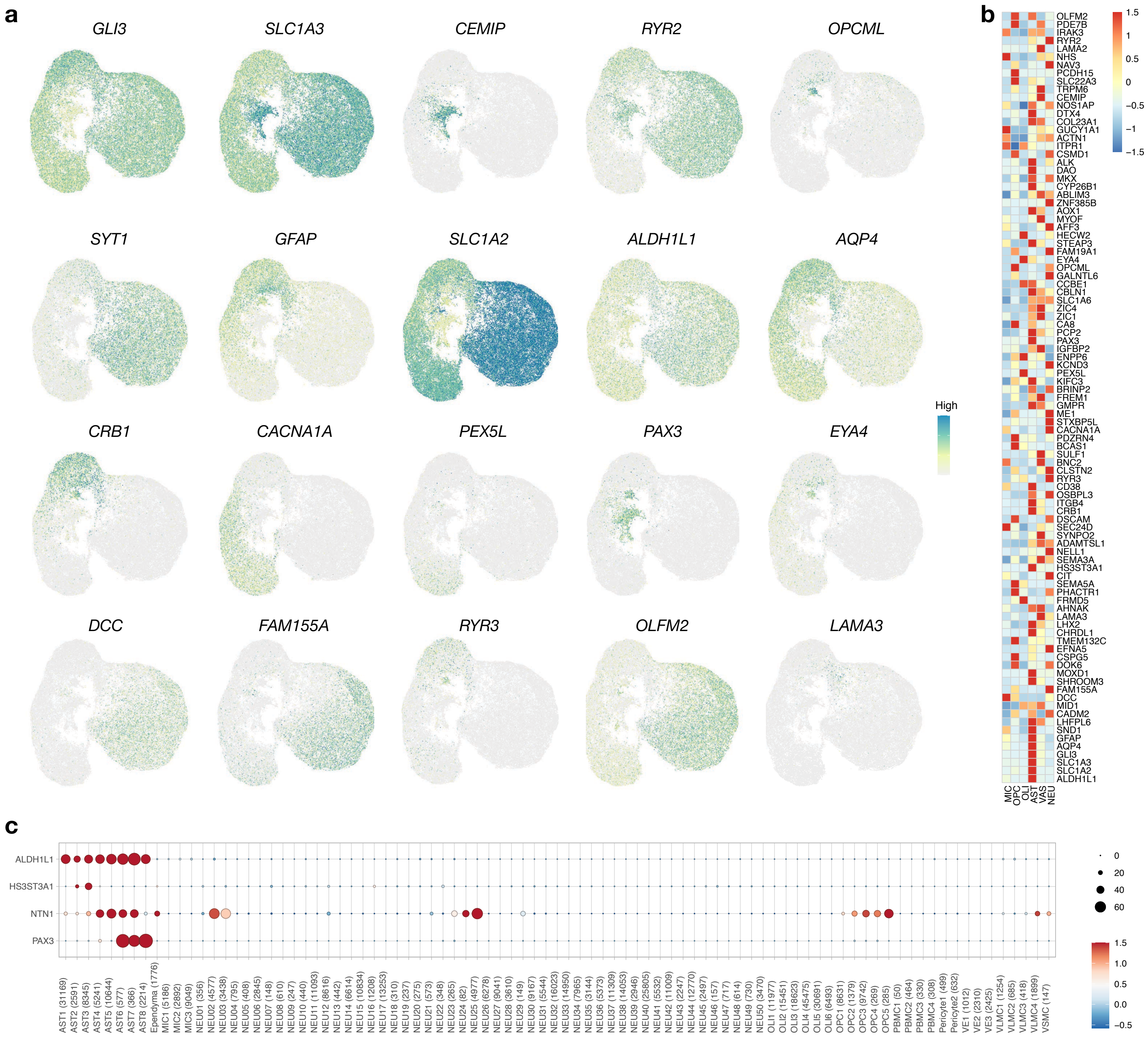

Supplementary Fig. 38. Related to Fig. 6. Selected gene expression in astrocytes and compared across 6 cell classes.

- **a.** UMAP scatter-plot visualization of astrocytes colored by selected marker gene expression.
- **b.** Heatmap showing mean-centered and z-score-scaled AST marker gene expression across Level 1 classes. The same object (C50 object) was used to make plots shown in Supplementary Fig. 37e.
- **c.** Dot plot showing selected mean-centered and z-score-scaled expression of selected subcluster-defining AST marker genes.

SupFig 39 - Related to Fig6

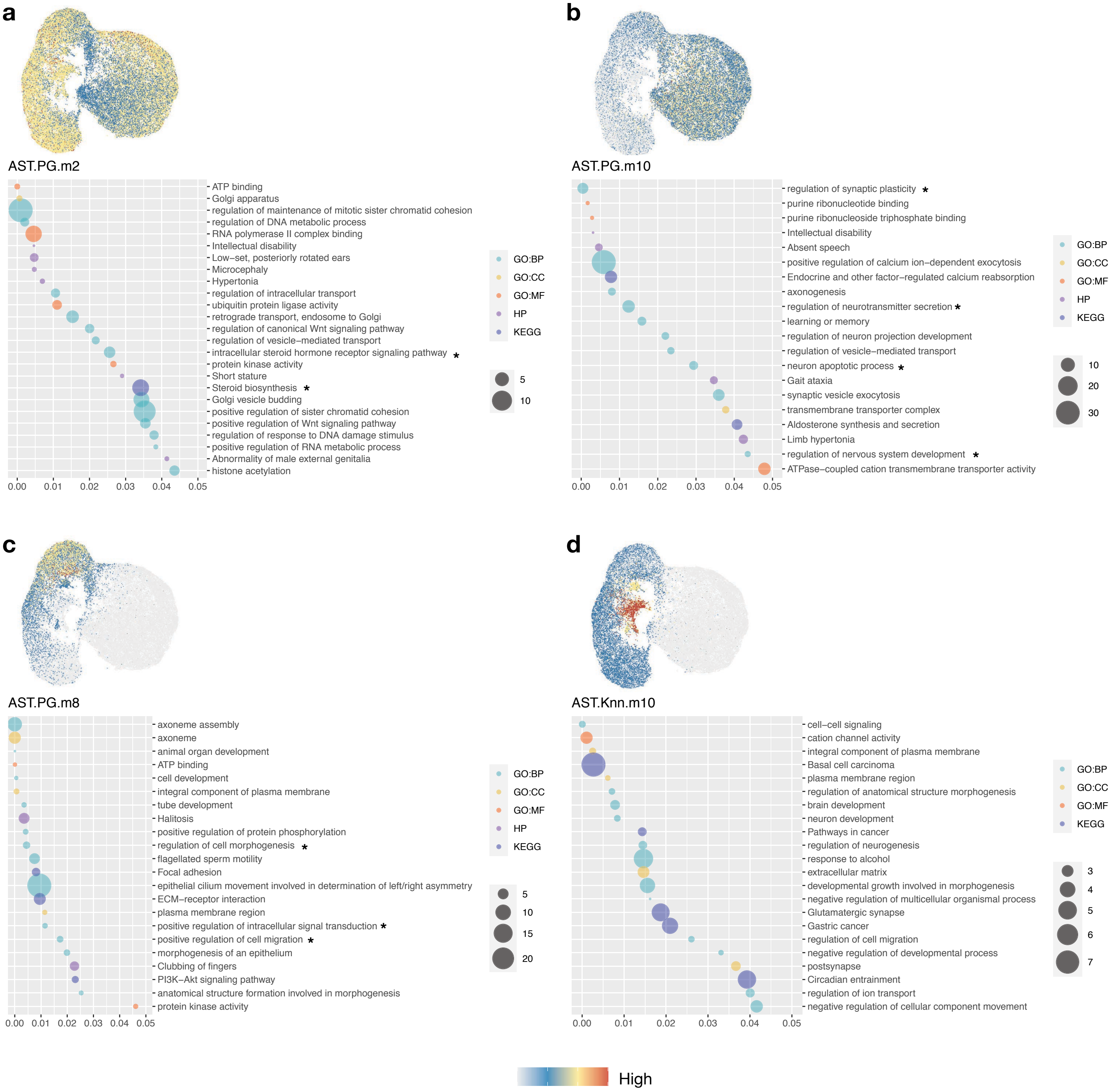

Supplementary Fig. 39. Related to Fig. 6. GO analysis on selected gene modules in astrocytes.

a – d. Top, UMAP scatter plot visualization of AST nuclei colored by aggregated expression of genes in each module. Bottom, dot plot showing the enriched GO terms from the list of genes in each module. GO, gene ontology; BP, biological process; CC, cellular component; MF, molecular function; HP, human phenotype ontology; KEGG, Kyoto Encyclopedia of Genes and Genomes. *Terms mentioned in the results section.

SupFig 40 - Related to Fig7

a

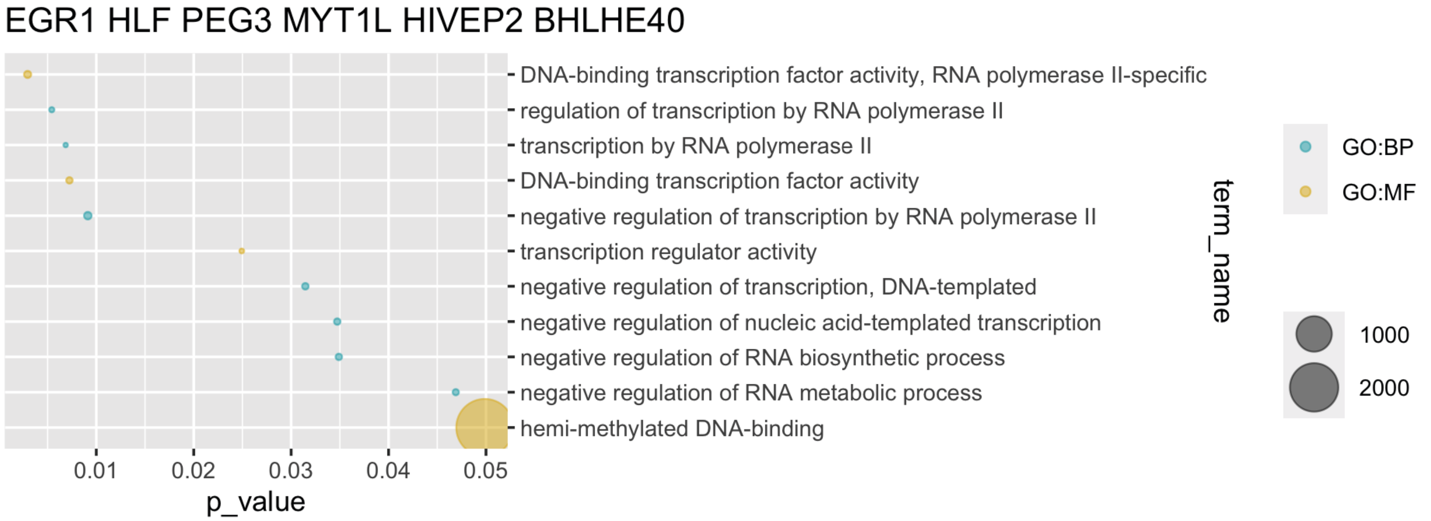

b

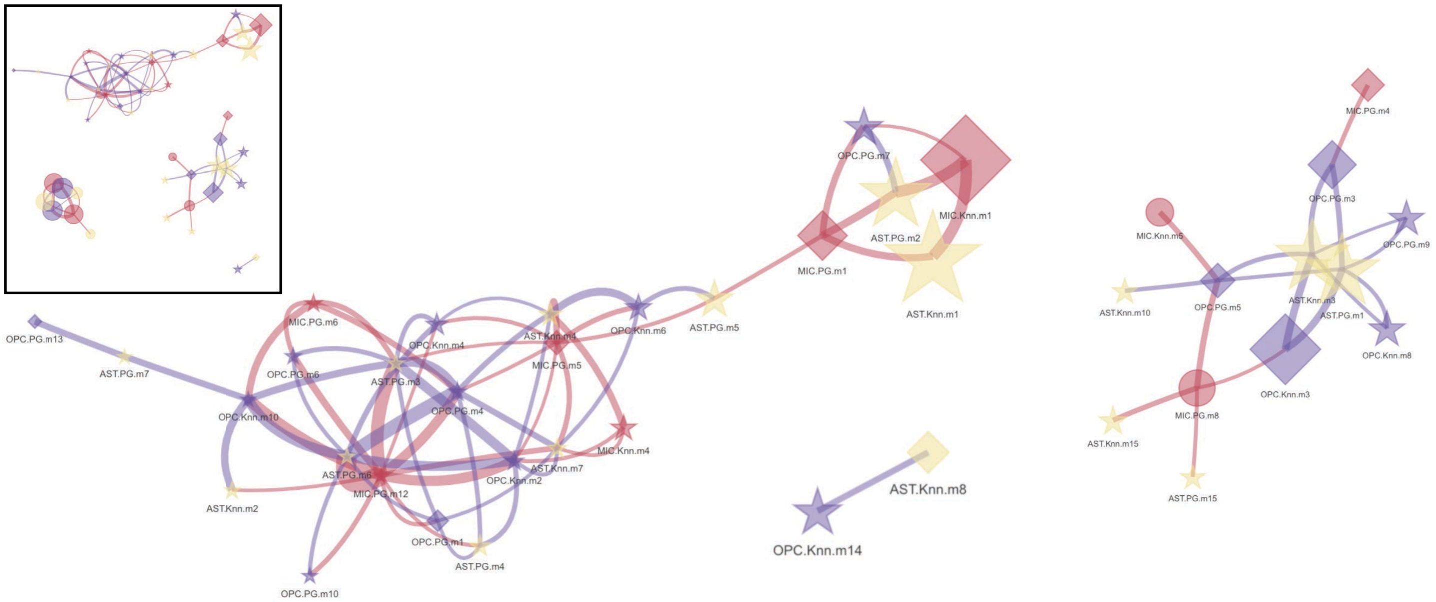

Supplementary Fig. 40. Related to Fig. 7. Gray matter and white matter glia are distinguished by their shared regulatory pathways.

a. Dot plot showing the enriched GO terms from the 6 shared transcription factors that are enriched in GM-glia. GM-glia are enriched with regulatory pathways that negatively impact RNA biosynthesis, consistent with the lower number of genes detected in GM- glia compared to WM-glia.

b. Network plot showing the similarity of GO terms identified in each gene module. The size of nodes in the network represents the number of significant GO terms found in each gene module, and nodes are annotated by shape to reflect differential loading of each module (dot, gene module enriched primarily in GM-glia; diamond, gene module enriched primarily in WM-glia; star, gene module enriched in multiple and/or "other" tissues). Edges between nodes are scaled to reflect similarity between two list of GO terms: thicker edges indicate greater similarity between the gene modules. Only edges with Jaccard index >0.25 are shown to avoid crowding, which results in the separation into 3 distinct networks and 1 connection. Upper-left, overall network structure in scale. Lower-right, networks and connection enlarged to see the module labeling of each node, not in scale.

SupFig 41 - Related to Fig8

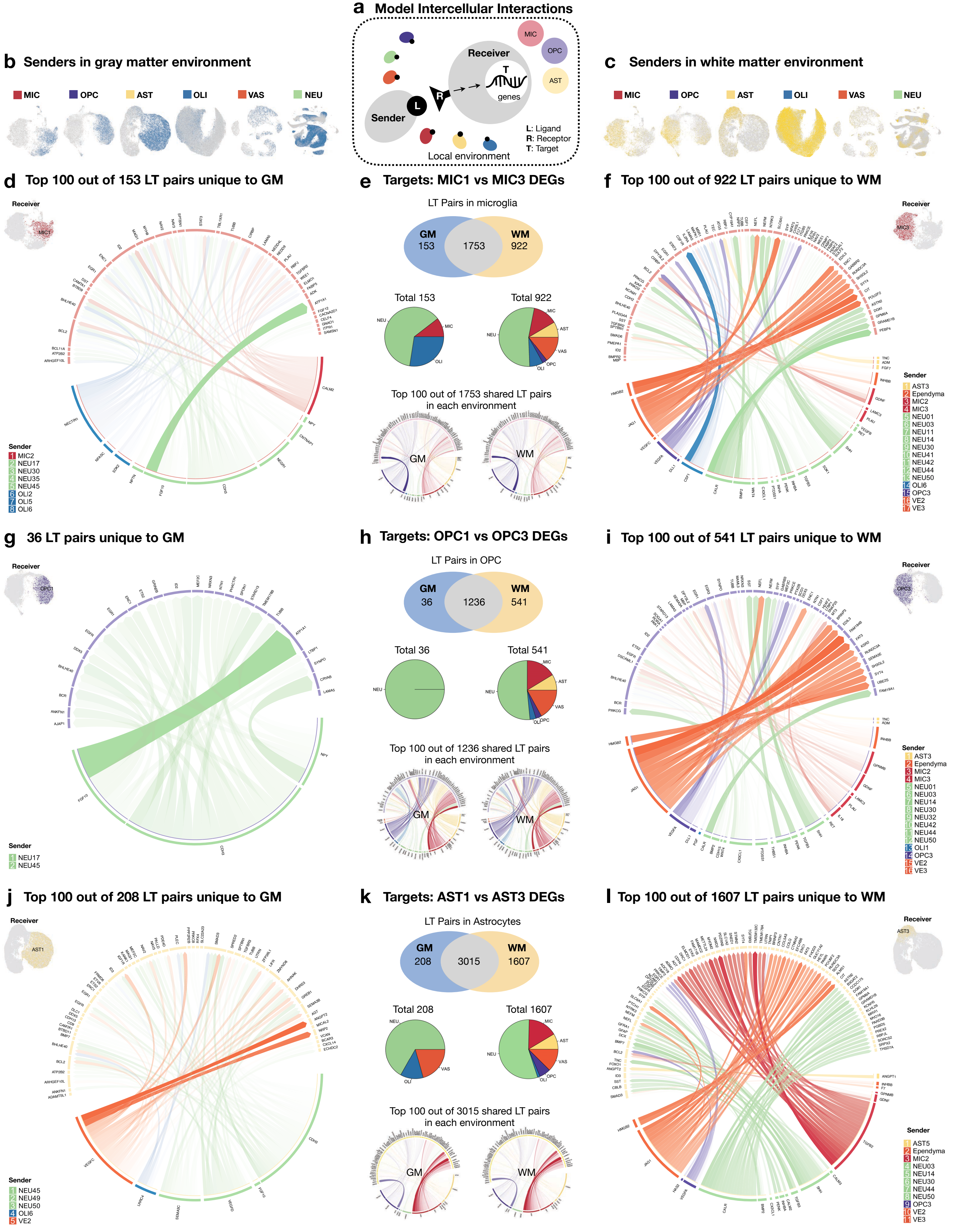

Supplementary Fig. 41. Related to Fig. 8. Interactions between resident cells are vastly more extensive in white matter than in gray matter.

a. We modeled intercellular communication in "WM" and "GM" microenvironments (see Fig. 1d for list) using a pre-established database in NicheNet, which contains information about ligand-receptor and receptor-target pairs. We aimed to identify cell types ("senders") that express ligands with matching receptors in target cells ("receivers"). To limit false positivity, we kept linked pairs only if the expression of downstream target genes also matched the receptor-target pairing information in the database. All cell classes defined in Level 1 analysis were included as senders in each microenvironment, and their influence on microglia, OPC, and astrocytes were modeled.

b. Senders present in "GM" are colored blue for each cell class.

c. Senders present in "WM" are colored yellow for each cell class.

d – f. Genes differentially expressed between MIC1 in "GM" and MIC3 in "WM" were set as target genes in the NicheNet analysis. Ligand genes expressed by senders in "GM" or "WM" (coarse category, see Fig. 1d for list) were extracted and filtered separately, and links to receptors expressed by MIC1 or MIC3 were matched and scored by NicheNet independently. The number of final established ligand-target (LT) pairs in "GM" or "WM" tissue are compared in Venn diagrams (e, top). The number of shared ligand- target pairs (1753) and the top 100 features in each environment are plotted in the center (e, bottom; see Supplementary Fig. 42 for full panel). Sender cell types (in Level 1 classes) for ligand-target pairs that are unique to each environment are further depicted in pie charts (e, middle). In the 153 ligand-target pairs that were unique to "GM," ligands were found from neurons, oligodendrocytes, and microglia (d). In the 922 ligand-target pairs that were unique to "WM," ligands were found from additional cell types (astrocytes, vascular cells, and OPC, f).

g – i. Genes differentially expressed between OPC1 in GM and OPC3 in cWM were set as target genes in the NicheNet analysis. Ligand genes expressed by senders in "GM" or "WM" (coarse category, see Fig. 1d for list) were extracted and filtered separately, and links to receptors expressed by OPC1 or OPC3 were matched and scored by NicheNet independently. The number of final established ligand-target (LT) pairs in "GM" or "WM" tissue are compared in Venn diagrams (h, top). The number of shared ligand-target pairs (1236) and the top 100 features in each environment are plotted in the center (h, bottom; see Supplementary Fig. 42 for full panel). Sender cell types (in Level 1 classes) for ligand-target pairs that are unique to each environment are further depicted in pie charts (h, middle). In the 36 ligand-target pairs that were unique to "GM," ligands were found almost exclusively from neurons (g). In the 541 ligand-target pairs that were unique to "WM,", ligands were found from additional cell types (microglia, astrocytes, vascular cells, oligodendrocytes, and OPC, i).

j – l. Genes differentially expressed between AST1 in GM and AST3 in WM were set as target genes in the NicheNet analysis. Ligand genes expressed by senders in "GM" or "WM" (coarse category, see Fig. 1d for

list) were extracted and filtered separately, and links to receptors expressed by AST1 or AST3 were matched and scored by NicheNet independently. The number of final established ligand-target (LT) pairs in “GM” or “WM” tissue are compared in Venn diagrams (k, top). The number of shared ligand- target pairs (3015) and the top 100 features in each environment are plotted in the center (k, bottom; see also Supplementary Fig. 40 for full panel). Sender cell types (in Level 1 classes) for ligand-target pairs that are unique to each environment are further depicted in pie charts (k, middle). In the 208 ligand-target pairs that were unique to “GM,” ligands were found from neurons, oligodendrocytes, and vascular cells (j). In the 1607 ligand- target pairs that were unique to “WM,” ligands were found from additional cell types (astrocytes, microglia, and OPC, l).

See also Supplementary Data 3.

SupFig 42 - Related to Fig8

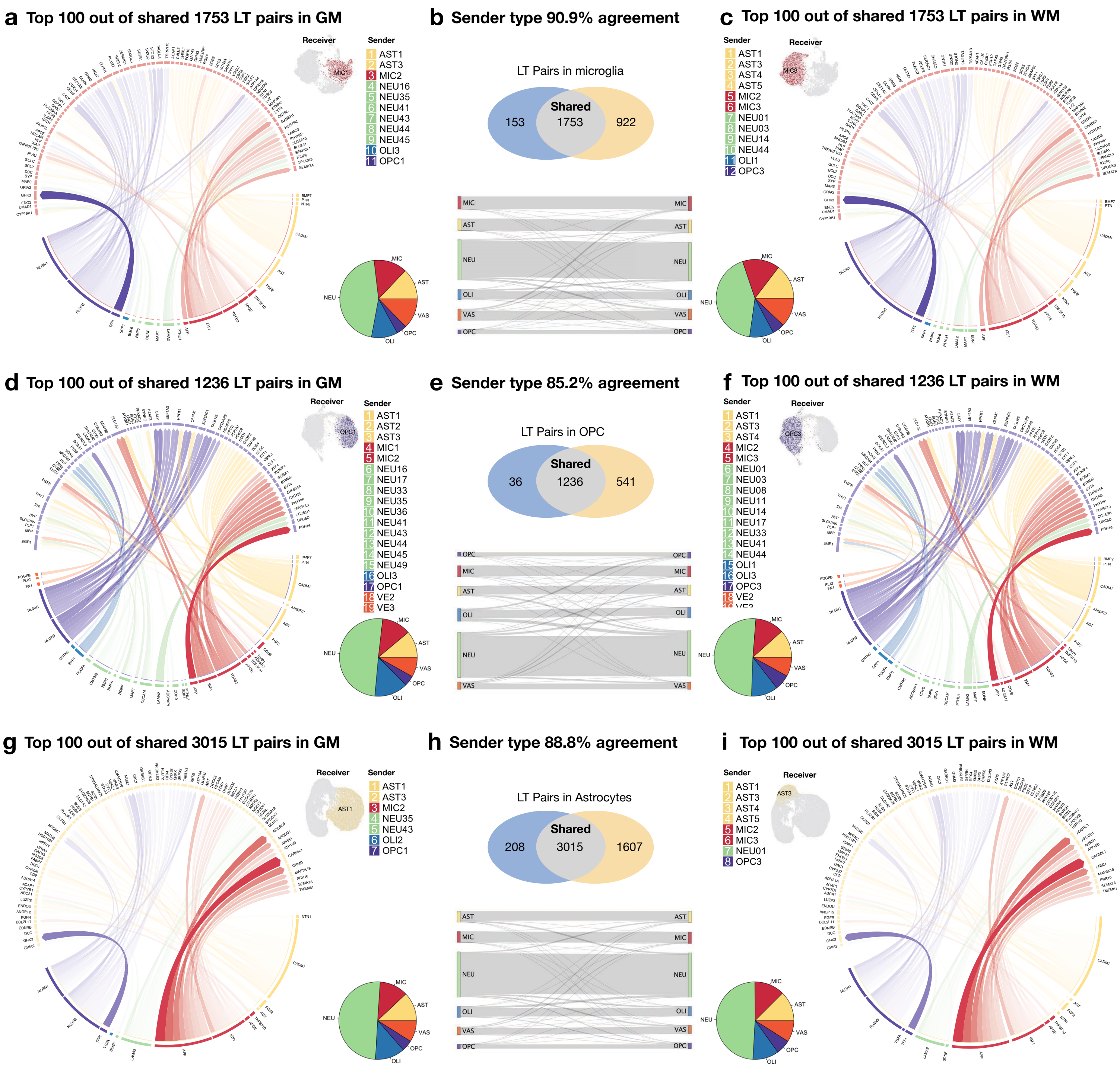

Supplementary Fig. 42. Related to Fig. 8. Shared modes of intercellular communication in "WM" and "GM."

Shared ligand-target (LT) pairs in "GM" and "WM" for MIC (**a – c**), OPC (**d – f**), and AST (**g – i**).

a, d, g. Circos plots showing the intercellular ligand-target pairs. Top 100 unique ligand- target pairs in "GM." Pie chart showing the sender contribution of shared ligand-target pairs grouped by Level 1 class in "GM."

c, f, i. Circos plots showing the intercellular ligand-target pairs. Top 100 unique ligand-target pairs in "WM." Pie chart showing the sender contribution of shared ligand-target pairs grouped by Level 1 class in "WM."

b, e, h. Top, the number of final established ligand-target pairs in "GM" or "WM" are compared in Venn diagrams. Bottom, Sankey diagram showing the agreement of sender type between "GM" and "WM."

SupFig 43 - Related to Fig9

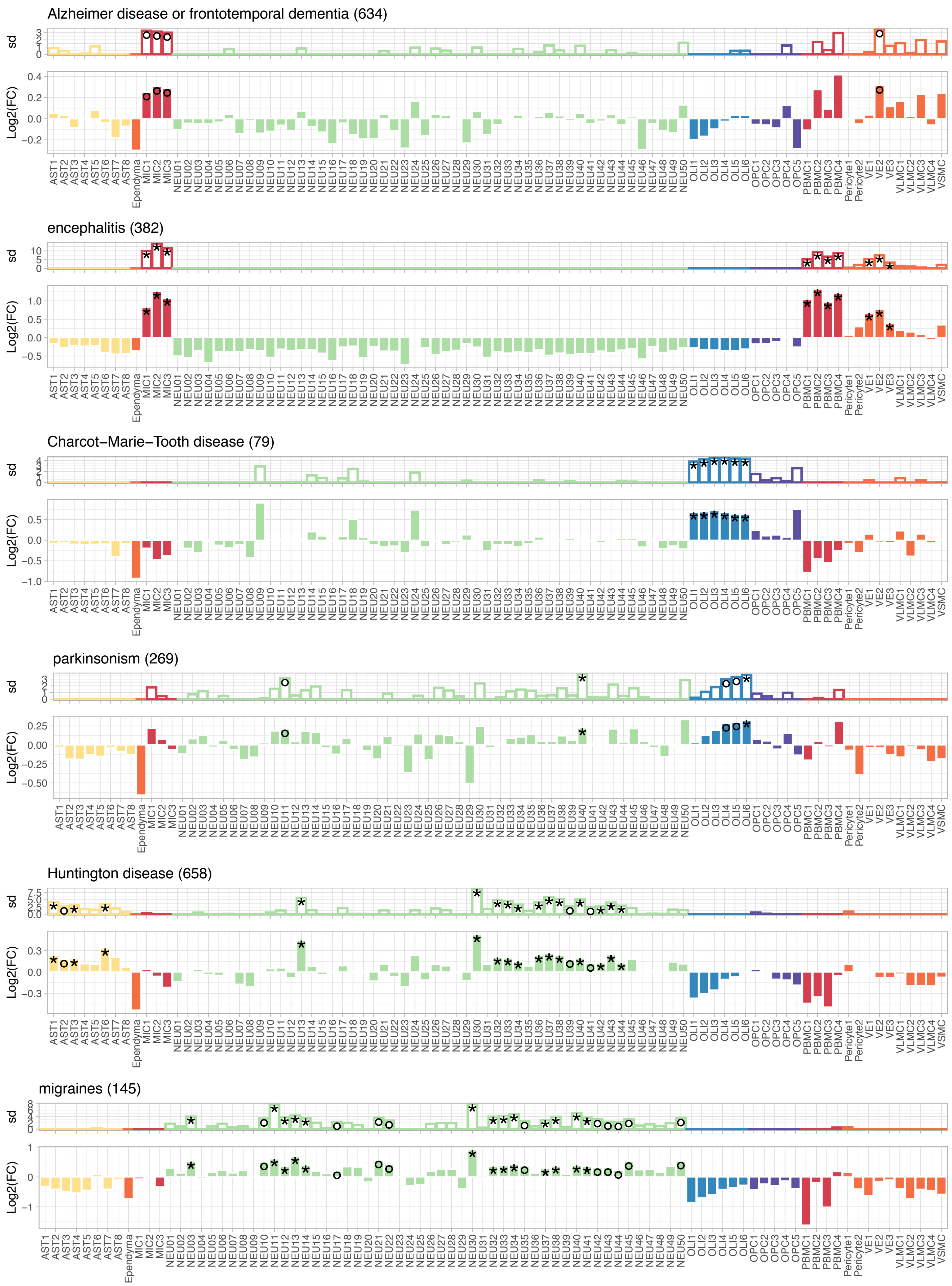

SupFig 43 - Related to Fig9 continue

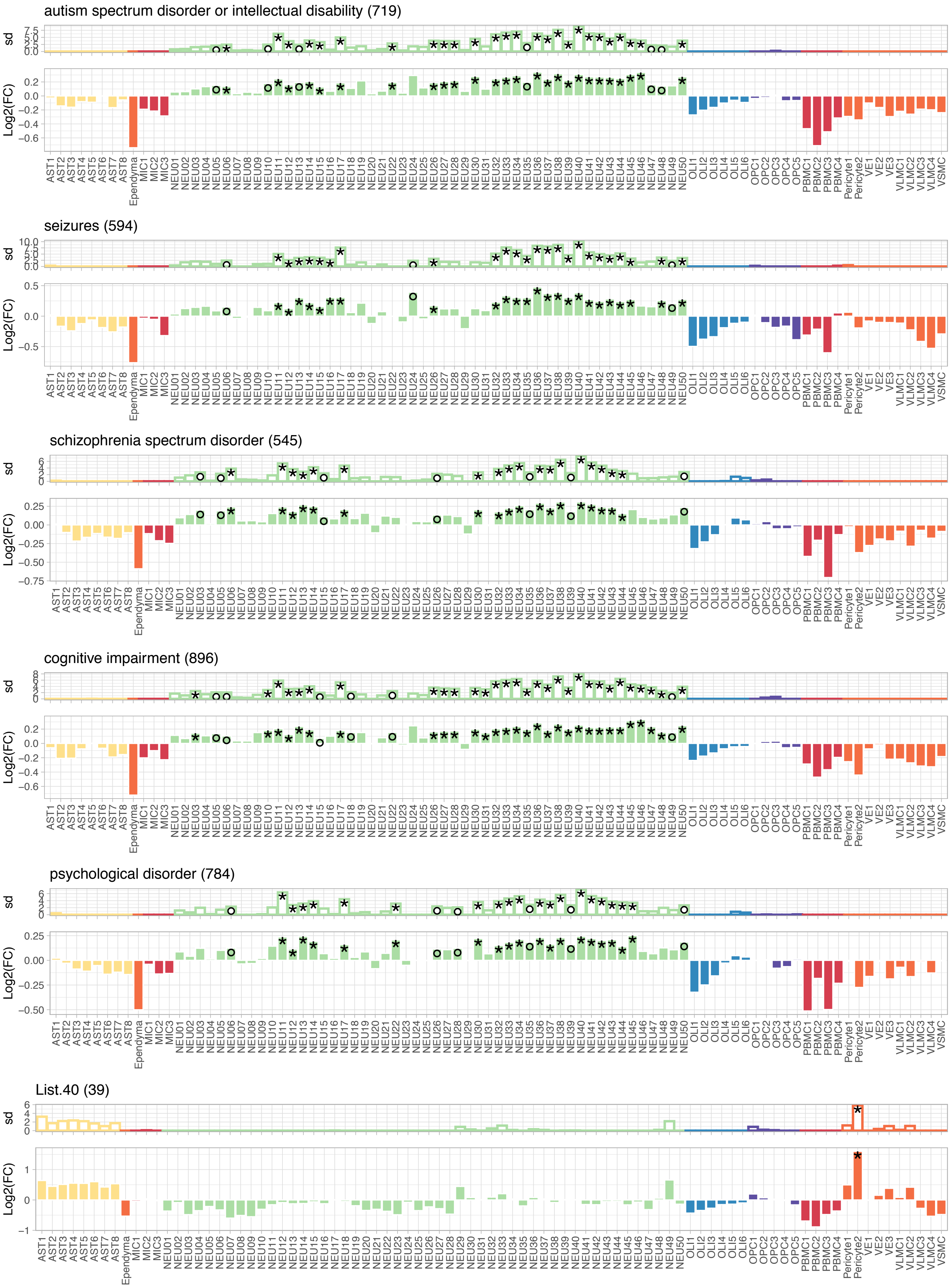

Supplementary Fig. 43. Related to Fig. 9. Cellular contributions to selected human neurological disorders

Bar graph showing standard deviation (top) and fold-change (bottom) of the enrichment probability for neurological disorder-associated genes calculated by Expression- Weighted Cell-type Enrichment (EWCE) analysis, after bootstrapping. In agreement with the view that neurovascular coupling plays a role in neurological disorders[8–10], a gene list that is uniquely shared by organic mental disorder, CNS tumor, and Huntington's disease (List.40) highlights the contribution of Pericyte2. Bar color represents the Level 1 class of origin. The significance of cell-type enrichment is denoted after Benjamini-Hochberg correction, *$q < 0.05$ and °$q < 0.1$.

Supplementary References

1. Liu, C. *et al.* A digital 3D atlas of the marmoset brain based on multi-modal MRI. *NeuroImage* **169**, 106–116 (2018).
2. Liu, C. *et al.* A resource for the detailed 3D mapping of white matter pathways in the marmoset brain. *Nat. Neurosci.* **23**, 271–280 (2020).
3. Shimogori, T. *et al.* Digital gene atlas of neonate common marmoset brain. *Neurosci. Res.* **128**, 1–13 (2018).
4. Kita, Y. *et al.* Cellular-resolution gene expression profiling in the neonatal marmoset brain reveals dynamic species- and region-specific differences. *Proc. Natl. Acad. Sci.* **118**, e2020125118 (2021).
5. Krienen, F. M. *et al.* Innovations present in the primate interneuron repertoire. *Nature* **586**, 262–269 (2020).
6. Zlokovic, B. V. The blood-brain barrier in health and chronic neurodegenerative disorders. *Neuron* **57**, 178–201 (2008).
7. Saunders, N. R., Ek, C. J., Habgood, M. D. & Dziegielewska, K. M. Barriers in the brain: a renaissance? *Trends Neurosci.* **31**, 279–286 (2008).
8. Clark, V. P., Lai, S. & Deckel, A. W. Altered functional MRI responses in huntington's disease. *Neuroreport* **13**, 703–706 (2002).
9. Iadecola, C. & Gottesman, R. F. Neurovascular and cognitive dysfunction in hypertension. *Circ. Res.* **124**, 1025–1044 (2019).
10. Grubb, S., Lauritzen, M. & Aalkjær, C. Brain capillary pericytes and neurovascular coupling. *Comp. Biochem. Physiol. A. Mol. Integr. Physiol.* **254**, 110893 (2021).
