# Supplementary material for: Transcriptomic architecture of nuclei in the marmoset CNS
Source: Nat Commun. 2022 Sep 21;13:5531. doi: 10.1038/s41467-022-33140-z (PMC9492672; doi:10.1038/s41467-022-33140-z)
Supplement: Supplementary file 3 — Description of Additional Supplementary Files [file 41467_2022_33140_MOESM3_ESM.pdf]

Description of Additional Supplementary Files

Supplementary Data 1: Sample metadata

Supplementary Data 2: Module Gene List

Supplementary Data 3: Ligand-Receptor-Target Gene List

Supplementary Data 4: Level 1, Level 2, Disease Gene List
