# Supplementary material for: Transcriptomic architecture of nuclei in the marmoset CNS
Source: Nat Commun. 2022 Sep 21;13:5531. doi: 10.1038/s41467-022-33140-z (PMC9492672; doi:10.1038/s41467-022-33140-z)
Supplement: Supplementary file 8 — Reporting Summary [file 41467_2022_33140_MOESM8_ESM.pdf]

nature portfolio

Corresponding author(s): Daniel S. Reich

Last updated by author(s): Aug 5, 2022

Reporting Summary

Nature Portfolio wishes to improve the reproducibility of the work that we publish. This form provides structure for consistency and transparency in reporting. For further information on Nature Portfolio policies, see our Editorial Policies and the Editorial Policy Checklist.

Statistics

For all statistical analyses, confirm that the following items are present in the figure legend, table legend, main text, or Methods section.

n/a	Confirmed	
☐	☒	The exact sample size (*n*) for each experimental group/condition, given as a discrete number and unit of measurement
☒	☐	A statement on whether measurements were taken from distinct samples or whether the same sample was measured repeatedly
☐	☒	The statistical test(s) used AND whether they are one- or two-sided<br>*Only common tests should be described solely by name; describe more complex techniques in the Methods section.*
☒	☐	A description of all covariates tested
☐	☒	A description of any assumptions or corrections, such as tests of normality and adjustment for multiple comparisons
☐	☒	A full description of the statistical parameters including central tendency (e.g. means) or other basic estimates (e.g. regression coefficient) AND variation (e.g. standard deviation) or associated estimates of uncertainty (e.g. confidence intervals)
☐	☒	For null hypothesis testing, the test statistic (e.g. *F*, *t*, *r*) with confidence intervals, effect sizes, degrees of freedom and *P* value noted<br>*Give P values as exact values whenever suitable.*
☒	☐	For Bayesian analysis, information on the choice of priors and Markov chain Monte Carlo settings
☒	☐	For hierarchical and complex designs, identification of the appropriate level for tests and full reporting of outcomes
☐	☒	Estimates of effect sizes (e.g. Cohen's *d*, Pearson's *r*), indicating how they were calculated

Our web collection on statistics for biologists contains articles on many of the points above.

Software and code

Policy information about availability of computer code

Data collection	see "Key Resources Table" at the Materials and Methods section
Data analysis	see "Key Resources Table" at the Materials and Methods section

For manuscripts utilizing custom algorithms or software that are central to the research but not yet described in published literature, software must be made available to editors and reviewers. We strongly encourage code deposition in a community repository (e.g. GitHub). See the Nature Portfolio guidelines for submitting code & software for further information.

Data

Policy information about availability of data

All manuscripts must include a data availability statement. This statement should provide the following information, where applicable:

- Accession codes, unique identifiers, or web links for publicly available datasets
- A description of any restrictions on data availability
- For clinical datasets or third party data, please ensure that the statement adheres to our policy

Raw and processed datasets are submitted to Gene Expression Omnibus (GEO) under session GSE165578 [https://www.ncbi.nlm.nih.gov/geo/query/acc.cgi?acc=GSE165578]. Data can also be visualized at https://cjpca.ninds.nih.gov. Source data are provided with this paper. Databases and datasets used in the study are listed in the "Key resources table" also with the following accession codes and links: GSE121654 [https://www.ncbi.nlm.nih.gov/geo/query/acc.cgi?acc=GSE121654], GSE132166 [https://www.ncbi.nlm.nih.gov/geo/query/acc.cgi?acc= GSE132166], GSE75330 [https://www.ncbi.nlm.nih.gov/geo/query/acc.cgi?acc=GSE75330], SRP135960 [https://www.ncbi.nlm.nih.gov/sra/?term=SRP135960], GSE52564 [https://www.ncbi.nlm.nih.gov/geo/query/acc.cgi?acc=GSE52564], phs001836 [https://www.ncbi.nlm.nih.gov/projects/gap/cgi-bin/study.cgi?study_id=phs001836.v1.p1], GSE97930 [https://www.ncbi.nlm.nih.gov/geo/query/acc.cgi?acc=GSE97930], GSE104525 [https://www.ncbi.nlm.nih.gov/geo/query/acc.cgi?acc=GSE104525], GSE73721 [https://www.ncbi.nlm.nih.gov/geo/query/acc.cgi?

acc=GSE73721], GSE118257 [https://www.ncbi.nlm.nih.gov/geo/query/acc.cgi?acc=GSE118257], GSE180759 [https://www.ncbi.nlm.nih.gov/geo/query/acc.cgi?acc=GSE180759], Marmoset Gene Atlas [https://gene-atlas.brainminds.riken.jp/], Marmoset Brain Mapping [https://marmosetbrainmapping.org/].

Field-specific reporting

Please select the one below that is the best fit for your research. If you are not sure, read the appropriate sections before making your selection.

- [x] Life sciences
- [ ] Behavioural & social sciences
- [ ] Ecological, evolutionary & environmental sciences

For a reference copy of the document with all sections, see nature.com/documents/nr-reporting-summary-flat.pdf

Life sciences study design

All studies must disclose on these points even when the disclosure is negative.

Sample size

Our central nervous system marmoset cell atlas was generated from 2 healthy, 5.5-year-old common marmosets (Callithrix jacchus), one female (CJH01) and one male (CJR02). For each animal, 19 types of tissue were collected to generate single-nucleus suspension. A total of 42 sample preps were included in the final atlas, 22 samples from CJH01 and 20 samples from CjR02. See supplemental figure S2 for detail on how many nuclei were recovered per sample prep. Staining was done with 4 healthy 4–6-year-old marmosets, two females (CJaT01, CJaV02) and two males (CJaB03, CJaD04). We include 2 marmosets to abide by the "reduction" principle of "3Rs" in animal research but include both sexes to account for common biological diversity. There is a well-accepted, statistically justified rule-of-thumb of using at least 2 animals in nonhuman primate neuroscience studies (doi: https://doi.org/10.1101/2022.05.10.491373). Instead of considering the individual animal as an experimental entity, we aim to survey the transcriptome diversity of nuclei (n > 500,000) across brain regions pooled from 2 animals, thereby yielding reasonable statistical power in our current design. We included 2 marmosets of both sexes for ethical, practical, and biological considerations. We found and annotated clusters that are comparable between animals to reach robust conclusions.

Data exclusions

Low quality reads, putative doublets, and low quality cells were excluded for each sample. See detail standards and parameters in "Preprocessing and quality control" paragraph in the Materials and Methods section.

Replication

Instead of considering the individual animal as an experimental entity, we aim to survey the transcriptome diversity of nuclei (n > 500,000) across brain regions pooled from 2 animals, thereby yielding reasonable statistical power in our current design.

Randomization

We performed observational study instead of controlled experiment, therefore randomization and blinding are not applicable to the study.

Blinding

We performed observational study instead of controlled experiment, therefore randomization and blinding are not applicable to the study.

Reporting for specific materials, systems and methods

We require information from authors about some types of materials, experimental systems and methods used in many studies. Here, indicate whether each material, system or method listed is relevant to your study. If you are not sure if a list item applies to your research, read the appropriate section before selecting a response.

Materials & experimental systems

n/a	Involved in the study	
	x	Antibodies
x		Eukaryotic cell lines
x		Palaeontology and archaeology
	x	Animals and other organisms
x		Human research participants
x		Clinical data
x		Dual use research of concern

Methods

n/a	Involved in the study	
x		ChIP-seq
x		Flow cytometry
	x	MRI-based neuroimaging

Antibodies

Antibodies used

The following antibodies were used: mouse anti-PLP (Bio-Rad, MCA839G, 1:200), rabbit anti-IBA1 (Wako, 019-19741, 1:200), mouse anti-IBA1 (Sigma, SAB2702364, 1:100), rabbit anti-SLC15A1 (Sigma, HPA002827, 1:100), rabbit anti-OLIG2 (Chemicon®, AB9610, 1:200), rabbit anti-GFAP (Dako, Z033429-2, 1:200), PV Poly-HRP Anti-Rabbit IgG (Leica, PV6119, 1:1), PV Poly-HRP Anti-Mouse IgG (Leica, PV6114, 1:1), ImmPRESS®-AP Horse Anti-Rabbit IgG Polymer (Vector, MP-5401-50, 1:1), ImmPRESS®-AP Horse Anti-Mouse IgG Polymer (Vector, MP-5402-50, 1:1), Goat anti-Rabbit IgG (H+L) Cross-Adsorbed Secondary Antibody, Alexa Fluor 594 (Invitrogen, A-11012, 1:400).

Validation

links to reference and citation
Bio-Rad, MCA839G: https://www.bio-rad-antibodies.com/monoclonal/cow-bovine-myelin-proteolipid-protein-antibody-plpc1-mca839.html?f=purified#references
Wako, 019-19741: https://labchem-wako.fujifilm.com/us/product_data/docs/00055446_doc03.pdf
Sigma, SAB2702364: https://www.sigmaaldrich.com/US/en/product/sigma/sab2702364

Sigma, HPA002827: https://www.sigmaaldrich.com/US/en/product/sigma/hpa002827
Chemicon, AB9610: https://www.emdmillipore.com/US/en/product/Anti-Olig-2-Antibody,MM_NF-AB9610
Dako, Z033429-2: https://www.agilent.com/en/product/immunohistochemistry/antibodies-controls/primary-antibodies/glial-fibrillary-acidic-protein-(concentrate)-76683
Leica, PV6119: https://shop.leicabiosystems.com/us/ihc-ish/detection-systems/pid-powervision-poly-hrp-anti-rabbit-ihc-detection-systems
Leica, PV6114: https://shop.leicabiosystems.com/us/ihc-ish/detection-systems/pid-powervision-poly-hrp-anti-rabbit-ihc-detection-systems
Vector, MP-5401-50: https://vectorlabs.com/products/enzyme-polymer/immpress-anti-rabbit-igg-ap-kit
Vector, MP-5402-50: https://vectorlabs.com/products/enzyme-polymer/immpress-anti-mouse-igg-ap-kit
Thermo, A-11012: https://www.thermofisher.com/antibody/product/Goat-anti-Rabbit-IgG-H-L-Cross-Adsorbed-Secondary-Antibody-Polyclonal/A-11012

Animals and other organisms

Policy information about studies involving animals; ARRIVE guidelines recommended for reporting animal research

Laboratory animals	Two common marmosets (Callithrix jacchus), 5.5 years old, both sexes
Wild animals	No Wild animals were used in this study
Field-collected samples	No filed-collected samples were used in this study
Ethics oversight	All marmosets were housed and handled with the approval of the NINDS/NIDCD/NCCIH Animal Care and Use Committee (ACUC).

Note that full information on the approval of the study protocol must also be provided in the manuscript.

Magnetic resonance imaging

Experimental design

Design type	N/A
Design specifications	N/A
Behavioral performance measures	N/A

Acquisition

Imaging type(s)	structural
Field strength	7
Sequence & imaging parameters	MRI was performed on a 7-tesla Bruker system to generate a series of proton density-weighted images with a resolution of 0.15 x 0.15 x 1 mm^3 per voxel and a matrix of 213 x 160 x 36 per session (Sati et al., 2012). The images were used for volume reconstruction and anatomy identification.
Area of acquisition	whole brain

Diabetes MRI ☐ Used ☒ Not used

Preprocessing

Preprocessing software	N/A
Normalization	N/A
Normalization template	N/A
Noise and artifact removal	N/A
Volume censoring	N/A

Statistical modeling & inference

Model type and settings	N/A
Effect(s) tested	N/A

Specify type of analysis: ☐ Whole brain ☒ ROI-based ☐ Both

Anatomical location(s)	Marmoset Gene Atlas (Shimogori et al., 2018)<br>adult marmoset MRI atlas (Liu et al., 2018a; 2020)
Statistic type for inference<br>(See Eklund et al. 2016)	N/A
Correction	N/A

Models & analysis

n/a	Involved in the study
☒	☐ Functional and/or effective connectivity
☒	☐ Graph analysis
☒	☐ Multivariate modeling or predictive analysis
